# Integrating bioinformatic prediction and the “gut microbiota-inflammation-skin axis” to decipher the mechanisms of quercetin (from *Evodia rutaecarpa*) in diabetic wound healing

**DOI:** 10.3389/fimmu.2026.1755280

**Published:** 2026-02-24

**Authors:** Zhixuan Huang, Jian Liu, Xu Zheng, Xinrong Geng, Jinlong Tan, Yangwen Ai, Hui Li, Dongyue Zhou

**Affiliations:** 1 Jiangxi Province Key Laboratory of Traditional Chinese Medicine Pharmacology, Institute of Traditional Chinese Medicine Health Industry, China Academy of Chinese Medical Sciences, Nanchang, China; 2 Jiangxi Health Industry Institute of Traditional Chinese Medicine, Nanchang, China; 3 Department of Tuina, The First Affiliated Hospital of Henan University of Chinese Medicine, Zhengzhou, China; 4 Yantai Institute of Coastal Zone Research, Chinese Academy of Sciences, Yantai, Shandong, China; 5 Institute of Chinese Materia Medica, China Academy of Chinese Medical Sciences, Beijing, China

**Keywords:** bioinformatics analysis, diabetic foot ulcer, fecal microbiotatransplantation, gut–skin axis, quercetin

## Abstract

**Background:**

Diabetic foot ulcer (DFU) is a serious complication of diabetes with impaired healing. This study focused on the herbal medicine *Evodia rutaecarpa* as a case to investigate the mechanisms of diabetic wound healing via the “gut microbiota–inflammation–skin axis”. We specifically aimed to elucidate the role of its core bioactive flavonoid, quercetin (Que), whose therapeutic potential in this context remains underexplored.

**Methods:**

*In vitro*, the direct interaction between Que and HIF1α was assessed by cellular thermal shift assay, and its functional effect on the HIF1α/VEGF pathway was evaluated in a lipopolysaccharide-induced RAW264.7/HUVEC co-culture system. *In vivo*, a streptozotocin-induced diabetic rat model with full-thickness dorsal wounds was treated with Que. Wound healing rates, metabolic parameters, systemic inflammation, and gut microbiota composition were analyzed. The causal role of the gut microbiota was further tested using fecal microbiota transplantation from Que-treated donors to diabetic recipient rats, and the biological activity of resulting drug-containing serum was assessed in HUVEC and RAW264.7 cell cultures.

**Results:**

Que was identified as a principal active component of *E. rutaecarpa* with predicted binding affinity for key targets involved in inflammatory and hypoxic responses. *In vitro*, Que directly bound to and stabilized HIF1α protein and upregulated the expression of both HIF1α and VEGF in HUVECs under inflammatory co-culture conditions. In diabetic rats, Que treatment significantly accelerated wound closure, improved systemic glucose and lipid metabolism, reduced serum levels of TNF-α and IL-1β, and modulated the gut microbiota structure. FMT from Que-treated rats replicated the pro-healing effects, enhancing angiogenesis and collagen deposition in wounds, and reducing tissue inflammation. Consistently, serum derived from the FMT-Que group promoted HUVEC migration and tube formation, and attenuated the pro-inflammatory cytokine expression in RAW264.7 cells.

**Conclusion:**

This study demonstrated that Que promoted diabetic wound healing by modulating the “gut microbiota–inflammation–skin axis”, thereby reducing systemic inflammation and enhancing local angiogenesis.

## Graphical Abstract

**Figure:**
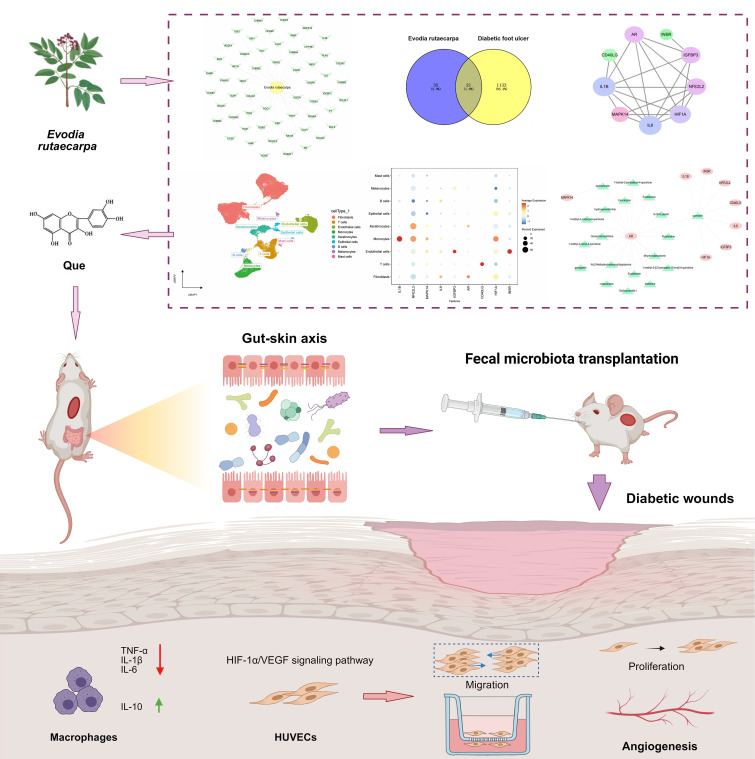
Integrating Bioinformatic Prediction and the "Gut Microbiota-Inflammation-Skin Axis" to Decipher the Mechanisms of Quercetin (from *Evodia rutaecarpa*) in Diabetic Wound Healing. The graphical abstract of this article was drawn by biorender (www.biorender.com).

## 1 Introduction

Diabetes mellitus, recognized globally as a significant public health challenge, is a metabolic disorder resulting from the complex interplay of immunological, genetic, psychological, environmental, and other contributing factors (1). As the disease progresses, persistent hyperglycemia not only disrupts systemic homeostasis but also induces chronic damage and functional impairment across various bodily tissues (2). This ultimately inflicts irreversible damage to organs and tissues, triggering a cascade of complications. Diabetic foot ulcer (DFU), a severe microvascular complication of diabetes mellitus, poses a significant global health burden because of its complex pathophysiology involving chronic hyperglycemia, impaired angiogenesis, persistent inflammation, and microbial infections (3). Current therapeutic strategies remain largely unsatisfactory, with high risks of amputation and mortality (4).

Patients with type 1 diabetes exhibit distinct alterations in their gut microbiota composition (5), accompanied by heightened activity in inflammation and immunity-related biological processes (6). The “gut–skin axis” represents a complex bidirectional communication network involving multiple physiological systems (7). Within this axis, the gut microbiota plays a pivotal regulatory role, which is primarily mediated through immunomodulatory, metabolic, and neuroendocrine pathways. On the one hand, gut microbial constituents and their metabolites can translocate to the skin via systemic circulation, thereby influencing cutaneous pathophysiological states (8). On the other hand, the gut microbiota critically modulates intestinal barrier integrity and permeability. Increased epithelial permeability may facilitate bacterial translocation, potentially triggering a systemic proinflammatory immune response that subsequently impairs skin function (9).

Traditional Chinese medicine (TCM) has garnered increasing attention for its multitarget therapeutic potential in managing diabetes-related complications (10). *Evodia rutaecarpa* is the dried nearly ripe fruit of *Euodia rutaecarpa (Juss.) Benth.*, *Euodia rutaecarpa (Juss.) Benth.* var. *officinalis (Dode) Huang*, *Euodia rutaecarpa (Juss.) Benth.* var. *bodinieri (Dode) Huang*. It has multiple pharmacological effects and is widely used in the prevention and treatment of migraine, diabetes, cardiovascular disease, cancer, and other chronic diseases (11). Studies have shown that *Evodia Fructus*-vinegar-processed *Coptidis Rhizoma* has superior efficacy to *Coptidis Rhizoma* alone in ameliorating ulcerative colitis in mice, effectively reducing colonic damage and inflammation while modulating the gut microbiota (12). In addition, the topical application *Evodia rutaecarpa* enhanced the anti-inflammatory and analgesic effects of indomethacin ointment (13). These properties render it a highly valuable research model for investigating the mechanisms through which the “gut microbiota-inflammation-skin axis” promotes diabetic wound healing.

This study aims to integrate bioinformatic prediction with experimental validation, using *Evodia rutaecarpa* as a starting point, to elucidate how this intervention influences the pathological process of diabetic wound healing, thereby providing a theoretical foundation for the development of novel therapeutic strategies.

## 2 Methods

### 2.1 Bioinformatics analysis and molecular docking

#### 2.1.1 Target screening of *Evodia rutaecarpa* intervention for diabetic foot

The drug targets of *Evodia rutaecarpa* were studied via the Traditional Chinese Medicine Systems Pharmacology Database and Analysis Platform (TCMSP) (https://tcmspw.com/tcmsp.php) (14). In addition, the OMIM database (https://www.omim.org/) (15) and GeneCards database (https://www.genecards.org/) (16) were used to screen the pathological targets of DFU. A Venn diagram was used to integrate the drug targets of Chinese medicine and the pathological targets of DFU to determine the potential mechanism of action of traditional Chinese medicine in the treatment of DFU.

#### 2.1.2 Chip data acquisition

The series matrix file of GSE147890 was downloaded from the NCBI GEO public database (https://www.ncbi.nlm.nih.gov/geo/info/datasets.html) (17), and the annotation file was GPL571. A total of 24 samples of expression spectrum data were included, including 12 cases in the control group and 12 cases in the disease group. The GSE165816 dataset from the NCBI GEO public database was downloaded, and a total of 22 datasets were included for single-cell analysis.

#### 2.1.3 Consistency clustering of drug–disease intersection genes

Consensus clustering was performed on the basis of the drug–disease intersection gene expression matrix to define DFU subtypes. The analysis involved 50 iterative computations, each utilizing a random 80% subset of the samples. The determination of the optimal cluster count was informed by the characteristics of the consensus matrix heatmap and the trajectory of the consensus cumulative distribution function curve (18).

#### 2.1.4 Gene set enrichment analysis pathway enrichment analysis

To investigate signalling pathway heterogeneity between the identified subtypes, GSEA was conducted. The background gene set was the version 7.0 annotation gene set downloaded from the MsigDB database (https://www.gsea-msigdb.org/gsea/msigdb) as the annotation gene set for the subtype pathways. Gene sets demonstrating significant enrichment (adjusted P < 0.05) in the intersubtype differential expression analysis were subsequently ranked on the basis of their normalized enrichment scores (19).

#### 2.1.5 Feature selection process of LASSO regression

LASSO is a compression estimate. It constructs a penalty function to obtain a more refined model, which compresses some coefficients and sets some coefficients to zero. Therefore, the advantages of subset shrinkage are retained, and it is a biased estimate for processing data with complex collinearity. We used the Lasso logistic regression algorithm to select features for diagnostic markers of diseases. The Lasso algorithm uses the “glmnet” package.

#### 2.1.6 Kyoto encyclopedia of genes and genomes functional annotation

The R package “ClusterProfiler” was used to perform functional annotations on important genes to explore the functional relevance of these genes fully. KEGG was used to evaluate relevant functional categories. KEGG-enriched pathways with p values and q values less than 0.05 were considered significant categories.

#### 2.1.7 Single-cell analysis

First, the expression profile was read through the Seurat package, and low-expression genes were screened out (nFeature_RNA > 200 & percent.mt <= median+3MAD & nFeature_RNA <= median+3MAD & nCount_RNA <= median+3MAD & percent.ribo <= median+3MAD); the data were standardized, homogenized, and subjected to principal component analysis (PCA). In turn, the optimal number of pcs was observed through ElbowPlot, and the positional relationship between each cluster was obtained through UMAP analysis. The clusters were annotated by marker genes, and some cells that were important for the occurrence of the disease were annotated.

#### 2.1.8 Molecular docking

For the key genes, the corresponding protein 3D structure was obtained from the RCSB PDB database (https://www.rcsb.org/), and the drug component structure was obtained from the PubChem database (https://pubchem.ncbi.nlm.nih.gov/). Molecular docking was performed via PyMOL software and AutoDock software.

#### 2.1.9 Statistical analysis

Statistical analysis was performed via R language (version 4.2.2). P < 0.05 was considered statistically significant.

### 2.2 Effects of Que on lipopolysaccharide-induced co-culture system of RAW264.7 and human umbilical vein endothelial cells

#### 2.2.1 Cell lines and cell culture

HUVECs and RAW264.7 cells were purchased from Haixing Biotechnology Co., Ltd. (Suzhou, China). HUVECs were cultured in Endothelial Cell Basal Medium supplemented with 5% fetal bovine serum (FBS), 1% Endothelial Cell Culture Supplement, and 1% penicillin/streptomycin. RAW264.7 cells were cultured in Dulbecco’s Modified Eagle’s Medium (5.5 mM D-glucose) supplemented with 10% FBS and 1% penicillin/streptomycin. Both cell lines were incubated at 37°C under 5% CO_2_ and cultured separately.

#### 2.2.2 Cytotoxicity assay

RAW264.7 cells and HUVECs were seeded into 96-well plates, respectively. After 24-h incubation with various concentrations (0, 6.25, 12.5, 25, 50, 100 μM) of Que, 50 μL of 3-(4, 5)-dimethylthiahiazo(-z-y1)-2,5-diphenyltetrazolium bromide (MTT) (Solarbio, Beijing, China) (2 mg/mL) was added per well and then incubated for 3 h. The formazan crystals were dissolved in 200 μL of dimethyl sulfoxide (DMSO) and the absorbance was read at 490 nm using a microplate reader.

#### 2.2.3 Cellular thermal shift assay

HUVECs in the logarithmic growth phase were pretreated with Que or DMSO at 37°C for 30 min. The samples were then aliquoted and subjected to a temperature gradient (38, 42, 48, 54, 62, 68°C) using a thermal cycler, followed by immediate cooling on ice. After centrifugation at 12,000 g and 4°C for 15 min, the protein expression level of hypoxia-inducible factor-1 α (HIF1α) (Wanleibio, Shenyang, China) was quantified by western blotting.

#### 2.2.4 Establishment of an *in vitro* macrophage and endothelial cell co-culture system

Based on a previously described method (20), a co-culture system was established using RAW264.7 cells and HUVECs. Specifically, HUVECs were seeded in the lower chamber of Transwell inserts, while RAW264.7 cells were placed in the upper chamber. The co−culture system was treated with LPS (100 ng/mL) or varying concentrations of Que for 24 h. After incubation, HUVECs from the lower chamber were collected, and the protein expression levels of HIF1α and vascular endothelial growth factor (VEGF) (Wanleibio, Shenyang, China) were evaluated by western blotting.

### 2.3 Effects of Quercetin on wounds in diabetic rats

Male Sprague–Dawley (SD) rats (180 ± 20 g body weight, n = 50) were purchased from Beijing Sibeifu Biotechnology Co., Ltd. (licence No. SCXK [Jing] 2019--0010). The animal study protocol was approved by the Laboratory Animal Ethics Committee of Jiangxi Health Industry Institute of Traditional Chinese Medicine (animal ethics number: 2023004). The rats were maintained under controlled environmental conditions (temperature: 22–24°C; humidity: 40–60%) with a 12 h light/dark cycle. Following a week-long acclimatization period with ad libitum access to food and water, the experiments were initiated (21).

After the SD rats were adaptively fed for one week, type 1 diabetic rat models were established by a single intraperitoneal injection of streptozotocin (STZ) (Solarbio, Beijing, China) at a dose of 55 mg/kg in fasted SD rats (22). The rats with fasting blood glucose values ≥ 16.7 mM after 72 h of STZ injection were considered successfully replicated type I diabetes models. After the rats were anesthetized with isoflurane, the dorsal skin was disinfected, and a circular segment of the dorsal skin with a diameter of 20 mm was excised to establish a full-thickness cutaneous excision wound. Que (0.5% sodium carboxymethyl cellulose prepared with normal saline) (25 mg/kg) (23) and metformin (0.5% sodium carboxymethyl cellulose prepared with normal saline) (Merck, Darmstadt, Germany) (200 mg/kg) (24) were administered by oral gavage. The sham and model groups received an equivalent volume of 0.5% sodium carboxymethyl cellulose prepared with normal saline. Serum was collected on day 7, and serum/feces were harvested on day 14. After 14 d of intervention, the rats in each group were administered an oral gavage of glucose (2 g/kg) (25) for the oral glucose tolerance test, and blood glucose levels were assessed at 0, 30, 60, 90 and 120 min via a glucometer (Yuyue, Jiangsu, China).

#### 2.3.1 Serum biochemical tests

At the endpoint of the 7-day or 14-day intervention period, the rats were first anesthetized with 4% isoflurane for induction, followed by maintenance of anesthesia with 2% isoflurane. After serum collection, the isoflurane concentration was increased to 5% to ensure humane euthanasia and minimize animal suffering as much as possible. Serum levels of insulin (INS), low-density lipoprotein cholesterol (LDL-C), triglyceride (TG), total cholesterol (T-CHO), tumor necrosis factor α (TNF-α), and interleukin-1β (IL-1β) were assessed according to the manufacturers’ instructions for commercial kits (Jiancheng, Nanjing, China).

#### 2.3.2 Histopathological analysis

The wounds were photographed via a digital camera, and the wound healing rate was calculated by measuring the wound area with ImageJ software. The reduction in wound size was calculated via the following equation: reduction in wound size (%) = (A_0_ − A_t_)/A_0_ × 100%, where A_0_ is the initial wound area and A_t_ is the wound area on days 0, 3, 7, 10 and 14 after surgery. The wound area at 7 d and 14 d was considered an important indicator of wound healing (26). Wound tissues were collected on days 7 and 14 after intervention, preserved in 4% paraformaldehyde solution, dehydrated, and processed with paraffin. The solidified paraffin-embedded samples were then subjected to serial sectioning. Histopathological evaluation was performed via hematoxylin–eosin staining and Masson’s trichrome staining.

#### 2.3.3 Detection of serum via untargeted metabolomics

##### 2.3.3.1 Metabolite extraction

Metabolomic profiling was performed via a liquid chromatography–mass spectrometry (LC–MS) system consisting of a Waters Acquity I-Class PLUS UPLC coupled with a Xevo G2-XS QToF high-resolution mass spectrometer. Separation was achieved via a Waters Acquity UPLC HSS T3 column (2.1 × 100 mm, 1.8 μm). The mobile phase for both positive and negative electrospray ionization modes comprised (A) 0.1% formic acid in water and (B) 0.1% formic acid in acetonitrile. The injection volume was set at 2 μL. The mobile phase conditions for liquid chromatography are shown in **Table 1** (27).

**Table 1:** Conditions of the liquid chromatography mobile phase.

Time (min)	Flow rate (µL/min)	Mobile phase (A)	Mobile phase (B)
0.0	400	95%	5%
0.5	400	95%	5%
5.5	400	50%	50%
9.0	400	5%	95%
10.5	400	5%	95%
12.0	400	95%	5%

##### 2.3.3.2 LC–MS/MS analysis

Mass spectrometry data were acquired via a Waters Xevo G2-XS QTOF high-resolution mass spectrometer governed by MassLynx V4.2 software. The instrument was operated in MSe mode to concurrently collect both primary and secondary mass spectral data in each acquisition cycle. This setup utilized a low collision energy channel and a high collision energy channel ramped from 10 to 40 V, with a spectral scan rate of 0.2 seconds. The ESI source parameters were configured as follows: capillary voltage at 2500 V (positive) or -2000 V (negative); cone voltage at 30 V; ion source temperature at 100°C; desolvation gas temperature at 500°C; cone gas flow rate at 50 L/h; and desolvation gas flow rate at 800 L/h (28).

##### 2.3.3.3 Data preprocessing and analysis

Data processing was initiated by subjecting the raw MassLynx V4.2 data to Progenesis QI software for peak extraction, alignment, and compound identification, which leveraged both the METLIN database (https://metlin.scripps.edu) and a self-established library. After normalization of the original peak areas to the total area, initial data quality assessment was performed through principal component analysis and Spearman correlation analysis to evaluate repeatability. The annotated compounds were queried against the KEGG, HMDB, and Lipid Maps databases for functional insights. Differential analysis involved the calculation of fold changes and the application of t-tests to determine statistical significance (p value). An orthogonal partial least squares-discriminant analysis (OPLS-DA) model was constructed with the ropls R package and rigorously validated by 200 permutation tests. The variable importance in projection (VIP) values from this model were then utilized, in conjunction with the criteria of fold change (FC) > 1 and P value (P < 0.05), to screen for robust differential metabolites. Enrichment analysis of relevant KEGG pathways was subsequently conducted via a hypergeometric test (28).

#### 2.3.4 16S rRNA gene sequencing of the gut microbiota

Following collection into sterile cryovials from each experimental group, the preservation of fecal samples involved immediate flash freezing in liquid nitrogen (1 h) prior to transfer to a -80°C freezer. For characterization of the gut microbial community, 16S rRNA gene sequencing was performed by Biomarker Technologies Co., Ltd. (Beijing, China) (21).

### 2.4 Effects of faecal microbiota transplantation on wounds in diabetic rats

Male SD rats (180 ± 20 g body weight, n = 50) were purchased from Beijing Sibeifu Biotechnology Co., Ltd. (licence No. SCXK [Jing] 2019--0010). The diabetic rat wound model was established as described in Section 2.3. Four days prior to modelling, the donor rats were intraperitoneally administered antibiotics (Yuanye, Shanghai, China) (vancomycin 100 mg/kg, neomycin sulfate 200 5mg/kg, metronidazole 200 mg/kg, and ampicillin 200 mg/kg) to deplete the gut microbiota (29). The rats were subsequently divided into faux aseptic sham (FS), faux aseptic model (FM), faux aseptic quercetin (FQ), and faux aseptic fecal microbiota transplantation quercetin (FMQ) groups. Following the final administration, the rats were anaesthetized, and blood was collected from the abdominal aorta. The blood was allowed to stand at room temperature for 2 h and then centrifuged at 3,500 r/min for 15 min at 4°C, after which the supernatant was left undisturbed for 30 min. Subsequently, it was inactivated in a 56°C water bath for 30 min and filtered through a 0.22 μm micropore filter, and the resulting drug-containing serum was stored at -80°C.

#### 2.4.1 Effects of FMT on intestinal permeability in rats

After the intestinal tissues of the rats were lysed, the protein concentration was quantified via a BCA assay. The expression levels of Zonula occludens-1 (ZO-1), Occludin and Claudin 1 (Proteintech, Wuhan, China) were detected via western blotting.

#### 2.4.2 Effects of FMT-derived drug-containing serum on cells

##### 2.4.2.1 Cell viability assay

Exponentially proliferating HUVECs/RAW264.7 cells, at a density of 1×10^4^ cells per well, were seeded in a 96-well plate and placed in an incubator until they reached a density of 80%. The cells were then treated with 5%/10% drug-containing serum obtained from the various FMT experiment groups for 24 h. Cell viability was determined via the MTT assay.

##### 2.4.2.2 Reactive oxygen species level of cells

After treatment, the culture medium of the HUVECs was aspirated. The cells were then incubated with 2’,7’-dichlorodihydrofluorescein diacetate (DCFH-DA) diluted in serum-free medium at 37°C for 30 min in the dark. Following incubation, the cells were thoroughly washed three times with prewarmed serum-free medium and subsequently ready for observation under a fluorescence microscope.

##### 2.4.2.3 Cell migration assay

HUVECs were seeded into 6-well culture plates and cultured until a confluent monolayer formed. Scratch wounds were generated in the cell monolayers via a sterile 200 μL pipette tip, followed by two washes with PBS to remove detached cells and debris. The wounded monolayers were then coincubated with or without the drugs under standard culture conditions. Images of the cells were captured at specific time points via an inverted fluorescence microscope (DMi8, Leica, Germany), and the scratch wound healing rate was quantitatively analyzed with ImageJ software. Transwell chambers (Corning, NY, USA) were placed in a 24-well plate, with 600 μL of medium containing 10% FBS added to the lower chambers. The upper chambers received 200 μL of serum-free cell suspension with 1×10^4^ HUVECs in drug-containing or drug-free medium. After 16 h of coincubation at 37°C, the cells were fixed with 4% paraformaldehyde and stained with 0.1% crystal violet. The invasive cells on the lower membrane surface were imaged via an optical microscope.

##### 2.4.2.4 Angiogenesis assay

On the day prior to the experiment, the Matrigel^®^ matrix (Mobiotech, Xiamen, China) was thawed at 4°C overnight to ensure homogeneous dissolution. On the experimental day, prechilled 24-well plates were loaded with 150 µL of liquefied Matrigel^®^ per well and polymerized at 37°C for 1 h to establish a three-dimensional matrix for cell adhesion and angiogenesis. The cell suspensions were adjusted to a density of 1×10^5^ HUVECs per well and seeded onto the solidified Matrigel^®^ surface. The plates were incubated for 4 h at 37°C to facilitate cellular adhesion and initial tubular network formation. After incubation, the angiogenic structures were visualized via an inverted optical microscope.

##### 2.4.2.5 Real-time quantitative polymerase chain reaction of cells

Following the isolation of total RNA from HUVECs and RAW264.7 cells, complementary DNA was synthesized via reverse transcription (Servicebio, Wuhan, China). The internal reference utilized in the analysis was β-actin, and the relative expression of the target gene was determined by the 2^-ΔΔCq^ method. The analyses quantified the expression of VEGF and HIF1α in HUVECs and that of TNF-α, IL-1β, interleukin-6 (IL-6), and interleukin-10 (IL-10) in RAW264.7 cells. All primer sequences utilized in this study are listed in **Table 2** (21) and **Table 3**.

**Table 2:** Primer sequences.

Gene (Rat) for tissues of animals	Primer sequences (5′ → 3′)
β-actin	Forward: GTCAGGTCATCACTATCGGCAAT
	Reverse: AGAGGTCTTTACGGATGTCAACGT
IL-1β	Forward: CATCCAGCTTCAATCTCAC
	Reverse: ACCACTTGTTGGCTTATGTT
IL-6	Forward: CTAGGAAGAACTGGCAATAT
	Reverse: AAACCATCTGGCTAGGTAAGA
IL-10	Forward: CAGAACAGAACAGGAGAGTGGAGTG
	Reverse: GAGGGAGTGGAGGTGTGCTACTGGG
TNF-α	Forward: ACGTCGTAGCAAACCACCAA
	Reverse: CTGGGAGTAGATAAGGTACA
HIF1α	Forward: CTTGGAAACGAGTGAAAGGATACA
	Reverse: GGTTTCTGCTGCCTTGTATGG
VEGF	Forward: GCACTGGACCCTGGCTTTACT
	Reverse: AACTTCACCACTTCATGGGCTTT

**Table 3:** Primer sequences.

Gene (Mouse) for RAW264.7 cells	Primer sequences (5′ → 3′)
β-actin	Forward: CGTGGGCCGCCCTAGGCACCA
	Reverse: TTGGCCTTAGGGTTCAGGGGGG
IL-1β	Forward: GAAATGCCACCTTTTGACAGTG
	Reverse: TGGATGCTCTCATCAGGACAG
IL-6	Forward: CCCCAATTTCCAATGCTCTCC
	Reverse: CGCACTAGGTTTGCCGAGTA
IL-10	Forward: GCTCTTACTGACTGGCATGAG
	Reverse: CGCAGCTCTAGGAGCATGTG
TNF-α	Forward: ACCCTCACACTCACAAACCA
	Reverse: ATAGCAAATCGGCTGACGGT

#### 2.4.3 Effects of FMT on wound healing in diabetic rats

The wound images of each group were taken and recorded at 0, 3, 7, and 14 days. ImageJ software was used to measure and calculate the wound healing rate. Wound tissues from the rats were collected for histological analysis via hematoxylin–eosin staining, Masson’s trichrome staining, and cluster of differentiation 31 (CD31) immunohistochemical staining, followed by microscopic examination to evaluate remodelling and pathological changes. RNA was also extracted from these tissues to analyze the mRNA expression levels of VEGF, HIF1α, and IL-6 (**Table 4**). The expression levels of VEGF and HIF1α were detected via western blotting.

**Table 4:** Primer sequences.

Gene (Human) for HUVECs	Primer sequences (5′ → 3′)
β-actin	Forward: CACCCAGCACAATGAAGATCAAGAT
	Reverse: CCAGTTTTTAAATCCTGAGTCAAGC
HIF1α	Forward: TGATTGCATCTCCATCTCCTACC
	Reverse: GACTCAAAGCGACAGATAACACG
VEGF	Forward: GGAGGGCAGAATCATCACGA
	Reverse: GCTCATCTCTCCTATGTGCTGG

### 2.5 Statistical analysis

Each experiment was independently repeated at least three times. The data represent the mean ± SD. We used GraphPad Prism 10.1.2 for all the statistical analyses, with the mean values compared via one-way ANOVA.

## 3 Results

### 3.1 Potential active ingredients and targets of *Evodia rutaecarpa* for the treatment of DFU

**Figure 1:**
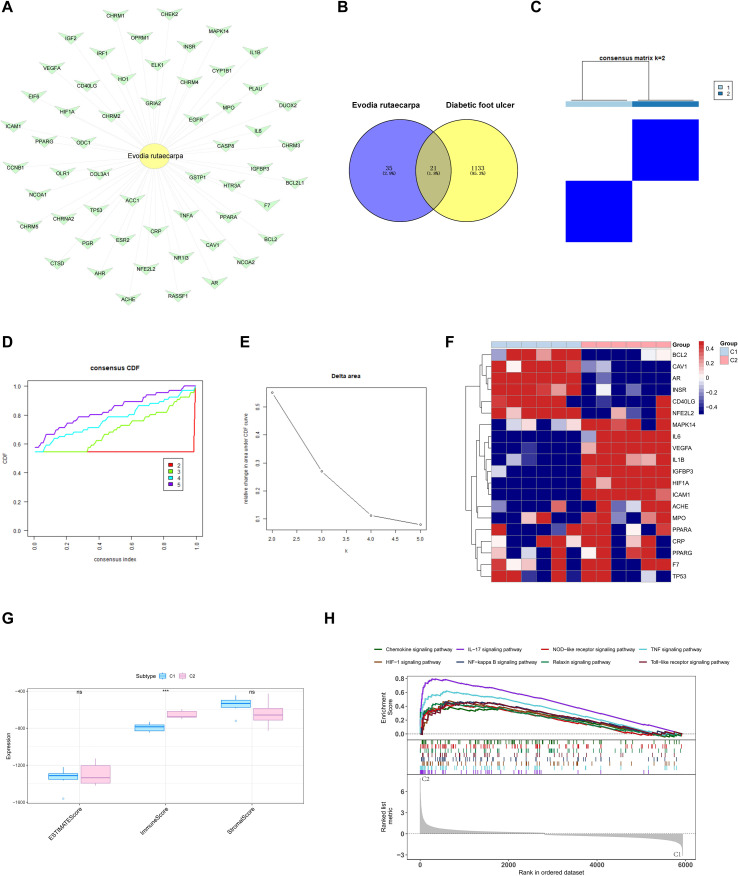
Integrated pharmacological profiling and multiomics cluster analysis of drug targets with immune enrichment signatures. **(A)** Targets of drug ingredients. **(B)** Venn diagram. **(C)** Clustering heatmap. **(D)** Clustering analysis cumulative distribution curve. **(E)** Area under the curve of the cluster analysis distribution. **(F)** ssGSEA enrichment heatmap of clusters. **(G)** Cluster immune infiltration score difference map. **(H)** Cluster GSEA enrichment plot.

The network pharmacology method was used to help determine the effect of *Evodia rutaecarpa* on DFU. This study first used the TCMSP database, which is based on the *Evodia rutaecarpa* and has a bioavailability ≥ 30% and a drug likeness ≥ 0.18, as the threshold for screening to obtain the active ingredients of the medicine and the targets on which the active ingredients act. Finally, a total of 56 corresponding drug targets were obtained, and Cytoscape was used to visualize the relationships between the Chinese herbal ingredients and the targets in the form of a network diagram (**Figure 1A**). Then, through the GeneCards database, 575 genes with relevance scores > 10 were extracted to explore the related targets of DFU, and 610 diabetic foot ulcer targets were obtained from the OMIM database. After removing duplicate values, a total of 1154 disease targets were obtained. The disease targets were subsequently intersected with the 56 drug targets of the former, and 21 intersection targets were obtained (**Figure 1B**).

To explore whether the molecular typing of drug–disease intersection genes can explain the heterogeneity of patients with DFU, we downloaded the GSE147890 DFU-related dataset from the GEO database and included the expression profile data of 24 samples, including the control group (n = 12) and the disease group (n = 12). We further used the consistency clustering method and molecularly typed the disease group on the basis of the expression of drug–disease intersection genes (**Figures 1C–E**). The results showed that when K = 2, the boundary between the two subtypes of the sample was clearer, so the DFU was divided into two clusters. Moreover, we generated a heatmap to display the expression of drug–disease intersection genes in the two subtypes (**Figure 1F**). Then, we scored the immune infiltration of the two subtypes according to the estimation algorithm and determined the difference in expression between the subtypes. The immune score was significantly different between the subtype groups (**Figure 1G**). In addition, we further analyzed the differences in signalling pathways between subtypes through GSEA. The GSEA results revealed that the pathways enriched were the chemokine signalling pathway, the IL-17 signalling pathway, the NOD-like receptor signalling pathway, etc. (**Figure 1H**).

**Figure 2:**
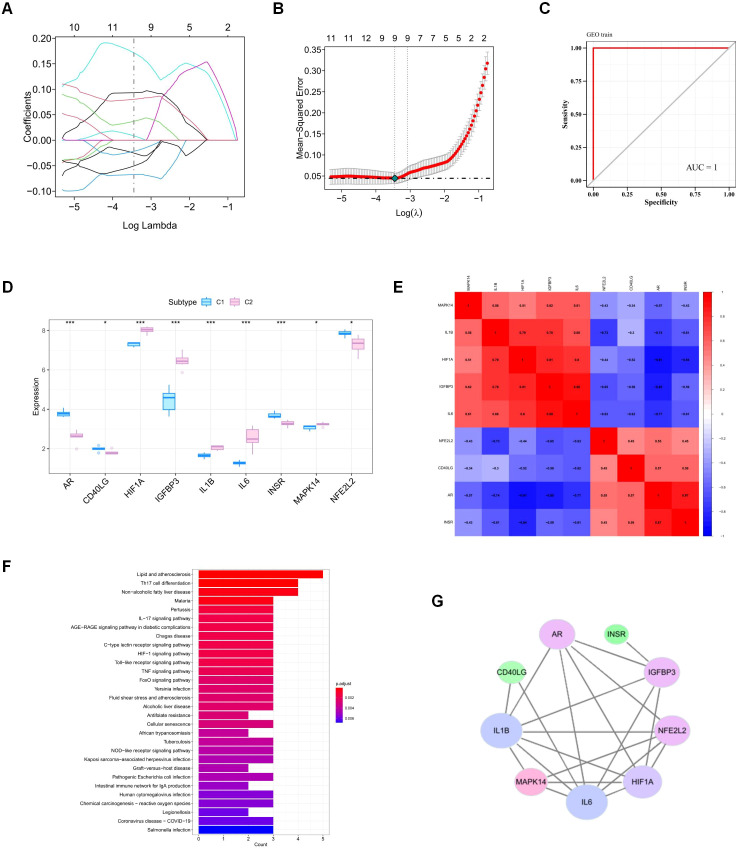
Analysis of key genes involved in DFU intervention by Evodia rutaecarpa. **(A)** LASSO regression analysis to screen feature genes. **(B)** LASSO regression λ value. **(C)** LASSO regression ROC curve. **(D)** Differences in the expression of key genes between clusters. **(E)** Heatmap of the correlations of key gene expression. **(F)** KEGG enrichment plot of key genes. **(G)** PPI network diagram.

We selected 21 disease–drug intersection genes from the GSE147890 dataset and performed feature screening through LASSO regression. The results showed that LASSO regression identified a total of 9 genes as characteristic genes of DFU (**Figures 2A, B**), as indicated by the ROC curve (AUC = 1). While the high AUC value suggests excellent separation in the training set, we recognize the potential for overfitting given the limited sample size. Therefore, these bioinformatics results were interpreted strictly as exploratory guidance for target screening rather than as a definitive diagnostic model (**Figure 2C**). Then, we subsequently performed intergroup expression difference analysis between the 9 genes identified by LASSO and found that the 9 genes presented significant differences (**Figure 2D**). In addition, we used heatmaps to show the correlations among the 9 genes (**Figure 2E**). Therefore, these 9 key genes are the key genes for our subsequent research. The R package “clusterprofiler” was used to perform KEGG pathway analysis, and the results revealed that the pathways associated with the genes were the IL-17 signalling pathway, the HIF-1 signalling pathway, the TNF signalling pathway and other signalling pathways (**Figure 2F**). We constructed a protein interaction network of common targets for 9 key genes via the STRING database (http://cn.string-db.org) and visualized it via Cytoscape (**Figure 2G**), which included 9 nodes and 21 edges in the network. We use the network analysis plug-in to count the nodes in the network graph and analyze their connectivity on the basis of the node degree. The larger the node degree is, the more biological functions the node has in the network. In the figure, the larger the node degree is, the more purple the color and the larger the graph, and vice versa, the lighter the green color and the smaller the graph.

**Figure 3:**
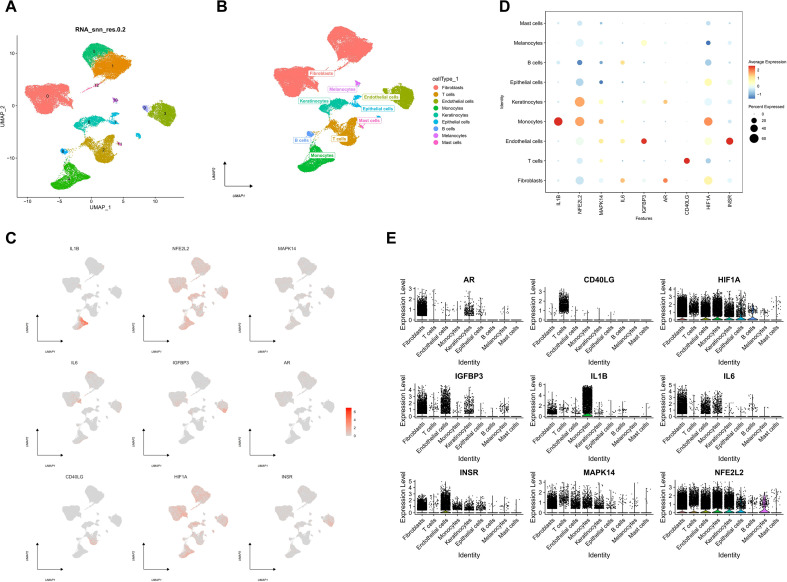
Characterization of 9 Cellular Subtypes and Key Gene Expression Profiling in scRNA-seq Data. **(A)** UMAP algorithm for cell clustering. **(B)** Annotation of each subtype. **(C)** The expression of key genes in 9 types of cells is shown in the scatter plot. **(D)** The expression of key genes in 9 types of cells is shown in a bubble plot. **(E)** The expression of key genes in 9 types of cells is shown in the violin plot.

We downloaded the single-cell data of GSE165816 and performed single-cell analysis through the Seurat package, clustered the cells via the UMAP algorithm, and obtained 13 subtypes (**Figure 3A**). Each subtype was annotated via the R package SingleR, and 13 clusters were annotated to 9 cell categories, including fibroblasts, T cells, endothelial cells, monocytes, keratinocytes, epithelial cells, B cells, melanocytes, and mast cells (**Figure 3B**). We displayed the expression of key genes in 9 cell lines in the form of scatter plots and bubble plots (**Figures 3C, D**). In addition, we analyzed the expression of the 9 key genes in each sample and displayed them in the form of violins (**Figure 3E**).

**Figure 4:**
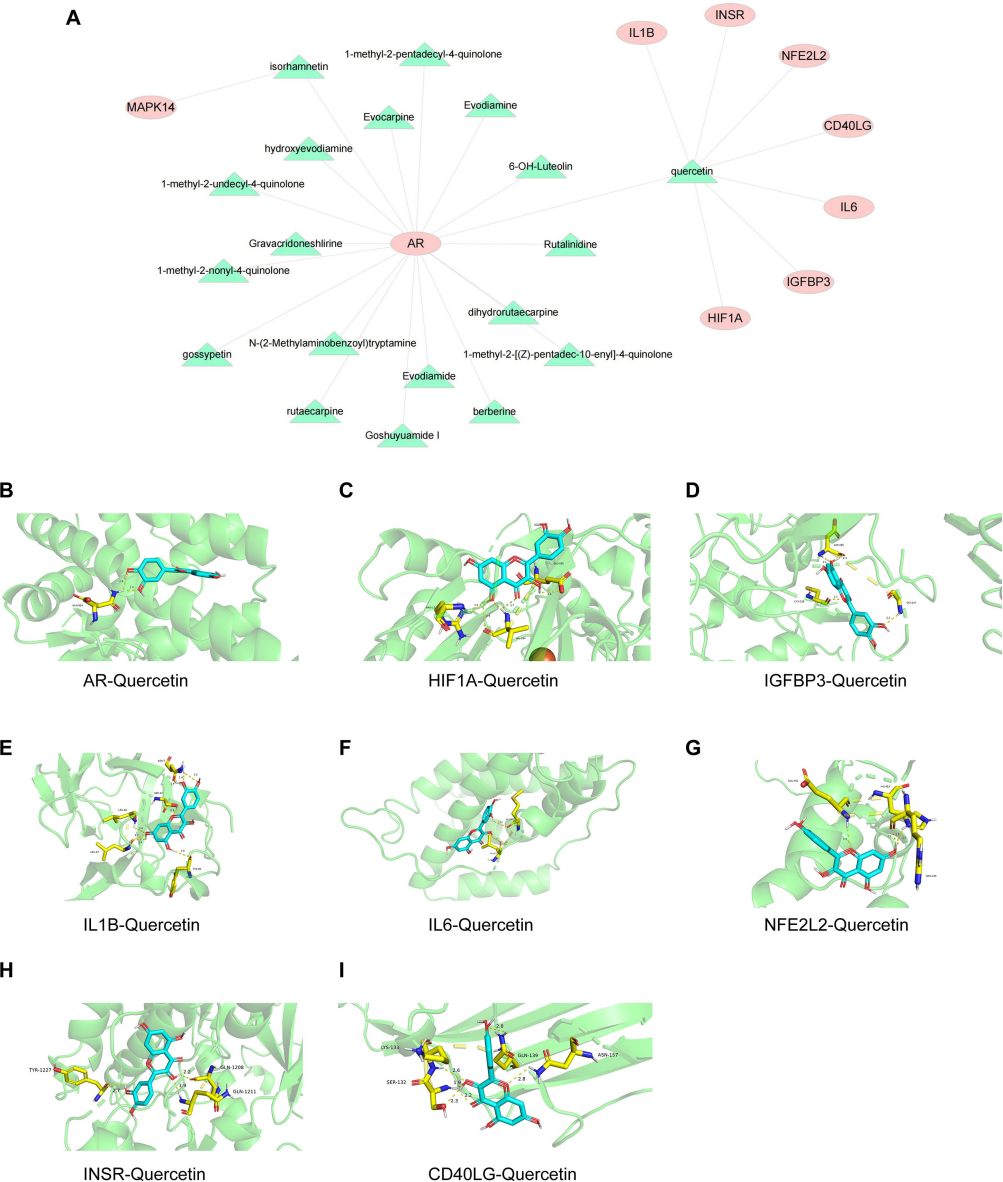
Schematic diagram of the gene target map and molecular docking. **(A)** Component–gene target map. **(B)** Molecular docking diagram of Que and AR. **(C)** Molecular docking diagram of Que and HIF1A. **(D)** Molecular docking diagram of Que and IGFBP3. **(E)** Molecular docking diagram of Que and IL-1B. **(F)** Molecular docking diagram of Que and IL-6. **(G)** Molecular docking diagram of Que and NFE2L2. **(H)** Molecular docking diagram of Que and INSR. **(I)** Molecular docking diagram of Que and CD40LG.

**Figure 5:**
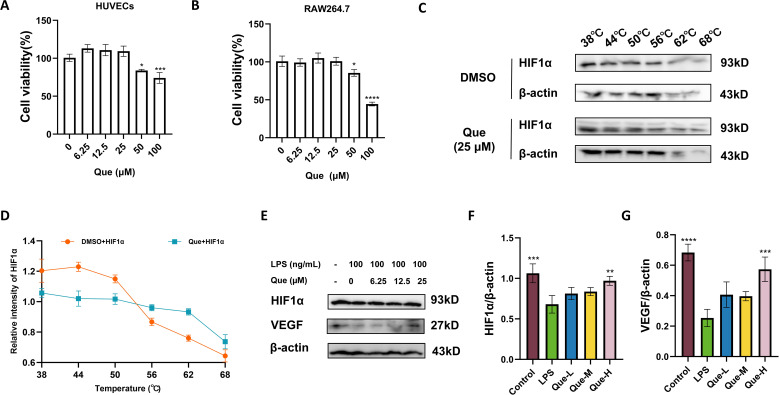
Effects of Que on LPS-induced co-culture system of RAW264.7 and HUVECs. **(A)** Cytotoxicity assessment of Que on HUVECs by cell viability assay. **(B)** Cytotoxicity assessment of Que on RAW264.7 macrophages by cell viability assay. **(C)** Representative Western blot images from CETSA. **(D)** Quantitative analysis of CETSA results. **(E)** Representative Western blot images showing protein levels of HIF-1α and VEGF in LPS-stimulated RAW264.7 and HUVEC co-culture system treated with Que at indicated concentrations. **(F)** Quantitative analysis of HIF-1α protein expression normalized to β-actin. **(G)** Quantitative analysis of VEGF protein expression normalized to β-actin. (All data are expressed as the mean ± SD (n = 3), *P < 0.05, **P < 0.01, ***P < 0.001, ****P < 0.0001. **(A, B)** vs. Control group; **(E–G)** vs. LPS group.).

We extracted the compounds corresponding to the 9 key genes on the basis of the target component of the previous drug and displayed them in the form of a Cytoscape (**Figure 4A**). Among these genes, Que had the highest correlation with the 9 key genes. The results revealed that the binding energy of the 9 target genes with Que was less than -5 kcal/mol and that the receptor and ligand could spontaneously bind and have strong binding activity (**Figures 4B-I**, **Table 5**). To validate the bioinformatic predictions, we employed cellular models. The cytocompatible concentration range of Que was first determined in both RAW264.7 cells and HUVECs (**Figures 5A, B**). Based on this assessment and previous literature (30), a concentration of 25 μM Que was selected for CETSA. The CETSA results confirmed that Que directly binds to and stabilizes HIF1α in HUVECs (**Figures 5C, D**). Furthermore, within a LPS-stimulated macrophage-endothelial co-culture system, Que was found to enhance the expression of both HIF1α and its downstream target VEGF (**Figures 5E-G**). It is well established that diabetic wounds are characterized by persistent inflammation, which contributes to impaired healing (31). Based on these findings, our subsequent investigation into the mechanism of action primarily focused on the modulation of inflammatory responses and the promotion of wound repair.

**Table 5:** Binding energies of Que and core targets.

Core target	AR	HIF1A	IGFBP3	IL1B	IL6	NFE2L2	INSR	CD40LG
Binding energy (kcal/mol)	-6.8	-7.4	-7.3	-7.3	-6.6	-6.2	-7.3	-6.0

### 3.2 Que accelerated wound healing in diabetic rats

#### 3.2.1 Effects of Que on body weight, biochemical parameters and wound tissue pathology in rats

**Figure 6:**
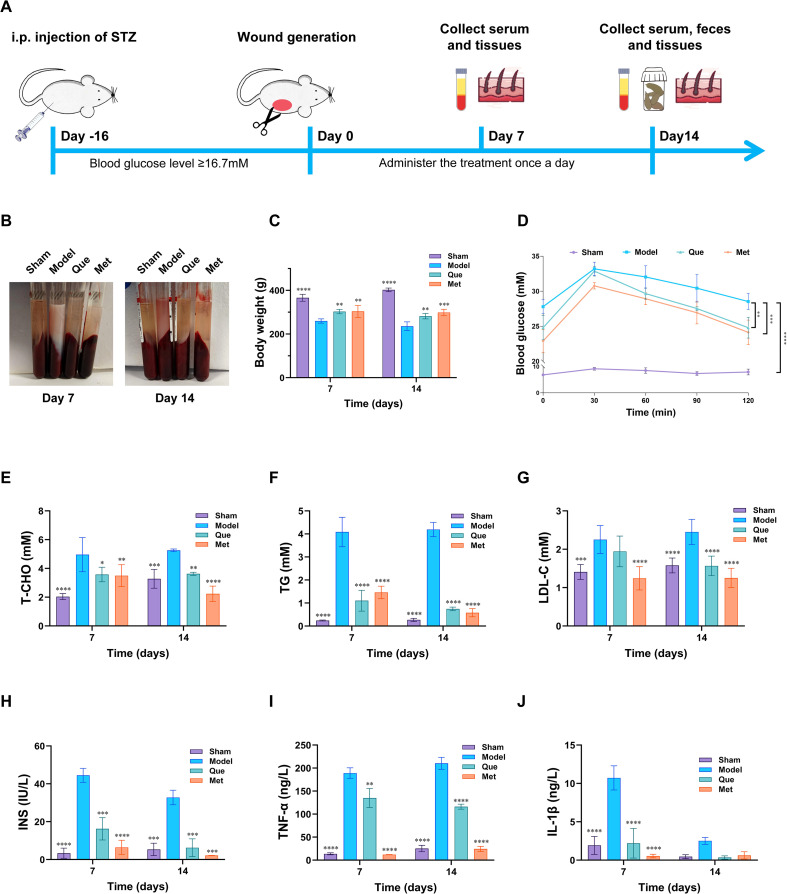
Effects of Que on the serum biochemical indices of diabetic wound-bearing rats. **(A)** Timeline of the rat experiment. **(B)** Representative pictures of the serum of each group of rats. **(C)** Weights of the rats in each group. **(D)** OGTTs of the rats in each group. **(E)** T-CHO levels in the serum of the rats in each group. **(F)** TG levels in the serum of the rats in each group. **(G)** LDL-C in the serum of the rats in each group. **(H)** INS in the serum of the rats in each group. **(I)** T-CHO levels in the serum of the rats in each group. **(J)** IL-1β levels in the serum of the rats in each group. (All data are expressed as the mean ± SD (n = 6), *P < 0.05, **P < 0.01, ***P < 0.001, ****P < 0.0001, vs. Model).

To investigate the potential impact of Que on the progression of chronic diabetic wound healing, we used type 1 diabetic SD rats to further evaluate wound healing rates and quality (**Figure 6A**). As shown in **Figures 6B, C**, on the 7th and 14th days, compared with the sham group, the model group exhibited a significant decrease in body weight, and the upper plasma presented a milky white and turbid appearance. In contrast, Que intervention attenuated the decrease in body weight of diabetic rats, and the plasma was clear and pale yellow. Oral glucose tolerance test data revealed a rapid increase in serum glucose levels in all groups following glucose administration, peaking at approximately 30 min, followed by a gradual decrease. Compared with the sham group, the model group presented significantly elevated serum glucose levels at all time-matched time points (**Figure 6D**). Que treatment also significantly reduced T-CHO, TG, and LDL-C levels (**Figures 6E-G**) while increasing INS concentrations (**Figure 6H**). Compared with those in the untreated model groups, the levels of inflammatory cytokines, particularly TNF-α and IL-1β, were markedly lower in the Que-treated groups (**Figures 6I, J**).

**Figure 7:**
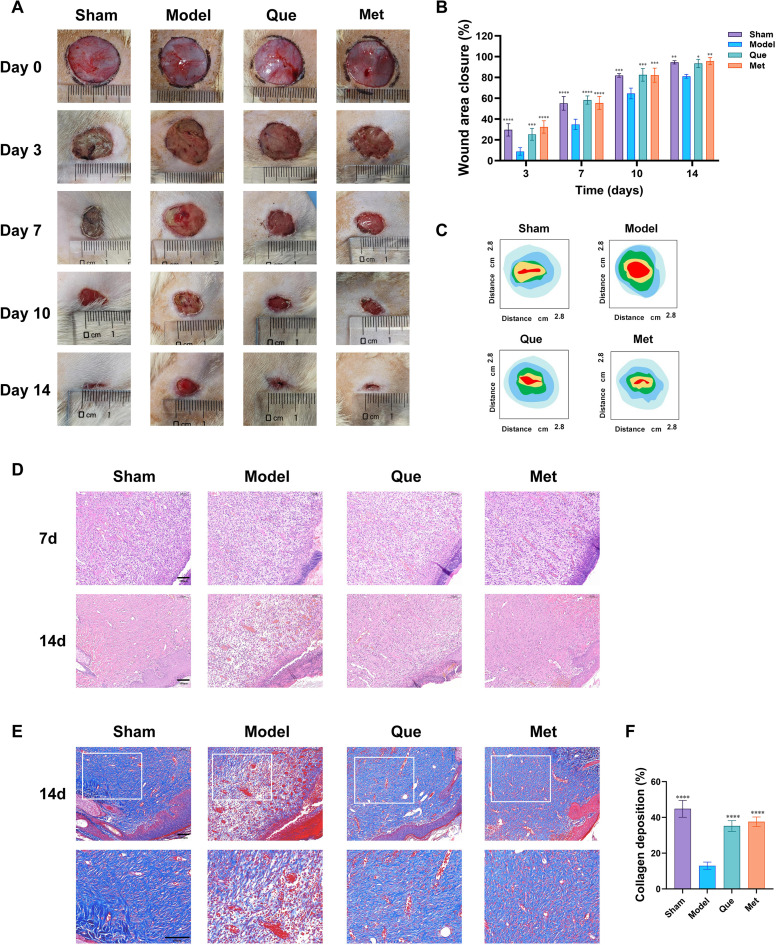
Histological evaluation of the ability of Que to promote diabetic wound healing. **(A)** Representative images depicting full-thickness skin defects in diabetic rats at various time points (0, 3, 7, 10 and 14 days) following drug administration. **(B)** Wound healing rates in each group of diabetic rats. (All data are expressed as the mean ± SD (n = 6), * P < 0.05, ** P < 0.01, *** P < 0.001, **** P < 0.0001, vs. Model) **(C)** Visualization of the wounds in each group of diabetic rats. **(D)** Hematoxylin–eosin-stained images of the wounds of diabetic rats in each group on the 7th and 14th days after surgery. **(E)** Masson-stained images of the wounds of diabetic rats in each group on the 14th day after surgery. **(F)** Quantitative analysis on collagen deposition in each group. [All data are expressed as the mean ± SD (n = 3)].

**Figures 7A-C** shows that, compared with that in the sham group, the wound healing process in the model group was prolonged, and the wound healing rate was lower. After treatment with Que, the wound healing rates improved within 14 days. To investigate the potential advantages of Que at different stages of chronic diabetic wound healing, we focused on detecting neovascularization within 7 days and collagen fibre deposition within 14 days. On day 7, diabetic wounds treated with Que displayed an increased relative number of of neovessels, occasional granulation tissue with new blood vessels, and collagen formation characterized by well-arranged fibroblasts. On day 14, the collagen fibres in the Que group were not only densely packed but also regularly arranged, indicating the initiation of the remodelling phase (**Figure 7D**). Masson staining further confirmed that, compared with the model group, the newly synthesized collagen fibers in the Que group exhibited a significantly higher density and more ordered structure (**Figures 7E, F**).

#### 3.2.2 Effects of Que on the serum metabolome of rats

**Figure 8:**
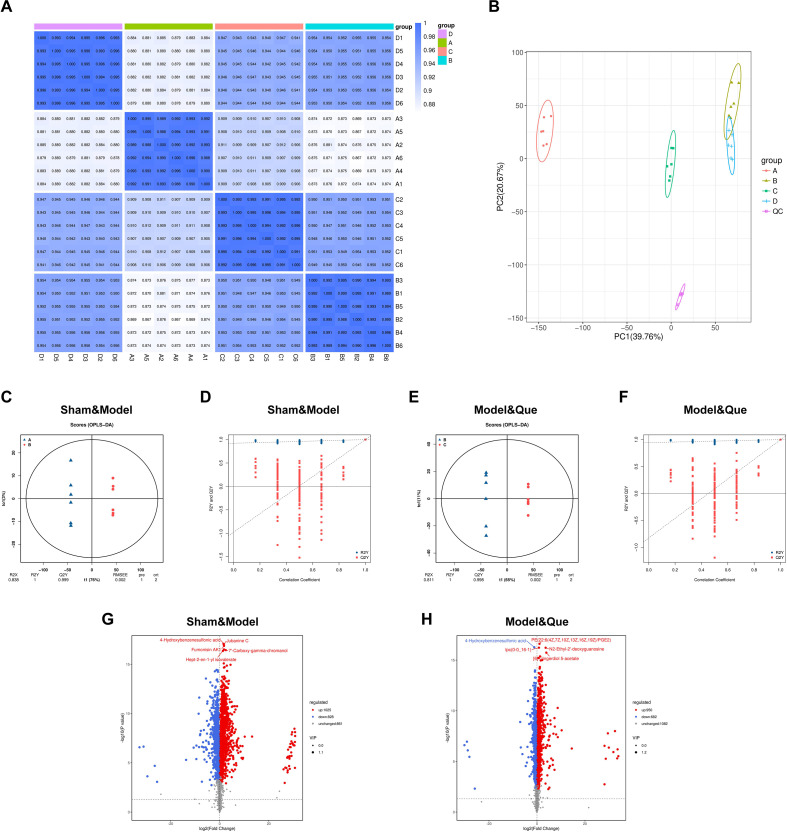
Effects of Que on the metabolism of diabetic wounds in rat serum. **(A)** Schematic diagram of the intersample correlation. **(B)** PCA statistical chart of samples. **(C)** OPLS-DA score plot of the sham group and model group. **(D)** OPLS-DA model permutation test plot of the sham group and model group. **(E)** OPLS-DA score plot of the Model group and the Que group. **(F)** OPLS-DA model permutation test plot of the Model group and Que group. **(G)** Volcano plot of the sham group and model group. **(H)** Volcano plot of the Model group and the Que group. (Sham group: A1–A6; Model group: B1–B6; Que group: C1–C6; Met group: D1–D6. Quality Control (QC) is a pooled mixture of equal aliquots from all experimental samples).

To further interpret the changes in metabolites in the serum of the rats in the disease state, the serum of the rats in each group on day 14 was analyzed via untargeted metabolomics. Spearman’s rank correlation coefficient (r > 0.8) was obtained for all pairwise sample comparisons, indicating high methodological consistency and robust biological reproducibility (**Figure 8A**). PCA revealed distinct segregation of the model group from both the sham and Que-treated groups but partial overlap with the metformin intervention group. Significant differences were observed between the Que-treated group and the model group along both the PC1 and PC2 dimensions (**Figure 8B**). OPLS-DA modelling revealed that both the sham vs. model comparison and model vs. Que-treated group comparisons presented a Q2Y > 0.5, with permutation tests demonstrating positive slopes in the corresponding validation regression lines. These parameters confirm the statistical validity and biological reliability of the OPLS-DA models for differential metabolites screening (**Figures 8C-F**). Univariate statistical analysis of all detected metabolites was performed. As demonstrated by volcano plots, significantly differential metabolites were identified between the sham and model groups, as well as between the model and Que-treated groups, under both positive and negative ionization modes (**Figures 8G, H**). In **Supplementary Table S1**, metabolites with FC ≥ 2 or ≤ 0.5, VIP ≥ 1, and P < 0.05 in the t test were selected as significantly differential metabolites. Comparative analysis among the sham, model, and Que-treated groups revealed 673 differentially abundant metabolites with significant variations in relative abundance. These included 334 metabolites detected in negative ionization mode and 339 in positive ionization mode, including 313 upregulated and 360 downregulated species.

#### 3.2.3 Effects of Que on the diversity of gut bacteria in rats

**Figure 9:**
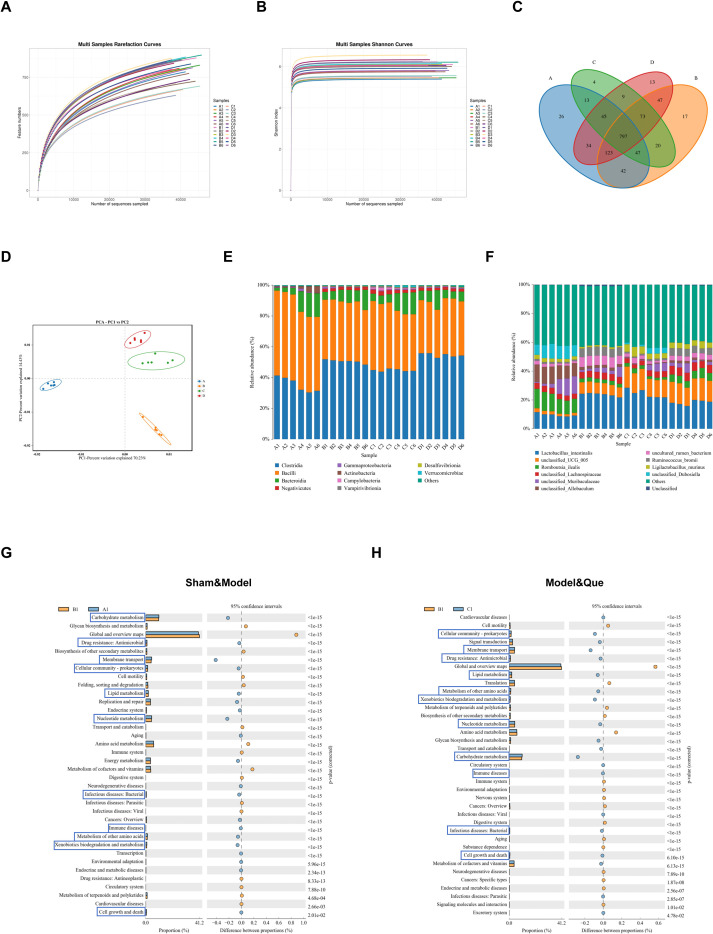
Effects of Que on the diversity of gut bacteria in diabetic wound-bearing rats. **(A)** Dilution curve of the samples. **(B)** Shannon curves of the samples. **(C)** Petal plot of the samples. **(D)** PCA statistical chart of samples. **(E)** Structural graph of samples at the class level. **(F)** Structural graph of samples at the species level. **(G)** Statistical chart of COG functional classification between the sham group (A1) and the model group (B1). **(H)** Statistical chart of COG functional classification between the Model group (B1) and the Que group (C1). (Sham group: A1–A6; Model group: B1–B6; Que group: C1–C6; Met group: D1–D6).

Owing to the important role of the gut bacteria in disease, the fecal samples of the rats were analyzed. A total of 1464722 CCS sequences were retrieved from 24 samples after sequencing and barcode identification (**Table 6**), and the alpha diversity indices were calculated via Mothur version 1.30 (**Table 7**). According to the results of the analysis, the sequencing depth of each sample was adequate and saturated, as shown by the Shannon and rarefaction analyses. Most of the microbial diversity information and the structure of the bacterial communities were reflected (**Figures 9A, B**). The Venn diagrams constructed on the basis of distinct groupings revealed that the four-way intersection contained 797 shared features (**Figure 9C**). PCA was subsequently performed on the samples, revealing distinct differences among the experimental groups. Although the Quercetin-treated group did not completely overlap with the Sham group in the PCA space, it exhibited a distinct separation from the Model group. This trajectory suggests that Quercetin intervention drives the gut microbiota towards an alternative beneficial equilibrium via structural remodeling, likely by specifically regulating key functional taxa rather than purely reversing the community structure to the naive state (**Figure 9D**). Compared with the sham group, the model group presented significantly elevated levels of *Clostridia*, *Negativicutes*, and *Desulfovibrionia* at the class taxonomic rank (P < 0.05). Notably, *Clostridia* and *Desulfovibrionia* abundances were significantly reduced following Que intervention (P < 0.05) (**Figure 9E**). At the genus level, the model group presented significantly greater abundances of *Lactobacillus*, *Ruminococcus*, *unclassified_Lachnospiraceae*, *uncultured_rumen_bacterium*, and *unclassified_Ruminococcaceae* than did the sham group (P < 0.05), whereas the abundances of *Romboutsia*, *Allobaculum*, and *Ligilactobacillus* were significantly lower (P < 0.05). Following Que intervention, a marked decrease in *Romboutsia*, *uncultured_rumen_bacterium*, and *unclassified_Ruminococcaceae* was observed (P < 0.05), accompanied by a significant increase in *Ligilactobacillus* abundance (P < 0.05) (**Figure 9F**). Further functional prediction and annotation of the 16S rRNA sequencing data were performed via PICRUSt2. Functional prediction analysis of the microbiota revealed that Que significantly modulated multiple pathways in diabetic wound rats, including those related to bacterial infections and immune regulation. This further highlights the critical role of inflammatory responses in the disease progression (**Figures 9G, H**).

**Table 6:** Statistics of the sample sequencing data processing results.

Sample ID	Raw CCS	Clean CCS	Effective CCS	AvgLen (bp)	Effective (%)
A1	57679	57619	44825	1458	77.71
A2	59293	59241	45689	1457	77.06
A3	64600	64542	49693	1457	76.92
A4	57219	57173	43850	1458	76.64
A5	61756	61709	47425	1458	76.79
A6	60011	59965	46696	1458	77.81
B1	65409	65361	48244	1463	73.76
B2	59020	59000	43632	1463	73.93
B3	61862	61811	45293	1463	73.22
B4	61440	61398	45275	1462	73.69
B5	68177	68114	49605	1463	72.76
B6	56792	56768	41245	1462	72.62
C1	59598	59558	44979	1464	75.47
C2	54839	54801	41314	1464	75.34
C3	66519	66476	49336	1464	74.17
C4	67556	67503	48622	1461	71.97
C5	57927	57881	42352	1461	73.11
C6	63937	63894	47318	1461	74.01
D1	56354	56315	40351	1460	71.6
D2	60006	59959	42488	1460	70.81
D3	59250	59182	41957	1460	70.81
D4	58427	58347	43546	1460	74.53
D5	64882	64807	47711	1461	73.54
D6	62169	62116	44841	1460	72.13

**Table 7:** Alpha diversity index statistics.

Sample ID	Feature	ACE	Chao1	Simpson	Shannon
A1	787	948.0075	947.0085	0.9389	5.3946
A2	807	966.2308	961.4	0.9421	5.4532
A3	830	999.7341	989.2	0.9426	5.4581
A4	810	985.2959	990.9076	0.9554	5.7955
A5	814	1041.9201	1043.0268	0.9565	5.8141
A6	776	949.7853	935.5537	0.956	5.7486
B1	879	1001.6018	1006.5508	0.9304	6.1007
B2	863	1026.0558	1030.6316	0.9282	6.0847
B3	878	1077.7258	1153.0333	0.9298	6.1068
B4	885	1072.8824	1086.4091	0.9323	6.142
B5	898	1059.771	1071.9279	0.9364	6.2162
B6	866	1050.3068	1098.7629	0.943	6.3331
C1	669	842.2276	850.069	0.9049	5.4624
C2	630	783.5379	763.6277	0.921	5.534
C3	692	842.907	844.6452	0.9134	5.5643
C4	724	858.5883	862.2609	0.9384	6.0093
C5	713	858.4725	852.7449	0.9403	6.0853
C6	736	955.2406	965.5109	0.9405	6.0585
D1	852	1028.4629	1039.0263	0.9489	6.2344
D2	843	983.723	1038.4045	0.952	6.332
D3	877	1022.8276	1026.9224	0.9596	6.5576
D4	804	952.5382	975.6804	0.9411	6.0653
D5	839	999.941	1048.5851	0.9393	5.9225
D6	866	1020.7502	1013.5203	0.9463	6.2232

**Figure 10:**
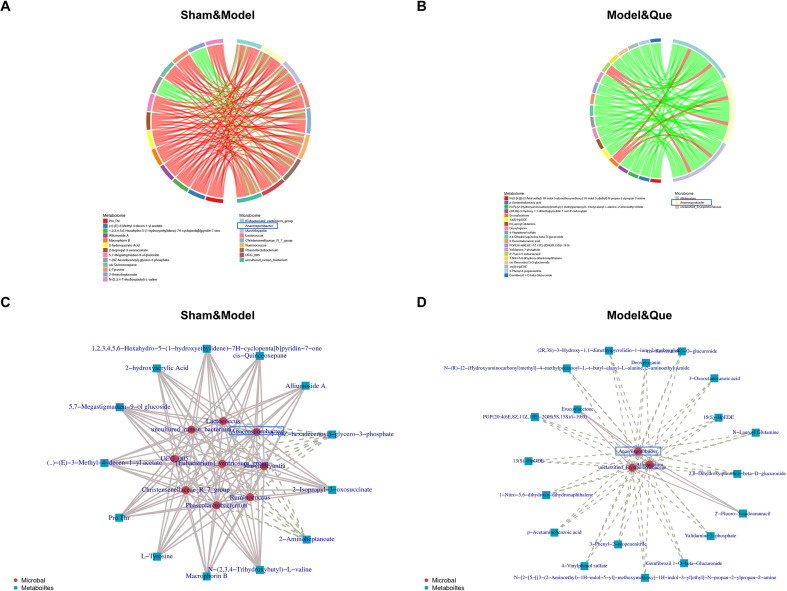
Serum metabolomics combined with microbial sequencing analysis in diabetic wound-bearing rats. **(A)** Chord diagram of the differential metabolites and differential microbes between the sham group and model group. **(B)** Chord diagram of the differential metabolites and differential microbes between the Model group and the Que group. **(C)** Differential metabolites–differential microbes network diagram of the sham group and model group. **(D)** Differential metabolites–differential microbes network diagram of the Model group and the Que group.

On the basis of the aforementioned results, we further conducted an integrated analysis of the microbiota and metabolome by correlating microbial taxa (at both the phylum and genus levels) with metabolomic data from holistic and differential perspectives. Therefore, the differential metabolites-differential microbes chord diagram (**Figures 10A, B**) and the differential metabolites-differential microbes network diagram (**Figures 10C, D**) were constructed. The results showed that after Que intervention, *Anaerosporobacter* was involved in regulating the abnormal metabolism of diseased rats.

### 3.3 Que regulated serum inflammatory factor levels and cell migration through the intestinal flora

**Figure 11:**
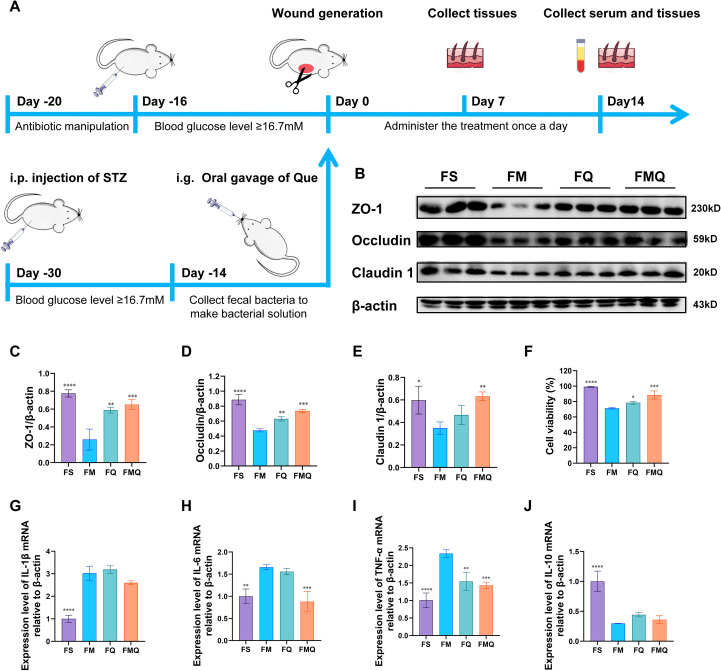
Effects of FMT on intestinal permeability in diabetic rats and effects of rat serum after FMT on Raw264.7 cells. **(A)** Timeline of the rat experiment. **(B)** The expression levels of ZO-1, Claudin 1 and Occludin in rat intestinal tissue. **(C)** Statistical analysis of ZO-1 levels in rat intestinal tissue. **(D)** Statistical analysis of Claudin 1 levels in rat intestinal tissue. **(E)** Statistical analysis of Occludin levels in rat intestinal tissue. **(F)** Effects of each group of samples on the viability of RAW264.7 cells. **(G)** The expression level of IL-1β mRNA in each group of RAW264.7 cells. **(H)** The expression level of IL-6 mRNA in each group of RAW264.7 cells. **(I)** The expression level of TNF-α mRNA in each group of RAW264.7 cells. **(J)** The expression level of IL-10 mRNA in each group of RAW264.7 cells. (All data are expressed as the mean ± SD (n = 3), *P < 0.05, **P < 0.01, ***P < 0.001, ****P < 0.0001, vs. FM).

To investigate whether Que exerts its therapeutic effects on diabetic wounds through the gut microbiota, an FMT approach was further employed (**Figure 11A**). The FBG level in FMQ group rats was significantly lower than that in the FM group (**Table 8**). As shown in **Figures 11B-E**, rats in the FMQ group presented higher protein expression levels of ZO-1, Occludin, and Claudin-1 than those in the FM group did, indicating that FMT contributed to the restoration of the intestinal barrier in diabetic rats.

**Table 8:** FBG levels in FMT Rats on Day 14.

Groups	Number	FBG (mM)
FS	6	7.47 ± 0.57****
FM	6	28.43 ± 0.81
FQ	6	24.63 ± 0.33**
FMQ	6	21.83 ± 1.13*

* P < 0.05, ** P < 0.01, *** P < 0.001, **** P < 0.0001.

To observe the effects of the FS, FM, FQ, and FMQ groups on cells, the MTT assay results revealed that, compared with the FM group, the FMQ group significantly increased the viability of RAW264.7 cells (**Figure 11F**). RT–qPCR analysis revealed that FMQ significantly upregulated IL-10 mRNA expression in RAW264.7 cells but downregulated the mRNA levels of TNF-α, IL-1β, and IL-6 (**Figures 11G-J**).

**Figure 12:**
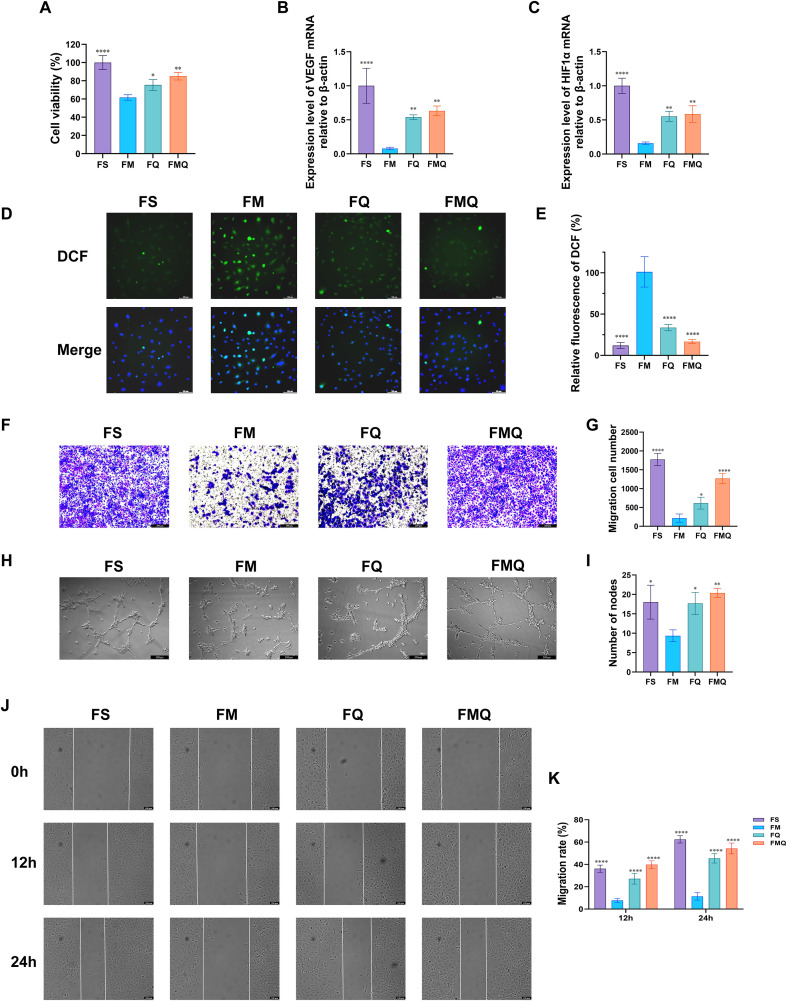
Effects of rat serum after FMT on HUVECs. **(A)** Effects of each group of samples on the viability of HUVECs. **(B)** The expression level of VEGF mRNA in each group of HUVECs. **(C)** The expression level of HIF1α mRNA in each group of HUVECs. **(D)** Images of ROS generation in each group of HUVECs. DAPI (blue) was used as a nuclear marker. **(E)** Quantitative relative fluorescence assay of DCF in each group of HUVECs. **(F)** Results of the migration ability of each group of HUVECs. **(G)** The number of invading cells in each group of HUVECs. **(H)** In vitro images of tube formation in each group of HUVECs. **(I)** The number of nodes in each group of HUVECs. **(J)** Cell scratch images of each group of HUVECs at 12 h and 24 h. **(K)** Migration rate of each group of HUVECs. (All data are expressed as the mean ± SD (n = 3), *P < 0.05, **P < 0.01, ***P < 0.001, ****P < 0.0001, vs. FM).

As shown in **Figure 12A**, compared with the FM group, the FMQ group significantly increased the viability of HUVECs. RT–qPCR analysis confirmed that FMQ significantly upregulated the mRNA expression levels of HIA1α and VEGF in HUVECs (**Figures 12B, C**). As shown in **Figures 12D, E**, ROS levels were significantly increased in the FM group, whereas FMT intervention markedly reduced ROS levels in the FMQ group. The Transwell assay results of HUVECs further demonstrated that the FM group displayed a significantly lower number of migrated cells than the FS group did, whereas this migratory capacity was substantially enhanced following FMQ intervention (**Figures 12F, G**). Additionally, we investigated the neovascularization mediated by FMQ in HUVECs. Compared with those in the FM group, tube formation in FMQ-treated HUVECs was significantly accelerated, manifested as an increase in the number of branches connecting cells and the formation of more tubular loops (**Figures 12H, I**). Furthermore, the scratch assay results indicated that, compared with that in the FS group, the migration rate of HUVECs in the FM group was significantly lower at 12 h and 24 h. However, treatment with FMQ markedly increased the migration rate at both time points (**Figures 12J, K**).

### 3.4 Que accelerated diabetic wound healing through the intestinal flora

**Figure 13:**
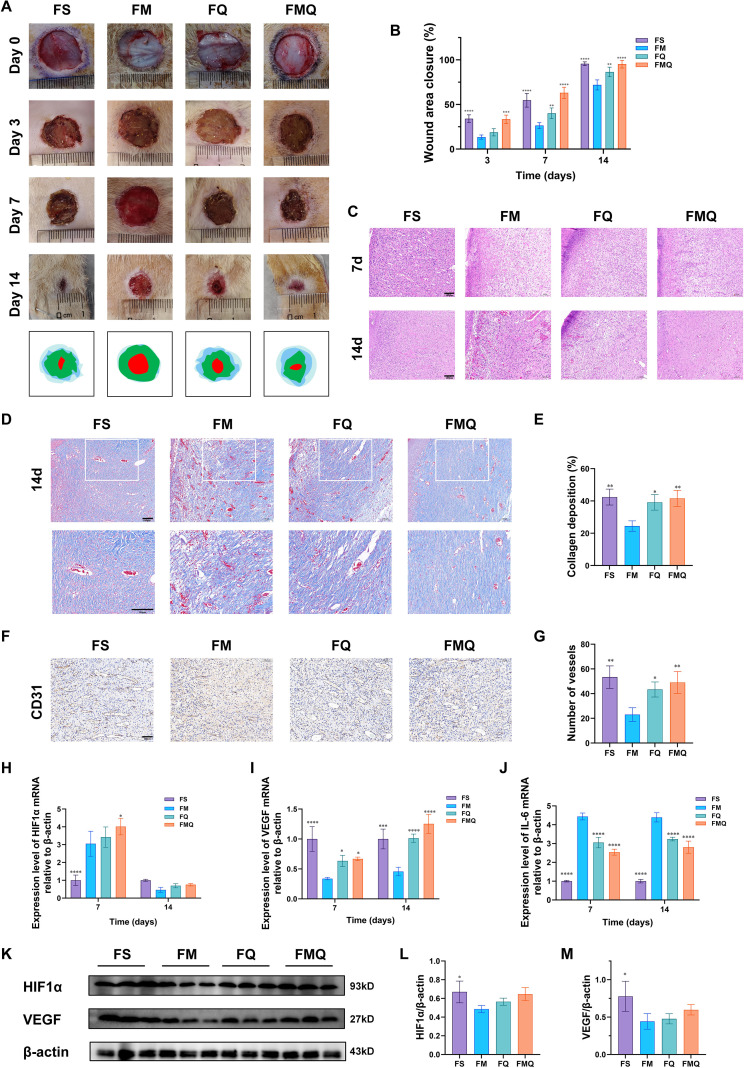
Effects of FMT on wound healing in diabetic rats. **(A)** Representative images depicting full-thickness skin defects in diabetic rats at various time points (0, 3, 7 and 14 days) following drug administration. **(B)** Wound healing rates in each group of diabetic rats. (All data are expressed as the mean ± SD (n = 6), *P < 0.05, **P < 0.01, ***P < 0.001, ****P < 0.0001, vs. FM) **(C)** Hematoxylin–eosin-stained images of the wounds of diabetic rats in each group on the 7th and 14th days after surgery. **(D)** Masson-stained images of the wounds of diabetic rats in each group on the 14th day after surgery. **(E)** Quantitative analysis on collagen deposition in each group. **(F)** Immunohistochemical detection of CD31 expression in the wounds of diabetic rats in each group on day 7. Positive CD31 staining (brown) revealed newly formed blood vessels. **(G)** Measurement of microvessel density in each group of rats on day 7. **(H)** The expression level of HIF1α mRNA in each group of diabetic wound tissues. **(I)** The expression level of VEGF mRNA in each group of diabetic wound tissues. **(J)** The expression level of IL-6 mRNA in each group of diabetic wound tissues. **(K)** The expression levels of HIF1α and VEGF in each group of diabetic wound tissues. **(L)** Statistical analysis of HIF1α levels in each group of diabetic wound tissues. **(M)** Statistical analysis of VEGF levels in each group of diabetic wound tissues. (All data are expressed as the mean ± SD (n = 3), *P < 0.05, **P < 0.01, ***P < 0.001, ****P < 0.0001, vs. FM) .

As shown in **Figures 13A, B**, both the FQ and FMQ groups exhibited improved wound healing rates within 14 days after intervention, with the FMQ group demonstrating superior efficacy. On day 7, diabetic wounds treated with FMQ presented an increased relative number of newly formed blood vessels, occasional neovascularization, and a collagen fiber structure coordinated with fibroblasts. By day 14, the FMQ group displayed more complete epidermal structural repair and more orderly cell arrangement, indicating robust tissue regeneration, increased matrix density, and enhanced structural integrity (**Figure 13C**). Furthermore, Masson’s trichrome staining results revealed that the wounds in the FMQ group were filled with abundant blue-stained collagen, demonstrating that this group significantly promoted collagen deposition during the wound healing process (**Figures 13D, E**). Immunohistochemical analysis revealed that CD31 expression was significantly reduced in FM, whereas treatment with FMQ markedly increased CD31 expression (**Figures 13F, G**). RT–qPCR analysis of wound tissues from the rats in each group revealed that FMQ significantly upregulated the mRNA expression levels of HIF1α and VEGF but downregulated the mRNA expression level of IL-6 (**Figures 13H-J**). Western blotting analysis further demonstrated that the expression levels of HIF1α and VEGF in wound tissues of rats in the FMQ group were significantly higher than those in the FM group (**Figures 13K-M**).

## 4 Discussion

The healing process of DFU is extremely complex and primarily involves insufficient neovascularization, neuropathy, a high risk of infection, tissue hypoxia, and nonphysiological inflammatory responses, which lead to delayed ulcer healing and the occurrence of highly proinflammatory chronic wounds (32, 33). TCM, characterized by low toxicity and comprehensive multitarget regulation, has developed rapidly in recent years for the treatment of chronic noncommunicable diseases (34). Research has indicated that certain types of flavonoids exhibit antidiabetic properties in animal studies (35). Additionally, studies have confirmed that flavonoids exhibit pleiotropic effects in the treatment of DFU (36). However, the precise mechanisms by which these flavonoids enhance glycemic control and treat diabetic complications remain unclear (37). Que, a potent bioactive flavonoid and a key constituent of *Evodia rutaecarpa* (38), exhibits anti-inflammatory (39) and antidiabetic effects (40). Despite its recognized benefits in diabetes management, the specific impact of Que on diabetic wound healing remains underexplored, which constitutes the primary focus of this study. Notably, molecular docking revealed stable conformations of Que bound to 9 key targets, indicating that it may be the core active component of *Evodia rutaecarpa* for anti-DFU activity.

The selection of a single dose of Que (25 mg/kg/day) is based on previously published studies (23) that have demonstrated that this dose can sustainably produce stable antioxidant and anti-inflammatory activity in relevant animal models. Further *in vivo* experiments revealed that Que significantly ameliorated glucose and lipid metabolism disorders and systemic inflammatory responses in diabetic wound-affected rats. Metabolomic analysis revealed that Que significantly reversed the dysregulation of 673 metabolites in the serum of DFU rats while concurrently ameliorating insulin resistance. Notably, gut microbiota analysis demonstrated that DFU induced the proliferation of proinflammatory bacteria such as *Desulfovibrionia* and *uncultured_rumen_bacterium* (41, 42), whereas Que specifically increased the abundance of anti-inflammatory bacteria, including *Ligilactobacillus* (43). The regulatory network constructed through integrated microbiota-metabolite analysis further confirmed that skin injury exacerbates systemic inflammation via negative regulation of the gut bacterium *Anaerosporobacter*. It is acknowledged that 16S rRNA sequencing limits the taxonomic resolution to the genus level compared to shotgun metagenomics. However, we integrated functional metabolomics to cross-validate the microbiome findings. The significant correlations observed between the differential gut taxa and specific circulating metabolites provide direct functional evidence supporting the “gut-skin axis” hypothesis, partially mitigating the limitations of amplicon-based sequencing. These findings indicate that Que may improve the local skin microenvironment by remodelling the homeostasis of the gut microbiota and metabolism and thereby inhibiting the systemic inflammatory cascade.

The intestinal barrier is a crucial component of the body’s barrier system and plays a defensive role in maintaining microecological homeostasis. Occludin, claudins, and ZO proteins are major molecular constituents of intestinal tight junctions. The primary functions of these tight junction proteins include regulating paracellular permeability and cell polarity, selectively permitting the passage of ions and small soluble molecules, and restricting the translocation of large molecules and foreign pathogenic microorganisms (44). Studies have shown that diabetic patients exhibit impaired intestinal barrier function and increased intestinal permeability (45), which are closely linked to dysbiosis of the gut microbiota (46). It has been demonstrated that cocoa supplementation can upregulate ZO-1 levels and reduce the expression of proinflammatory cytokines in the colon of diabetic rats (47).

In the process of diabetic wound healing, the regulation of inflammation and the expression of growth factors are critical determinants. Among these, the overexpression of CLEC14A can reverse high glucose-induced impairments in angiogenic capacity and elevated inflammatory levels, thereby accelerating wound healing (48). As a natural compound, Que has significant efficacy in alleviating diabetic metabolic disorders. It reduces blood glucose levels and improves insulin sensitivity by modulating multiple factors and signalling pathways implicated in insulin resistance and the pathogenesis of type 2 diabetes, such as TNF-α, NF-κB, AMPK, AKT, and NRF2 (49). It also mitigates the persistence of inflammatory cells and enhances wound tissue quality. Furthermore, it accelerates wound closure by promoting fibroblast proliferation, collagen synthesis, and re-epithelialization (50). Additionally, Que facilitates wound healing through enhanced angiogenesis and collagen deposition (51). However, its clinical application is limited by low bioavailability, which has prompted ongoing research into combination strategies with nanotechnology to improve delivery and efficacy (52). It has been shown that FMT promotes diabetic wound healing by modulating the gut microbiota, enhancing IL-17A production to facilitate keratinocyte proliferation and migration—a process mediated through the IL-17A–mTOR–HIF1α signalling axis (53). Our findings indicate that serum derived from FM group rats inhibits the viability of both RAW 264.7 cells and HUVECs, underscoring the influence of intestinal microbiota on the inflammatory wound environment. Moreover, the FMQ group rats displayed enhanced collagen deposition, increased neovascularization, and reduced inflammatory responses in wound tissues, further demonstrating that Que facilitates diabetic wound healing via regulation of the gut microbiota.

## 5 Conclusion

This study demonstrated that Que, the core active ingredient of *Evodia rutaecarpa*, significantly promotes diabetic wound healing by modulating inflammatory responses and enhancing angiogenesis through key targets identified via bioinformatics analysis. Furthermore, Que restored gut microbiota homeostasis and regulated serum metabolic profiles, confirming its role in ameliorating diabetic wounds via the “gut microbiota–inflammation–skin axis”. FMT and drug-containing serum experiments confirmed that Que-mediated gut microbiota remodelling contributes to reduced systemic inflammation, improved endothelial function, and accelerated wound repair. Future research will focus on identifying the specific microbial metabolites regulated by Que and elucidating their precise mechanisms in influencing downstream signaling communication between immune and reparative cells. Our findings further indicated that Chinese medicine represents a valuable resource for investigating this emerging therapeutic axis. This underscores the translational potential of targeting the “gut–skin axis” in diabetic wound management and offers new mechanistic perspectives on the treatment of DFU.

## Data Availability

The original contributions presented in the study are publicly available. This data can be found here: https://figshare.com/s/2f4e2715a2ab00c48104.

## Glossary

CD31: cluster of differentiation 31
DCFH-DA: 2’,7’-dichlorodihydrofluorescein diacetate
DFU: diabetic foot ulcer
DMSO: dimethyl sulfoxide
FBS: fetal bovine serum
FC: fold change
FM: faux aseptic model
FMQ: faux aseptic fecal microbiota transplantation quercetin
FMT: fecal microbiota transplantation
FQ: faux aseptic quercetin
FS: faux aseptic sham
GSEA: gene set enrichment analysis
HIF1α: hypoxia inducible factor-1 α
HUVECs: human umbilical vein endothelial cells
IL-1β: interleukin-1β
IL-6: interleukin-6
IL-10: interleukin-10
INS: insulin
KEGG: Kyoto Encyclopedia of Genes and Genomes
LDL-C: low-density lipoprotein cholesterol
LC–MS: liquid chromatography–mass spectrometry
MTT: 3-(4,5)-dimethylthiahiazo(-z-y1)-2,5-diphenytetrazoliumromide
OPLS-DA: orthogonal partial least squares-discriminant analysis
PBS: phosphate buffer solution
PCA: principal component analysis
Que: quercetin
RT–qPCR: real-time quantitative polymerase chain reaction
T-CHO: total cholesterol
TCM: traditional Chinese medicine
TG: triglyceride
TNF-α: tumor necrosis factor α
VEGF: vascular endothelial growth factor
VIP: variable importance in projection

## References

[1] Cornelis MC Hu FB . Gene-environment interactions in the development of type 2 diabetes: recent progress and continuing challenges. Annu Rev Nutr. (2012) 32:245–59. doi: 10.1146/annurev-nutr-071811-150648, PMID: 22540253

[2] Alexander M Cho E Gliozheni E Salem Y Cheung J Ichii H . Pathology of diabetes-induced immune dysfunction. Int J Mol Sci. (2024) 25(13):7105. doi: 10.3390/ijms25137105, PMID: 39000211 PMC11241249

[3] Aldana PC Cartron AM Khachemoune A . Reappraising diabetic foot ulcers: A focus on mechanisms of ulceration and clinical evaluation. Int J Low Extrem Wounds. (2022) 21:294–302. doi: 10.1177/1534734620944514, PMID: 32734837

[4] Hartmann B Fottner C Herrmann K Limbourg T Weber MM Beckh K . Interdisciplinary treatment of diabetic foot wounds in the elderly: Low risk of amputations and mortality and good chance of being mobile with good quality of life. Diabetes Vasc Dis Res. (2017) 14:55–8. doi: 10.1177/1479164116666477, PMID: 27941057

[5] Needell JC Zipris D . The role of the intestinal microbiome in type 1 diabetes pathogenesis. Curr Diabetes Rep. (2016) 16:89. doi: 10.1007/s11892-016-0781-z, PMID: 27523648

[6] Hara N Alkanani AK Ir D Robertson CE Wagner BD Frank DN . The role of the intestinal microbiota in type 1 diabetes. Clin Immunol. (2013) 146:112–9. doi: 10.1016/j.clim.2012.12.001, PMID: 23314185

[7] Jimenez-Sanchez M Celiberto LS Yang H Sham HP Vallance BA . The gut-skin axis: a bi-directional, microbiota-driven relationship with therapeutic potential. Gut Microbes. (2025) 17:2473524. doi: 10.1080/19490976.2025.2473524, PMID: 40050613 PMC11901370

[8] Chen M Wang R Wang T . Gut microbiota and skin pathologies: Mechanism of the gut-skin axis in atopic dermatitis and psoriasis. Int Immunopharmacol. (2024) 141:112658. doi: 10.1016/j.intimp.2024.112658, PMID: 39137625

[9] Dokoshi T Chen Y Cavagnero KJ Rahman G Hakim D Brinton S . Dermal injury drives a skin to gut axis that disrupts the intestinal microbiome and intestinal immune homeostasis in mice. Nat Commun. (2024) 15:3009. doi: 10.1038/s41467-024-47072-3, PMID: 38589392 PMC11001995

[10] Jiang P Di Z Huang W Xie L . Modulating the gut microbiota and metabolites with traditional Chinese medicines: an emerging therapy for type 2 diabetes mellitus and its complications. Molecules. (2024) 29. doi: 10.3390/molecules29122747, PMID: 38930814 PMC11206945

[11] Liao JF Chiou WF Shen YC Wang GJ Chen CF . Anti-inflammatory and anti-infectious effects of Evodia rutaecarpa (Wuzhuyu) and its major bioactive components. Chin Med. (2011) 6:6. doi: 10.1186/1749-8546-6-6, PMID: 21320305 PMC3046897

[12] Luo ZY Hu YX Qiu CW Chen WC Li L Chen FL . Coptidis Rhizoma processed with Evodia Rutaecarpa improves the effect on ulcerative colitis by increasing intestinal energy metabolites alpha-ketoglutarate and Lactobacillus reuteri. Phytomedicine. (2023) 121:155115. doi: 10.1016/j.phymed.2023.155115, PMID: 37801896

[13] Matsuda H Yoshikawa M Ido Y Amemiya T Kubo M . Augmenting Effect of Evodiae Fructus on Antiinflammatory and Antinociceptive Activities of Indomethacin Ointment. Natural Medicines. (2001) 55(5):235–42. https://mol.medicalonline.jp/en/archive/search?jo=cg5yakso&vo=55&issue=5

[14] Ru J Li P Wang J Zhou W Li B Huang C . TCMSP: a database of systems pharmacology for drug discovery from herbal medicines. J Cheminform. (2014) 6:13. doi: 10.1186/1758-2946-6-13, PMID: 24735618 PMC4001360

[15] Feramisco JD Sadreyev RI Murray ML Grishin NV Tsao H . Phenotypic and genotypic analyses of genetic skin disease through the Online Mendelian Inheritance in Man (OMIM) database. J Invest Dermatol. (2009) 129:2628–36. doi: 10.1038/jid.2009.108, PMID: 19536140

[16] Lin Y Hu Z . Bioinformatics analysis of candidate genes involved in ethanol-induced microtia pathogenesis based on a human genome database: GeneCards. Int J Pediatr Otorhinolaryngol. (2021) 142:110595. doi: 10.1016/j.ijporl.2020.110595, PMID: 33418206

[17] Shi C Ou X Huang L Lei X Xu S Ou M . Integrating GEO database, mendelian randomization, and molecular docking to identify HLA-C as a potential therapeutic target for periodontitis. Mediators Inflamm. (2025) 2025:9163431. doi: 10.1155/mi/9163431, PMID: 40692979 PMC12279435

[18] Jiang K Mai S Li J Zhou H Chen Y Zou L . Exploring the molecular mechanisms of lactylation-related biological functions and immune regulation in sepsis-associated acute kidney injury. Clin Exp Med. (2025) 25:200. doi: 10.1007/s10238-025-01745-5, PMID: 40504273 PMC12162736

[19] Mo Q Mo Q Mo F . Single-cell RNA sequencing and transcriptomic analysis reveal key genes and regulatory mechanisms in sepsis. Biotechnol Genet Eng Rev. (2024) 40:1636–58. doi: 10.1080/02648725.2023.2196475, PMID: 37017187

[20] Li K Wu L Jiang J . Apigenin accelerates wound healing in diabetic mice by promoting macrophage M2-type polarization via increasing miR-21 expression. Mol Cell Biochem. (2024) 479:3119–27. doi: 10.1007/s11010-023-04885-y, PMID: 38261238

[21] Huang Z Liu J Li H Ai Y Zhou D . Network pharmacology-based prediction and “gut microbiota-inflammation-brain axis” validation of the active ingredients and potential mechanisms of Plantagins Herba for treating diabetes-related cognitive dysfunction. Front Pharmacol. (2025) 16:1601689. doi: 10.3389/fphar.2025.1601689, PMID: 40626305 PMC12231498

[22] Jung JS Jo HY Hwang J Kim D Kwon M Yong J . Efficacy of subconjunctivally applied everolimus- and sirolimus-pretreated MSCs in preventing diabetic retinopathy. Transl Vis Sci Technol. (2025) 14:19. doi: 10.1167/tvst.14.9.19, PMID: 40952054 PMC12442941

[23] Maciel RM Costa MM Martins DB França RT Schmatz R Graça DL . Antioxidant and anti-inflammatory effects of quercetin in functional and morphological alterations in streptozotocin-induced diabetic rats. Res Vet Sci. (2013) 95:389–97. doi: 10.1016/j.rvsc.2013.04.028, PMID: 23706762

[24] Zhang P Wei W Zhang X Wen C Ovatlarnporn C Olatunji OJ . Antidiabetic and antioxidant activities of Mitragyna speciosa (kratom) leaf extract in type 2 diabetic rats. BioMed Pharmacother. (2023) 162:114689. doi: 10.1016/j.biopha.2023.114689, PMID: 37058820

[25] Siqueira BS Díaz Urrutia MA Ceglarek VM Moreira DC Brasil Kuzniewski FT Roberto de Souza de Almeida Leite J . A novel bombesin-related peptide modulates glucose tolerance and insulin secretion in non-obese and hypothalamic-obese rats. Toxicon. (2025) 255:108230. doi: 10.1016/j.toxicon.2025.108230, PMID: 39788326

[26] Pang J Maienschein-Cline M Koh TJ . Enhanced proliferation of Ly6C(+) monocytes/macrophages contributes to chronic inflammation in skin wounds of diabetic mice. J Immunol. (2021) 206:621–30. doi: 10.4049/jimmunol.2000935, PMID: 33443065 PMC7927918

[27] Chen Q Mushtaq W Wang X Liao Z Li J Xiao S . Crop rotation alleviates continuous cropping obstacles in Chrysanthemum morifolium production by regulating rhizosphere soil microbial communities and metabolites. Environ Microbiome. (2025) 20:90. doi: 10.1186/s40793-025-00754-x, PMID: 40676696 PMC12273239

[28] Chen M Wang Z He H He W Zhang Z Sun S . Multi-Omics Analysis Reveals the Regulatory Mechanism of Different Probiotics on Growth Performance and Intestinal Health of Salmo trutta (S. trutta). Microorganisms. (2024) 12(7):1410. doi: 10.3390/microorganisms12071410, PMID: 39065178 PMC11278557

[29] Chen R Xu Y Wu P Zhou H Lasanajak Y Fang Y . Transplantation of fecal microbiota rich in short chain fatty acids and butyric acid treat cerebral ischemic stroke by regulating gut microbiota. Pharmacol Res. (2019) 148:104403. doi: 10.1016/j.phrs.2019.104403, PMID: 31425750

[30] Liu J Liu J Tong X Peng W Wei S Sun T . Network pharmacology prediction and molecular docking-based strategy to discover the potential pharmacological mechanism of Huai Hua San against ulcerative colitis. Drug Des Devel Ther. (2021) 15:3255–76. doi: 10.2147/dddt.S319786, PMID: 34349502 PMC8326529

[31] Clayton SM Shafikhani SH Soulika AM . Macrophage and neutrophil dysfunction in diabetic wounds. Adv Wound Care (New Rochelle). (2024) 13:463–84. doi: 10.1089/wound.2023.0149, PMID: 38695109 PMC11535468

[32] Huang F Lu X Yang Y Yang Y Li Y Kuai L . Microenvironment-based diabetic foot ulcer nanomedicine. Adv Sci (Weinh). (2023) 10:e2203308. doi: 10.1002/advs.202203308, PMID: 36424137 PMC9839871

[33] Xiao S Zhang D Liu Z Jin W Huang G Wei Z . Diabetes-induced glucolipotoxicity impairs wound healing ability of adipose-derived stem cells-through the miR-1248/CITED2/HIF-1α pathway. Aging (Albany NY). (2020) 12:6947–65. doi: 10.18632/aging.103053, PMID: 32294623 PMC7202540

[34] Liu FS Li Y Guo XS Liu RC Zhang HY Li Z . Advances in traditional Chinese medicine as adjuvant therapy for diabetic foot. World J Diabetes. (2022) 13:851–60. doi: 10.4239/wjd.v13.i10.851, PMID: 36312004 PMC9606791

[35] Tong XL Dong L Chen L Zhen Z . Treatment of diabetes using traditional Chinese medicine: past, present and future. Am J Chin Med. (2012) 40:877–86. doi: 10.1142/s0192415x12500656, PMID: 22928822

[36] Miraj SS Kurian SJ Rodrigues GS Saravu K Rao M Raychaudhuri SP . Phytotherapy in diabetic foot ulcers: A promising strategy for effective wound healing. J Am Nutr Assoc. (2023) 42:295–310. doi: 10.1080/07315724.2022.2034069, PMID: 35512780

[37] Xu N Zhang L Dong J Zhang X Chen YG Bao B . Low-dose diet supplement of a natural flavonoid, luteolin, ameliorates diet-induced obesity and insulin resistance in mice. Mol Nutr Food Res. (2014) 58:1258–68. doi: 10.1002/mnfr.201300830, PMID: 24668788

[38] Dabeek WM Marra MV . Dietary quercetin and kaempferol: bioavailability and potential cardiovascular-related bioactivity in humans. Nutrients. (2019) 11(10):2288. doi: 10.3390/nu11102288, PMID: 31557798 PMC6835347

[39] Xie R Xu T Yin Y Liu M Huang C Zhang W . Quercetin attenuates DEHP-induced pyroptosis and programmed necrosis in chicken duodenum through regulation of the TLR4/MyD88/NF-κB pathway. Environ pollut. (2025) 372:126016. doi: 10.1016/j.envpol.2025.126016, PMID: 40057166

[40] Farhadi F Sharififar F Jafari M Rahimi VB Askari N Askari VR . Hallmarks of quercetin benefits as a functional supplementary in the management of diabetes mellitus-related maladies: from basic to clinical applications. Curr Drug Metab. (2025) 25(9):653–69. doi: 10.2174/0113892002339410250108031621, PMID: 39878112

[41] Cunha MI Su M Cantuti-Castelvetri L Müller SA Schifferer M Djannatian M . Pro-inflammatory activation following demyelination is required for myelin clearance and oligodendrogenesis. J Exp Med. (2020) 217(5):e20191390. doi: 10.1084/jem.20191390, PMID: 32078678 PMC7201919

[42] Zhong Y Xue MY Sun HZ Valencak TG Guan LL Liu J . Rumen and hindgut bacteria are potential indicators for mastitis of mid-lactating Holstein dairy cows. Microorganisms. (2020) 8(12):2042. doi: 10.3390/microorganisms8122042, PMID: 33419337 PMC7767203

[43] Rieu A Aoudia N Jego G Chluba J Yousfi N Briandet R . The biofilm mode of life boosts the anti-inflammatory properties of Lactobacillus. Cell Microbiol. (2014) 16:1836–53. doi: 10.1111/cmi.12331, PMID: 25052472

[44] Stewart AS Pratt-Phillips S Gonzalez LM . Alterations in intestinal permeability: the role of the “Leaky gut” in health and disease. J Equine Vet Sci. (2017) 52:10–22. doi: 10.1016/j.jevs.2017.02.009, PMID: 31000910 PMC6467570

[45] Zhao L Lou H Peng Y Chen S Fan L Li X . Elevated levels of circulating short-chain fatty acids and bile acids in type 2 diabetes are linked to gut barrier disruption and disordered gut microbiota. Diabetes Res Clin Pract. (2020) 169:108418. doi: 10.1016/j.diabres.2020.108418, PMID: 32891692

[46] Zhou W Yang T Xu W Huang Y Ran L Yan Y . The polysaccharides from the fruits of Lycium barbarum L. confer anti-diabetic effect by regulating gut microbiota and intestinal barrier. Carbohydr Polym. (2022) 291:119626. doi: 10.1016/j.carbpol.2022.119626, PMID: 35698418

[47] Álvarez-Cilleros D Ramos S López-Oliva ME Escrivá F Álvarez C Fernández-Millán E . Cocoa diet modulates gut microbiota composition and improves intestinal health in Zucker diabetic rats. Food Res Int. (2020) 132:109058. doi: 10.1016/j.foodres.2020.109058, PMID: 32331673

[48] Liao Y Wu N Guo L Yang D . CLEC14A facilitates angiogenesis and alleviates inflammation in diabetic wound healing. Life Sci. (2024) 358:123176. doi: 10.1016/j.lfs.2024.123176, PMID: 39454994

[49] Yan L Vaghari-Tabari M Malakoti F Moein S Qujeq D Yousefi B . Quercetin: an effective polyphenol in alleviating diabetes and diabetic complications. Crit Rev Food Sci Nutr. (2023) 63:9163–86. doi: 10.1080/10408398.2022.2067825, PMID: 35468007

[50] Kant V Jangir BL Sharma M Kumar V Joshi VG . Topical application of quercetin improves wound repair and regeneration in diabetic rats. Immunopharmacol Immunotoxicol. (2021) 43:536–53. doi: 10.1080/08923973.2021.1950758, PMID: 34278923

[51] Wang C Chen H Wang W Yan G Zheng S Wang C . Facile strategy for gelatin-based hydrogel with multifunctionalities to remodel wound microenvironment and accelerate healing of acute and diabetic wounds. Int J Biol Macromol. (2024) 256:128372. doi: 10.1016/j.ijbiomac.2023.128372, PMID: 38000588

[52] Panthi VK Imran M Chaudhary A Paudel KR Mohammed Y . The significance of quercetin-loaded advanced nanoformulations for the management of diabetic wounds. Nanomedicine (Lond). (2023) 18:391–411. doi: 10.2217/nnm-2022-0281, PMID: 37140389

[53] Peng C Lei P Qi H Zhu Q Huang C Fu J . Effect of fecal microbiota transplantation on diabetic wound healing through the IL-17A-mTOR-HIF1α signaling axis. Appl Environ Microbiol. (2025) 91:e0201924. doi: 10.1128/aem.02019-24, PMID: 40019272 PMC11921319

