# Supplementary material for: Integrating bioinformatic prediction and the “gut microbiota-inflammation-skin axis” to decipher the mechanisms of quercetin (from Evodia rutaecarpa) in diabetic wound healing
Source: Front Immunol. 2026 Feb 24;17:1755280. doi: 10.3389/fimmu.2026.1755280 (PMC12974265; doi:10.3389/fimmu.2026.1755280)
Supplement: Supplementary file 1 [file Table1.docx]

Supplementary Material

# Supplementary Table

**Table S1 Differences metabolites of quercetin intervention diabetic wound rat model**

| No. | Ionic mode | name | Fold_change | | P value | | VIP | | Regulated | |
| --- | --- | --- | --- | --- | --- | --- | --- | --- | --- | --- |
|  |  |  | AvsB | BvsC | AvsB | BvsC | AvsB | BvsC | AvsB | BvsC |
| 1 | neg_1110 | 9-(1,3-Dioxolan-2-yl)purine-2,6-diamine | 2.4732 | 0.5362 | 0.0000 | 0.0000 | 1.1497 | 1.2377 | up | down |
| 2 | neg_1111 | 2,5-Dioxopentanoate | 3.2149 | 0.5165 | 0.0000 | 0.0000 | 1.1488 | 1.2360 | up | down |
| 3 | neg_1113 | Norophthalmic acid | 0.3502 | 1.3277 | 0.0000 | 0.0000 | 1.1447 | 1.2115 | down | up |
| 4 | neg_1166 | L-trans-5-Hydroxy-2-piperidinecarboxylic acid | 2.3419 | 0.5195 | 0.0000 | 0.0000 | 1.1491 | 1.2355 | up | down |
| 5 | neg_1168 | D-Glucuronic acid | 6.5559 | 0.3971 | 0.0000 | 0.0000 | 1.1487 | 1.2313 | up | down |
| 6 | neg_1171 | 7-Hydroxy-4-(trifluoromethyl)coumarin | 3.1493 | 0.4154 | 0.0000 | 0.0000 | 1.1470 | 1.2375 | up | down |
| 7 | neg_1189 | Raffinose | 11.6823 | 0.3865 | 0.0000 | 0.0000 | 1.1506 | 1.2407 | up | down |
| 8 | neg_1190 | Coriandrone E | 17.9464 | 0.3635 | 0.0000 | 0.0000 | 1.1494 | 1.2387 | up | down |
| 9 | neg_1209 | Isoniazid alpha-ketoglutaric acid | 0.3805 | 1.2914 | 0.0000 | 0.0000 | 1.1450 | 1.1294 | down | up |
| 10 | neg_1316 | Pyroglutamic acid | 0.4715 | 1.1796 | 0.0000 | 0.0003 | 1.1451 | 1.1289 | down | up |
| 11 | neg_1359 | 4-Hydroxy-L-phenylglycine | 0.3974 | 1.6491 | 0.0000 | 0.0000 | 1.1254 | 1.1981 | down | up |
| 12 | neg_1429 | L-Allysine Ethylene Acetal | 12.8643 | 0.7619 | 0.0000 | 0.0003 | 1.1455 | 1.1534 | up | down |
| 13 | neg_1432 | Isovalerylglutamic acid | 15.9661 | 0.7913 | 0.0000 | 0.0010 | 1.1432 | 1.0734 | up | down |
| 14 | neg_1433 | (+)-2-Amino-7-phosphonoheptanoic acid | 8.8764 | 0.8014 | 0.0000 | 0.0000 | 1.1495 | 1.1916 | up | down |
| 15 | neg_1448 | GMP | 8.4687 | 0.7804 | 0.0000 | 0.0006 | 1.1447 | 1.1400 | up | down |
| 16 | neg_1452 | 2'-Deoxy-5-hydroxymethylcytidine-5'-diphosphate | 7.4639 | 0.7953 | 0.0000 | 0.0001 | 1.1480 | 1.1799 | up | down |
| 17 | neg_1499 | Dihydrolipoate | 0.0430 | 4.4969 | 0.0000 | 0.0000 | 1.1431 | 1.1644 | down | up |
| 18 | neg_1501 | Fluoroazomycin arabinoside | 27.0500 | 0.8578 | 0.0000 | 0.0014 | 1.1483 | 1.0092 | up | down |
| 19 | neg_1505 | 1-Deoxy-1-morpholino-D-fructose | 15.8447 | 0.7503 | 0.0000 | 0.0000 | 1.1482 | 1.1959 | up | down |
| 20 | neg_1521 | 4-Dedimethylamino sancycline | 10.0808 | 0.8272 | 0.0000 | 0.0000 | 1.1493 | 1.1897 | up | down |
| 21 | neg_1544 | UDP-kanosamine | 5.9507 | 0.7538 | 0.0000 | 0.0000 | 1.1494 | 1.2247 | up | down |
| 22 | neg_1571 | 2-Acetamido-2,6-dideoxygalactose | 0.3965 | 3.4659 | 0.0000 | 0.0000 | 1.1286 | 1.2165 | down | up |
| 23 | neg_1817 | Asparaginylhistidine | 2.5136 | 0.7320 | 0.0000 | 0.0002 | 1.1323 | 1.1400 | up | down |
| 24 | neg_1875 | 2-Deoxy-scyllo-inosose | 2.6433 | 0.9229 | 0.0000 | 0.0006 | 1.1487 | 1.0421 | up | down |
| 25 | neg_1886 | 2-n-Propylthiazolidine-4-carboxylic acid | 2.4072 | 0.7190 | 0.0000 | 0.0000 | 1.1477 | 1.2328 | up | down |
| 26 | neg_1895 | O-Succinyl-L-homoserine | 0.3517 | 1.2514 | 0.0000 | 0.0000 | 1.1470 | 1.1344 | down | up |
| 27 | neg_1897 | (R)-2-(4-(Tert-butoxycarbonyl)morpholin-3-yl)acetic acid | 7.5231 | 0.7727 | 0.0000 | 0.0000 | 1.1509 | 1.2286 | up | down |
| 28 | neg_1922 | (E)-2-(3-(Naphthalen-2-yl)but-2-enamido)benzoic acid | 8.7126 | 0.7664 | 0.0000 | 0.0000 | 1.1508 | 1.2370 | up | down |
| 29 | neg_1982 | 15-Demethyl plumieride | 5.5492 | 0.8233 | 0.0000 | 0.0000 | 1.1481 | 1.1443 | up | down |
| 30 | neg_1985 | 3'-Phosphoadenylyl sulfate | 6.4781 | 0.7879 | 0.0000 | 0.0000 | 1.1507 | 1.2338 | up | down |
| 31 | neg_2037 | Citalopram propionic acid | 133.0300 | 0.6096 | 0.0000 | 0.0000 | 1.1466 | 1.1899 | up | down |
| 32 | neg_2281 | N-Methyl-1-deoxynojirimycin | 2.7744 | 0.7261 | 0.0000 | 0.0000 | 1.1471 | 1.1991 | up | down |
| 33 | neg_2284 | Xanthurenate-8-O-beta-D-glucoside | 17.3514 | 0.7272 | 0.0000 | 0.0000 | 1.1502 | 1.2277 | up | down |
| 34 | neg_2285 | N-Acetylneuraminate 9-phosphate | 1717311461.5076 | 0.7106 | 0.0000 | 0.0000 | 1.1487 | 1.1822 | up | down |
| 35 | neg_2319 | L-Isoleucine | 6.1552 | 0.7768 | 0.0000 | 0.0000 | 1.1501 | 1.2262 | up | down |
| 36 | neg_2321 | 1,1-Bis(2-aminoethyl)-2-hydroxy-3-oxotriazane | 2.8860 | 0.8202 | 0.0000 | 0.0000 | 1.1461 | 1.1940 | up | down |
| 37 | neg_2322 | methyl 1-methylpiperidine-3-carboxylate | 12.6324 | 0.7106 | 0.0000 | 0.0000 | 1.1503 | 1.2309 | up | down |
| 38 | neg_2323 | 5-(1-Pyrrolidinylmethyl)-2-furanmethanol | 804097316.2156 | 0.6779 | 0.0000 | 0.0000 | 1.1500 | 1.2118 | up | down |
| 39 | neg_2324 | L-Valine, N-(2-hydroxy-3-butenyl)- | 91.6023 | 0.6941 | 0.0000 | 0.0000 | 1.1497 | 1.2230 | up | down |
| 40 | neg_2328 | N-(1-Deoxy-1-fructosyl)isoleucine | 13.2102 | 0.7043 | 0.0000 | 0.0000 | 1.1505 | 1.2301 | up | down |
| 41 | neg_2336 | Dihydrozeatin-7-N-glucoside | 12.1187 | 0.8590 | 0.0000 | 0.0000 | 1.1504 | 1.2001 | up | down |
| 42 | neg_2343 | LysoPI 16-0 | 9528.3815 | 0.5516 | 0.0000 | 0.0000 | 1.1496 | 1.2326 | up | down |
| 43 | neg_2345 | Flumazenil | 1499379377.8172 | 0.4713 | 0.0000 | 0.0000 | 1.1503 | 1.2375 | up | down |
| 44 | neg_2349 | Balsalazide | 192.2810 | 0.6987 | 0.0000 | 0.0001 | 1.1439 | 1.1536 | up | down |
| 45 | neg_2355 | Acetyl-DL-Leucine | 5.7413 | 0.8535 | 0.0000 | 0.0005 | 1.1473 | 1.0514 | up | down |
| 46 | neg_2366 | N-lactoyl-phenylalanine | 3.1834 | 0.6342 | 0.0000 | 0.0000 | 1.1503 | 1.2398 | up | down |
| 47 | neg_2436 | Lactacystin | 3.4673 | 0.6154 | 0.0000 | 0.0000 | 1.1450 | 1.2152 | up | down |
| 48 | neg_2438 | 5-Fluorodeoxyuridine diphosphate | 6.3322 | 0.5421 | 0.0000 | 0.0000 | 1.1435 | 1.2078 | up | down |
| 49 | neg_2442 | N-Lactoylphenylalanine | 2.9423 | 0.6349 | 0.0000 | 0.0000 | 1.1485 | 1.2351 | up | down |
| 50 | neg_2443 | 6'-Oxolividamine | 3.0338 | 0.6376 | 0.0000 | 0.0000 | 1.1479 | 1.2320 | up | down |
| 51 | neg_2445 | Niazirin | 4.2485 | 0.6021 | 0.0000 | 0.0000 | 1.1463 | 1.2057 | up | down |
| 52 | neg_2446 | BEFLOXATONE | 3.2644 | 0.6357 | 0.0000 | 0.0000 | 1.1482 | 1.2277 | up | down |
| 53 | neg_2448 | 5-Sulfosalicylic acid | 2.4497 | 0.7379 | 0.0000 | 0.0000 | 1.1453 | 1.1869 | up | down |
| 54 | neg_2449 | pyramid | 0.4404 | 1.2554 | 0.0000 | 0.0000 | 1.1335 | 1.1397 | down | up |
| 55 | neg_2487 | Phosphoguanidinoacetate | 0.2165 | 4.3016 | 0.0000 | 0.0000 | 1.1433 | 1.2403 | down | up |
| 56 | neg_2641 | Sulfamethoxazole N1-glucuronide | 2.1370 | 0.7810 | 0.0000 | 0.0001 | 1.1242 | 1.1317 | up | down |
| 57 | neg_2646 | 5-Hydroxymethyldeoxycytidylate | 2.6874 | 0.6783 | 0.0000 | 0.0000 | 1.1470 | 1.2257 | up | down |
| 58 | neg_2652 | Lividomycin B | 2.4877 | 0.5674 | 0.0000 | 0.0000 | 1.1412 | 1.2133 | up | down |
| 59 | neg_2668 | WKYMVm | 3.2654 | 0.7936 | 0.0000 | 0.0000 | 1.1470 | 1.1563 | up | down |
| 60 | neg_2673 | Hydroquinone | 0.1101 | 18.8675 | 0.0000 | 0.0000 | 1.1480 | 1.2413 | down | up |
| 61 | neg_2675 | L-arogenate | 0.3273 | 1.4697 | 0.0000 | 0.0000 | 1.1456 | 1.1922 | down | up |
| 62 | neg_2676 | Glycylprolylhydroxyproline | 2.4140 | 0.6941 | 0.0000 | 0.0000 | 1.1452 | 1.2234 | up | down |
| 63 | neg_2677 | (S)-2-Amino-5-((4-nitrophenyl)amino)-5-oxopentanoic acid | 0.4005 | 1.3905 | 0.0000 | 0.0000 | 1.1310 | 1.2037 | down | up |
| 64 | neg_2678 | N5-Acetyl-N2-gamma-L-glutamyl-L-ornithine | 2.8169 | 0.6666 | 0.0000 | 0.0000 | 1.1446 | 1.2144 | up | down |
| 65 | neg_2682 | Nesbuvir | 0.2604 | 1.9146 | 0.0000 | 0.0000 | 1.1214 | 1.2026 | down | up |
| 66 | neg_2683 | 2-Ethoxy-3-[4-[2-[4-(trifluoromethylsulfonyloxy)phenyl]ethoxy]phenyl]propanoic acid | 0.3418 | 1.5931 | 0.0003 | 0.0000 | 1.0830 | 1.1717 | down | up |
| 67 | neg_2685 | dTDP-4-amino-2,3,4,6-tetradeoxy-D-glucose | 0.3133 | 2.0392 | 0.0001 | 0.0000 | 1.1031 | 1.2200 | down | up |
| 68 | neg_2697 | 3-O-Methylniveusin A | 0.2971 | 1.6221 | 0.0000 | 0.0000 | 1.1247 | 1.2292 | down | up |
| 69 | neg_2706 | UDP-3-O-(3-hydroxytetradecanoyl)-D-glucosamine | 0.4726 | 1.5658 | 0.0004 | 0.0014 | 1.0370 | 1.0288 | down | up |
| 70 | neg_2708 | N-Formylmethionine | 0.2942 | 1.7616 | 0.0000 | 0.0000 | 1.1487 | 1.2358 | down | up |
| 71 | neg_2727 | Resorufin benzyl ether | 2.0212 | 0.7683 | 0.0000 | 0.0000 | 1.1419 | 1.2090 | up | down |
| 72 | neg_2728 | Pantoprazole | 2.0045 | 0.7733 | 0.0000 | 0.0000 | 1.1479 | 1.2221 | up | down |
| 73 | neg_2729 | UDP-N-acetylmuraminate | 0.4037 | 1.4829 | 0.0003 | 0.0019 | 1.0689 | 1.0372 | down | up |
| 74 | neg_2732 | 2'',3''-Di-O-p-coumaroylafzelin | 5.4979 | 0.5412 | 0.0000 | 0.0000 | 1.1486 | 1.2349 | up | down |
| 75 | neg_2733 | Versiconal | 17.7383 | 0.4421 | 0.0000 | 0.0000 | 1.1459 | 1.2251 | up | down |
| 76 | neg_2743 | Cinchophen | 2.0387 | 0.7412 | 0.0000 | 0.0000 | 1.1484 | 1.2362 | up | down |
| 77 | neg_2749 | Adefovir | 2.0879 | 0.7374 | 0.0000 | 0.0000 | 1.1488 | 1.2350 | up | down |
| 78 | neg_2750 | Sulfamoxole | 2.0513 | 0.7417 | 0.0000 | 0.0000 | 1.1491 | 1.2316 | up | down |
| 79 | neg_2752 | alpha-Cephalin | 2.2204 | 0.7918 | 0.0000 | 0.0000 | 1.1451 | 1.2221 | up | down |
| 80 | neg_2757 | Cefadroxil | 2.0730 | 0.7304 | 0.0000 | 0.0000 | 1.1493 | 1.2339 | up | down |
| 81 | neg_2761 | Hydroxylansoprazole | 2.2688 | 0.7435 | 0.0000 | 0.0000 | 1.1479 | 1.2171 | up | down |
| 82 | neg_2762 | Cephalosporin C | 2.4309 | 0.7079 | 0.0000 | 0.0000 | 1.1469 | 1.1956 | up | down |
| 83 | neg_2768 | CTP | 2.0799 | 0.6936 | 0.0000 | 0.0000 | 1.1487 | 1.2339 | up | down |
| 84 | neg_2771 | 2',3'-Cyclic CMP | 0.4963 | 1.2943 | 0.0001 | 0.0001 | 1.1062 | 1.1475 | down | up |
| 85 | neg_2772 | 1,3,4-Trigalloyl-beta-D-glucopyranose | 0.4912 | 1.2940 | 0.0000 | 0.0000 | 1.1129 | 1.1680 | down | up |
| 86 | neg_2778 | Bacampicillin | 640.8623 | 0.7083 | 0.0000 | 0.0000 | 1.1473 | 1.1844 | up | down |
| 87 | neg_2779 | 3-Hydroxypimeloyl-CoA | 50.6261 | 0.4597 | 0.0000 | 0.0000 | 1.1460 | 1.2147 | up | down |
| 88 | neg_2880 | 4-Hydroxy-4-(3-pyridyl)-butanoic acid | 0.2640 | 1.7867 | 0.0000 | 0.0003 | 1.1363 | 1.0964 | down | up |
| 89 | neg_2888 | Acetyl-L-tyrosine | 0.2014 | 2.0274 | 0.0000 | 0.0000 | 1.1494 | 1.2366 | down | up |
| 90 | neg_2903 | Paracetamol sulfate | 0.4552 | 4.3813 | 0.0000 | 0.0000 | 1.1463 | 1.2407 | down | up |
| 91 | neg_2911 | 4-Hydroxybenzenesulfonic acid | 2.9408 | 0.4626 | 0.0000 | 0.0000 | 1.1509 | 1.2417 | up | down |
| 92 | neg_2912 | 4-Amino-5-aminomethyl-2-methylpyrimidine | 7.5609 | 0.3097 | 0.0000 | 0.0000 | 1.1507 | 1.2416 | up | down |
| 93 | neg_2927 | Diphenol glucuronide | 0.0390 | 115.3164 | 0.0000 | 0.0000 | 1.1480 | 1.2403 | down | up |
| 94 | neg_2935 | D-1-Aminopropan-2-ol O-phosphate | 2.6189 | 0.7787 | 0.0000 | 0.0000 | 1.1447 | 1.2090 | up | down |
| 95 | neg_2936 | Quebrachidine | 15.5995 | 0.4064 | 0.0000 | 0.0000 | 1.1492 | 1.2354 | up | down |
| 96 | neg_2948 | 6-Fluorohomovanillic acid | 3.5211 | 0.8494 | 0.0000 | 0.0014 | 1.1361 | 1.0062 | up | down |
| 97 | neg_2949 | Decitabine | 5.0410 | 0.5657 | 0.0000 | 0.0000 | 1.1422 | 1.2247 | up | down |
| 98 | neg_2954 | 4-Hydroxystachydrine | 3.3493 | 0.7297 | 0.0000 | 0.0000 | 1.1504 | 1.2335 | up | down |
| 99 | neg_2955 | 3-Hydroxyisovalerate | 0.2622 | 2.4976 | 0.0000 | 0.0000 | 1.1470 | 1.2337 | down | up |
| 100 | neg_2963 | 2-Methoxyestrone 3-glucuronide | 24.5548 | 0.3100 | 0.0000 | 0.0000 | 1.1472 | 1.2332 | up | down |
| 101 | neg_2966 | Quinoline-4,8-diol | 2.8982 | 0.8775 | 0.0000 | 0.0001 | 1.1478 | 1.1083 | up | down |
| 102 | neg_2968 | Elenolide | 3.8019 | 0.5879 | 0.0000 | 0.0000 | 1.1506 | 1.2395 | up | down |
| 103 | neg_2972 | 4-Amino-1-[(2R,3S,4S,5R)-3,4-dihydroxy-5-(hydroxymethyl)oxolan-2-yl]oxypyrimidin-2-one | 72.3721 | 0.2747 | 0.0000 | 0.0000 | 1.1489 | 1.2354 | up | down |
| 104 | neg_2973 | Biocytin | 4.2232 | 0.5051 | 0.0000 | 0.0000 | 1.1501 | 1.2392 | up | down |
| 105 | neg_2978 | 2,8-Dihydroxyquinoline-beta-D-glucuronide | 0.4790 | 4.0742 | 0.0000 | 0.0000 | 1.1463 | 1.2410 | down | up |
| 106 | neg_2983 | Indoxylsulfuric acid | 2.2035 | 0.5076 | 0.0000 | 0.0000 | 1.1503 | 1.2412 | up | down |
| 107 | neg_2990 | Isoindolin-1-one | 2.5546 | 0.4791 | 0.0000 | 0.0000 | 1.1456 | 1.2357 | up | down |
| 108 | neg_2996 | 2,8-Quinolinediol 2-sulfate | 0.2635 | 3.4495 | 0.0000 | 0.0000 | 1.1501 | 1.2407 | down | up |
| 109 | neg_2999 | (2S,2'S)-Pyrosaccharopine | 39.9635 | 0.7030 | 0.0000 | 0.0000 | 1.1452 | 1.1774 | up | down |
| 110 | neg_3006 | 4-Vinylphenol sulfate | 0.1835 | 6.9237 | 0.0000 | 0.0000 | 1.1496 | 1.2413 | down | up |
| 111 | neg_3021 | Pyroglutamyl-glutamyl-proline amide | 2.5646 | 0.7593 | 0.0000 | 0.0002 | 1.1360 | 1.1568 | up | down |
| 112 | neg_3023 | 2-Hydroxypropyl methacrylamide | 6.4130 | 0.8479 | 0.0000 | 0.0000 | 1.1495 | 1.1889 | up | down |
| 113 | neg_3048 | p-Acetaminobenzoic acid | 0.3351 | 5.6485 | 0.0000 | 0.0000 | 1.1497 | 1.2418 | down | up |
| 114 | neg_3072 | 6-Hydroxy-5-methoxyindole glucuronide | 0.2679 | 1.5389 | 0.0000 | 0.0000 | 1.1501 | 1.2355 | down | up |
| 115 | neg_3074 | Ascorbic acid | 0.4699 | 1.4583 | 0.0000 | 0.0000 | 1.1448 | 1.2128 | down | up |
| 116 | neg_3076 | Trp derivative | 0.0115 | 3.8931 | 0.0000 | 0.0000 | 1.1498 | 1.1874 | down | up |
| 117 | neg_3094 | N2-Ethyl-2'-deoxyguanosine | 0.3413 | 12.1872 | 0.0000 | 0.0000 | 1.1199 | 1.2417 | down | up |
| 118 | neg_3141 | Malvidin 3-rhamnoside | 17.2197 | 0.5443 | 0.0000 | 0.0000 | 1.1502 | 1.2372 | up | down |
| 119 | neg_3148 | Panaquinquecol 4 | 2.3996 | 0.4572 | 0.0000 | 0.0000 | 1.1482 | 1.2378 | up | down |
| 120 | neg_3159 | Epothilone D | 7.2716 | 0.4000 | 0.0000 | 0.0000 | 1.1458 | 1.2321 | up | down |
| 121 | neg_3171 | methyl 2-[(2,3-dihydroxybenzoyl)amino]-3-hydroxypropanoate | 29.0872 | 0.6747 | 0.0000 | 0.0000 | 1.1475 | 1.2095 | up | down |
| 122 | neg_3172 | 5'-O-methylthymidine | 0.0205 | 6.6348 | 0.0000 | 0.0000 | 1.1498 | 1.1694 | down | up |
| 123 | neg_3391 | DG(LTE4/2:0/0:0) | 0.4912 | 2.2109 | 0.0000 | 0.0000 | 1.1174 | 1.2279 | down | up |
| 124 | neg_3434 | (3Z)-Phycoerythrobilin | 0.4723 | 2.9274 | 0.0000 | 0.0000 | 1.1443 | 1.2340 | down | up |
| 125 | neg_3439 | (2R)-2-[[(2R)-2-[[(2S)-2-(Azepane-1-carbonylamino)-4-methylpentanoyl]amino]-3-(1-formylindol-3-yl)propanoyl]amino]-3-(1H-indol-3-yl)propanoic acid | 0.3019 | 2.1673 | 0.0000 | 0.0000 | 1.1473 | 1.2239 | down | up |
| 126 | neg_3505 | Neopetasitenine | 6.3510 | 0.6753 | 0.0000 | 0.0000 | 1.1466 | 1.2132 | up | down |
| 127 | neg_3515 | Phenylacetylglycine | 6.9349 | 0.3897 | 0.0000 | 0.0000 | 1.1508 | 1.2416 | up | down |
| 128 | neg_3531 | sn-Glycero-3-phosphoethanolamine | 3.6936 | 0.5703 | 0.0000 | 0.0000 | 1.1480 | 1.2013 | up | down |
| 129 | neg_3610 | Fonsecin B | 0.3536 | 3.3569 | 0.0000 | 0.0000 | 1.1454 | 1.2333 | down | up |
| 130 | neg_3611 | Deoxyloganin | 0.0042 | 172.3484 | 0.0000 | 0.0000 | 1.1490 | 1.2397 | down | up |
| 131 | neg_3619 | Deoxycholic acid 3-glucuronide | 5.0800 | 0.9025 | 0.0000 | 0.0000 | 1.1502 | 1.1627 | up | down |
| 132 | neg_3624 | dITP | 5.8984 | 0.2874 | 0.0000 | 0.0000 | 1.1436 | 1.2322 | up | down |
| 133 | neg_3630 | 4,1-Benzoxazepine | 0.1929 | 2.0007 | 0.0000 | 0.0000 | 1.1501 | 1.2395 | down | up |
| 134 | neg_3631 | p-Tolyl Sulfate | 3.2654 | 0.4453 | 0.0000 | 0.0000 | 1.1490 | 1.2387 | up | down |
| 135 | neg_3635 | Heptylmalonic acid | 0.3359 | 2.3432 | 0.0000 | 0.0000 | 1.1425 | 1.2237 | down | up |
| 136 | neg_3650 | 4-(2-Furanylmethylene)-3,4-dihydro-2H-pyrrole | 0.1738 | 2.0140 | 0.0000 | 0.0001 | 1.1474 | 1.1604 | down | up |
| 137 | neg_3651 | Piliformic-acid | 0.1783 | 1.9522 | 0.0000 | 0.0000 | 1.1508 | 1.2349 | down | up |
| 138 | neg_3653 | Quercetin 3-O-acetyl-rhamnoside | 217.8744 | 0.6650 | 0.0000 | 0.0000 | 1.1457 | 1.1630 | up | down |
| 139 | neg_3682 | p-Benzosemiquinone | 6.5328 | 0.3028 | 0.0000 | 0.0000 | 1.1437 | 1.2283 | up | down |
| 140 | neg_3683 | Velaresol | 4.5027 | 0.3383 | 0.0000 | 0.0000 | 1.1508 | 1.2416 | up | down |
| 141 | neg_3686 | Hydroxytyrosol 3'-glucuronide | 2.8950 | 0.4124 | 0.0000 | 0.0000 | 1.1477 | 1.2356 | up | down |
| 142 | neg_3692 | cis-Mulberroside A | 659.6999 | 0.0861 | 0.0000 | 0.0000 | 1.1390 | 1.2261 | up | down |
| 143 | neg_3768 | 4-Oxo-1-(3-pyridyl)-1-butanone | 9.8166 | 0.2014 | 0.0000 | 0.0000 | 1.1482 | 1.2394 | up | down |
| 144 | neg_3779 | Blasticidin S | 1191.9296 | 0.2370 | 0.0000 | 0.0000 | 1.1496 | 1.2393 | up | down |
| 145 | neg_3805 | Glycosyl-4,4'-diaponeurosporenoate | 3.1599 | 0.7448 | 0.0000 | 0.0000 | 1.1487 | 1.2268 | up | down |
| 146 | neg_3814 | 2-Cyclopenten-1-one | 39.8163 | 0.3554 | 0.0000 | 0.0000 | 1.1463 | 1.2293 | up | down |
| 147 | neg_3838 | Lucidenic acid E2 | 0.1665 | 4.2066 | 0.0000 | 0.0000 | 1.1448 | 1.2400 | down | up |
| 148 | neg_3840 | BQ 123 | 0.3002 | 1.6879 | 0.0000 | 0.0000 | 1.1479 | 1.2295 | down | up |
| 149 | neg_3883 | Estrone glucuronide | 63.6401 | 0.6940 | 0.0000 | 0.0000 | 1.1502 | 1.2269 | up | down |
| 150 | neg_3890 | Salutaridine | 0.2774 | 3.5914 | 0.0000 | 0.0000 | 1.1414 | 1.2238 | down | up |
| 151 | neg_3959 | 6-Keto-prostaglandin E1 | 0.2208 | 11.0275 | 0.0000 | 0.0000 | 1.1445 | 1.2391 | down | up |
| 152 | neg_3989 | Nalidixic Acid | 0.2472 | 1.9778 | 0.0000 | 0.0000 | 1.1473 | 1.2405 | down | up |
| 153 | neg_4074 | 2-[(4S,5S,5aS,9aS)-4-methoxy-6,6,9a-trimethyl-5-[(2E,4E,6E)-octa-2,4,6-trienoyl]oxy-1-oxo-4,5,5a,7,8,9-hexahydro-3H-benzo[e]isoindol-2-yl]pentanedioic acid | 1793550236.8820 | 0.4007 | 0.0000 | 0.0000 | 1.1500 | 1.2381 | up | down |
| 154 | neg_4091 | Phosphocholine,1-Decanoyl-2-Hydroxy-sn-Glycero-3- | 688919738.3663 | 0.7833 | 0.0000 | 0.0000 | 1.1498 | 1.2017 | up | down |
| 155 | neg_4119 | E-10-Hydroxynortriptyline | 0.1861 | 3.7367 | 0.0000 | 0.0000 | 1.1489 | 1.2350 | down | up |
| 156 | neg_4132 | Taraxinic acid glucosyl ester | 3.7836 | 0.6799 | 0.0000 | 0.0000 | 1.1476 | 1.2244 | up | down |
| 157 | neg_4146 | PC(P-18:1(11Z)/PGE2) | 0.3270 | 2.5826 | 0.0000 | 0.0000 | 1.1477 | 1.2379 | down | up |
| 158 | neg_4167 | zaltoprofen | 13.3871 | 0.8809 | 0.0000 | 0.0012 | 1.1487 | 1.0171 | up | down |
| 159 | neg_4168 | Avermectin B1a monosaccharide | 0.3333 | 2.6282 | 0.0000 | 0.0000 | 1.1495 | 1.2400 | down | up |
| 160 | neg_4181 | NCGC00385388-01_C21H34O11_(1R,4S,7S,8S,9R,12E,20R,21R,22S,23S)-7,8,21,22,23-Pentahydroxy-12,16-dimethyl-3,5,10,19,24-pentaoxatricyclo[18.3.1.0~4,9~]tetracos-12-en-11-one | 856194269.1792 | 0.6617 | 0.0000 | 0.0000 | 1.1483 | 1.1819 | up | down |
| 161 | neg_4209 | PA(13:0/20:3(8Z,11Z,14Z)-2OH(5,6)) | 0.3339 | 2.6707 | 0.0000 | 0.0000 | 1.1286 | 1.2304 | down | up |
| 162 | neg_4211 | PI(5-iso PGF2VI/22:4(10Z,13Z,16Z,19Z)) | 0.2939 | 2.5905 | 0.0000 | 0.0000 | 1.1503 | 1.2400 | down | up |
| 163 | neg_4281 | Pyridoxaminium(1+) | 0.3887 | 7.7454 | 0.0000 | 0.0000 | 1.1470 | 1.2396 | down | up |
| 164 | neg_4288 | Cer(d16:1/LTE4) | 0.4242 | 2.2432 | 0.0000 | 0.0000 | 1.1501 | 1.2411 | down | up |
| 165 | neg_4289 | PE(15:0/PGJ2) | 0.3633 | 2.5852 | 0.0000 | 0.0000 | 1.1473 | 1.2250 | down | up |
| 166 | neg_4293 | Norfuraneol | 0.2665 | 5.0404 | 0.0000 | 0.0000 | 1.1478 | 1.2393 | down | up |
| 167 | neg_4295 | 2-Phenylethanol glucuronide | 0.1046 | 12.5510 | 0.0000 | 0.0000 | 1.1507 | 1.2416 | down | up |
| 168 | neg_4297 | Homovanillyl alcohol glucuronide | 0.4465 | 3.5767 | 0.0000 | 0.0000 | 1.1404 | 1.2391 | down | up |
| 169 | neg_4305 | Obtustyrene | 3.5519 | 0.8853 | 0.0000 | 0.0002 | 1.1486 | 1.1200 | up | down |
| 170 | neg_4310 | Nevadensin | 0.4984 | 1.3073 | 0.0000 | 0.0000 | 1.1465 | 1.2337 | down | up |
| 171 | neg_4321 | 5-Hydroxyindoleacetate | 0.4425 | 1.6316 | 0.0000 | 0.0000 | 1.1492 | 1.2403 | down | up |
| 172 | neg_4323 | 1-Nitro-5,6-dihydroxy-dihydronaphthalene | 0.4658 | 1.5671 | 0.0000 | 0.0000 | 1.1494 | 1.2384 | down | up |
| 173 | neg_4344 | Isoleucyl-prolyl-arginine-4-nitroanilide | 42.7285 | 0.0078 | 0.0000 | 0.0000 | 1.1498 | 1.2406 | up | down |
| 174 | neg_4348 | DG(18:2(9Z,12Z)/22:6(4Z,7Z,10Z,13Z,16Z,19Z)/0:0) | 0.3749 | 1.7953 | 0.0000 | 0.0000 | 1.1179 | 1.1641 | down | up |
| 175 | neg_4439 | Isopentyl gentiobioside | 0.3671 | 2.4504 | 0.0000 | 0.0000 | 1.1417 | 1.2258 | down | up |
| 176 | neg_4532 | Eriojaposide B | 4.0106 | 0.2925 | 0.0000 | 0.0000 | 1.1458 | 1.2357 | up | down |
| 177 | neg_4550 | Colforsin | 3.6601 | 0.5680 | 0.0000 | 0.0000 | 1.1506 | 1.2402 | up | down |
| 178 | neg_4565 | 3,7-DIHYDROXYFLAVONE | 20.1318 | 0.1588 | 0.0000 | 0.0000 | 1.1506 | 1.2415 | up | down |
| 179 | neg_4607 | Ganglioside GM3 (d18:0/12:0) | 0.4351 | 1.6777 | 0.0000 | 0.0000 | 1.1450 | 1.2254 | down | up |
| 180 | neg_4617 | DG(2:0/PGF2alpha/0:0) | 0.3813 | 1.0353 | 0.0000 | 0.0005 | 1.1507 | 1.0551 | down | up |
| 181 | neg_4633 | DL-b-Hydroxycaprylic acid | 3.8399 | 0.9269 | 0.0000 | 0.0000 | 1.1506 | 1.1627 | up | down |
| 182 | neg_4645 | Acacetin | 33.4218 | 0.1353 | 0.0000 | 0.0000 | 1.1491 | 1.2389 | up | down |
| 183 | neg_4691 | Apramycin | 11.5183 | 0.5195 | 0.0000 | 0.0005 | 1.1015 | 1.0636 | up | down |
| 184 | neg_4700 | Validamine 7-phosphate | 0.2968 | 3.0763 | 0.0000 | 0.0000 | 1.1472 | 1.2367 | down | up |
| 185 | neg_4724 | Avermectin B1b aglycone | 0.1927 | 3.4988 | 0.0000 | 0.0000 | 1.1483 | 1.2378 | down | up |
| 186 | neg_4757 | Lividamine | 0.2272 | 3.1620 | 0.0000 | 0.0000 | 1.1455 | 1.2348 | down | up |
| 187 | neg_4785 | Feruloylagmatine | 2.6235 | 0.5436 | 0.0000 | 0.0000 | 1.1498 | 1.2384 | up | down |
| 188 | neg_4788 | KOdiA-PC | 0.0050 | 20.0305 | 0.0000 | 0.0000 | 1.1496 | 1.1970 | down | up |
| 189 | neg_4796 | PE(22:6(4Z,7Z,10Z,13Z,16Z,19Z)/PGE2) | 0.3920 | 2.0660 | 0.0000 | 0.0000 | 1.1495 | 1.2418 | down | up |
| 190 | neg_4806 | Acetylstrophanthidin | 0.3185 | 2.5530 | 0.0000 | 0.0000 | 1.1373 | 1.2104 | down | up |
| 191 | neg_4810 | 2-(5-Fluoro-3,7,11-trimethyldodeca-2,6,10-trienyl)sulfanylbenzoic acid | 0.0026 | 22.1924 | 0.0000 | 0.0000 | 1.1509 | 1.2411 | down | up |
| 192 | neg_4814 | Cibenzoline | 0.2048 | 1.3683 | 0.0000 | 0.0000 | 1.1477 | 1.1642 | down | up |
| 193 | neg_4896 | homoharringtonine | 6.6284 | 0.8131 | 0.0000 | 0.0002 | 1.1492 | 1.1532 | up | down |
| 194 | neg_4913 | Pirbuterol | 0.3003 | 2.4542 | 0.0000 | 0.0000 | 1.1476 | 1.2403 | down | up |
| 195 | neg_4917 | NCGC00347770-02_C23H32O4_Carda-5,20(22)-dienolide, 3,14-dihydroxy-, (3beta,9xi)- | 0.3152 | 2.3406 | 0.0000 | 0.0000 | 1.1464 | 1.2336 | down | up |
| 196 | neg_4925 | Methyl 3-(2,3-dihydroxy-3-methylbutyl)-4-hydroxybenzoate | 2.9851 | 0.9519 | 0.0000 | 0.0004 | 1.1503 | 1.0737 | up | down |
| 197 | neg_4927 | Genistein | 8.5700 | 0.1515 | 0.0000 | 0.0000 | 1.1508 | 1.2417 | up | down |
| 198 | neg_4949 | 15-methyl-15R-PGE2 | 9.9374 | 0.6826 | 0.0000 | 0.0000 | 1.1505 | 1.2360 | up | down |
| 199 | neg_4952 | Methohexital | 43.3130 | 0.0150 | 0.0000 | 0.0000 | 1.1415 | 1.2319 | up | down |
| 200 | neg_4953 | PI(22:5(4Z,7Z,10Z,13Z,16Z)/PGJ2) | 0.4575 | 1.5051 | 0.0000 | 0.0000 | 1.1461 | 1.2248 | down | up |
| 201 | neg_4960 | (+)-2-(Benzothiophen-4-yl)-N-methyl-N-((1R,2R)-2-pyrrolidin-1-ylcyclohexyl)acetamide | 0.4960 | 1.5579 | 0.0000 | 0.0000 | 1.1476 | 1.2262 | down | up |
| 202 | neg_4961 | PGP(18:0/18:1(11Z)) | 0.4567 | 1.6306 | 0.0000 | 0.0000 | 1.1403 | 1.2055 | down | up |
| 203 | neg_4965 | Tetrahydrocortisone | 5.5929 | 0.6613 | 0.0000 | 0.0000 | 1.1500 | 1.2290 | up | down |
| 204 | neg_50 | Dexamethasone acetate | 3.5819 | 0.5848 | 0.0000 | 0.0000 | 1.1467 | 1.2234 | up | down |
| 205 | neg_5009 | Pyridinoline | 3.0155 | 0.8189 | 0.0000 | 0.0000 | 1.1496 | 1.2318 | up | down |
| 206 | neg_5037 | Peperinic acid | 0.2736 | 1.4144 | 0.0000 | 0.0000 | 1.1497 | 1.2231 | down | up |
| 207 | neg_5047 | Cyclopentadiene | 4.7537 | 0.9100 | 0.0000 | 0.0015 | 1.1496 | 1.0445 | up | down |
| 208 | neg_5063 | Psilostachyin | 0.0921 | 1.9026 | 0.0000 | 0.0000 | 1.1500 | 1.1999 | down | up |
| 209 | neg_5109 | Leucomycin a5 | 0.0006 | 35.8708 | 0.0000 | 0.0039 | 1.1468 | 1.0461 | down | up |
| 210 | neg_5117 | 19-Hydroxyeicosatetraenoic acid | 6.7949 | 0.8168 | 0.0000 | 0.0000 | 1.1507 | 1.2140 | up | down |
| 211 | neg_5130 | ent-7-Oxo-8(14),15-pimaradien-19-oic acid | 157.4802 | 0.5316 | 0.0000 | 0.0000 | 1.1472 | 1.2190 | up | down |
| 212 | neg_5132 | Lucidenic acid D1 | 2.7553 | 0.7517 | 0.0000 | 0.0000 | 1.1463 | 1.2192 | up | down |
| 213 | neg_5144 | 9-deoxy-9-methylene-16,16-dimethyl -PGE2 | 6.8953 | 0.4014 | 0.0000 | 0.0000 | 1.1499 | 1.2398 | up | down |
| 214 | neg_5166 | 7'-Carboxy-gamma-chromanol | 5.6016 | 0.6289 | 0.0000 | 0.0000 | 1.1507 | 1.2393 | up | down |
| 215 | neg_5207 | Solithromycin | 0.4601 | 2.1650 | 0.0000 | 0.0000 | 1.1457 | 1.2409 | down | up |
| 216 | neg_5211 | PS(22:6(4Z,7Z,11E,13Z,15E,19Z)-2OH(10S,17)/18:3(6Z,9Z,12Z)) | 0.1110 | 3.6493 | 0.0000 | 0.0000 | 1.1472 | 1.2280 | down | up |
| 217 | neg_5213 | Methyl 6-[(3S,6S,9S,12R)-3-butan-2-yl-6-[(1-methoxyindol-3-yl)methyl]-2,5,8,11-tetraoxo-1,4,7,10-tetrazabicyclo[10.4.0]hexadecan-9-yl]hexanoate | 21.5885 | 0.6102 | 0.0000 | 0.0000 | 1.1506 | 1.2371 | up | down |
| 218 | neg_5225 | norfentanyl | 3.5709 | 0.7192 | 0.0000 | 0.0000 | 1.1468 | 1.2026 | up | down |
| 219 | neg_5226 | 13,14-Dihydro PGF-1a | 0.4030 | 2.8982 | 0.0000 | 0.0000 | 1.0863 | 1.2102 | down | up |
| 220 | neg_5236 | CDP-DG(i-19:0/PGJ2) | 1141245861.2772 | 0.6819 | 0.0000 | 0.0000 | 1.1476 | 1.1947 | up | down |
| 221 | neg_5254 | Psychosine sulfate | 10.8203 | 0.7594 | 0.0000 | 0.0000 | 1.1494 | 1.2188 | up | down |
| 222 | neg_5272 | Cholylmethionine | 32.8115 | 0.7661 | 0.0000 | 0.0001 | 1.1459 | 1.1074 | up | down |
| 223 | neg_5275 | Chenodeoxycholic acid glycine conjugate | 1510894225.6135 | 0.5655 | 0.0000 | 0.0000 | 1.1477 | 1.2154 | up | down |
| 224 | neg_5280 | Cholylalanine | 5.9919 | 0.7672 | 0.0000 | 0.0000 | 1.1506 | 1.2343 | up | down |
| 225 | neg_5285 | Ethylhexyl salicylate | 0.0769 | 2.0243 | 0.0000 | 0.0000 | 1.1502 | 1.2288 | down | up |
| 226 | neg_5320 | α-CEHC | 3.9577 | 0.3682 | 0.0000 | 0.0000 | 1.1495 | 1.2395 | up | down |
| 227 | neg_5322 | 3-Acetoxyscirpene-4,15-diol | 6.9611 | 0.3120 | 0.0000 | 0.0000 | 1.1477 | 1.2361 | up | down |
| 228 | neg_5333 | Gentamicin | 0.3281 | 1.2256 | 0.0000 | 0.0000 | 1.1506 | 1.2369 | down | up |
| 229 | neg_5339 | Carbocyclic thromboxane A2 | 10.6412 | 0.6873 | 0.0000 | 0.0000 | 1.1490 | 1.2243 | up | down |
| 230 | neg_5344 | Helenalin | 2.0958 | 0.4232 | 0.0000 | 0.0000 | 1.1479 | 1.2400 | up | down |
| 231 | neg_5356 | [12]-Gingerol | 0.0906 | 6.9178 | 0.0001 | 0.0000 | 1.0986 | 1.2236 | down | up |
| 232 | neg_5358 | 16-Acetylgitoxin | 8.2806 | 0.7470 | 0.0000 | 0.0000 | 1.1506 | 1.2367 | up | down |
| 233 | neg_5386 | DG(20:3(8Z,11Z,14Z)-2OH(5,6)/2:0/0:0) | 2.3492 | 0.7653 | 0.0000 | 0.0000 | 1.1471 | 1.2268 | up | down |
| 234 | neg_5401 | Glycodeoxycholic acid | 3.8560 | 0.4738 | 0.0000 | 0.0000 | 1.1508 | 1.2416 | up | down |
| 235 | neg_5412 | 1,5,5,8-Tetramethyl-12-thiabicyclo[9.1.0]dodeca-3,7-diene | 0.1279 | 8.3875 | 0.0000 | 0.0000 | 1.1306 | 1.2356 | down | up |
| 236 | neg_5420 | (±)10-Gingerol | 4.0406 | 0.6417 | 0.0000 | 0.0000 | 1.1501 | 1.2391 | up | down |
| 237 | neg_5443 | Loteprednol | 0.0031 | 160.1163 | 0.0000 | 0.0000 | 1.1509 | 1.2413 | down | up |
| 238 | neg_5461 | 5-Heptyl-2-furanheptanoic acid | 6.2955 | 0.7083 | 0.0000 | 0.0000 | 1.1502 | 1.2322 | up | down |
| 239 | neg_5472 | 1(10),11-Eremophiladiene-2,9-dione | 2.0313 | 0.8743 | 0.0000 | 0.0003 | 1.1407 | 1.0784 | up | down |
| 240 | neg_5488 | Cetilistat | 5.4795 | 0.4312 | 0.0000 | 0.0000 | 1.1508 | 1.2416 | up | down |
| 241 | neg_5491 | 1-(11Z-eicosenoyl)-glycero-3-phosphate | 9.4026 | 0.4013 | 0.0000 | 0.0000 | 1.1385 | 1.1627 | up | down |
| 242 | neg_5506 | (1(10)E,4E,6a,9b)-9-(2-Methylbutanoyloxy)-1(10),4,11(13)-germacratrien-12,6-olide | 2.9395 | 0.8190 | 0.0000 | 0.0000 | 1.1488 | 1.2077 | up | down |
| 243 | neg_5508 | NCGC00380753-01_C16H28O12_6-O-Isobutyryl-alpha-D-glucopyranosyl alpha-D-glucopyranoside | 0.2308 | 1.6620 | 0.0000 | 0.0000 | 1.1498 | 1.2328 | down | up |
| 244 | neg_5529 | 4-Methyl-2-methylene-1-(1methylethylidene)-cyclohexane | 4.8373 | 0.2752 | 0.0000 | 0.0001 | 1.0740 | 1.1038 | up | down |
| 245 | neg_5566 | PE(24:0/20:3(8Z,11Z,14Z)-2OH(5,6)) | 2.5452 | 0.5211 | 0.0001 | 0.0009 | 1.0521 | 1.1126 | up | down |
| 246 | neg_5591 | 9,10,13-Trihydroxystearic acid | 0.1604 | 4.6023 | 0.0000 | 0.0000 | 1.1484 | 1.2414 | down | up |
| 247 | neg_5605 | 19(R)-hydroxy-PGF1α | 0.1072 | 12.5343 | 0.0000 | 0.0000 | 1.1431 | 1.2407 | down | up |
| 248 | neg_5612 | 13'-Carboxy-gamma-tocopherol | 2.1562 | 0.8420 | 0.0000 | 0.0001 | 1.1453 | 1.1894 | up | down |
| 249 | neg_5623 | CDP-DG(PGJ2/i-22:0) | 0.0039 | 43.2498 | 0.0000 | 0.0000 | 1.1496 | 1.2096 | down | up |
| 250 | neg_5630 | Squamosinin A | 739.7317 | 0.4370 | 0.0000 | 0.0000 | 1.1399 | 1.1992 | up | down |
| 251 | neg_5638 | Vulgarol | 3.2478 | 0.8741 | 0.0000 | 0.0003 | 1.1478 | 1.1671 | up | down |
| 252 | neg_5645 | Maraviroc | 2.1339 | 0.7094 | 0.0000 | 0.0001 | 1.1199 | 1.1628 | up | down |
| 253 | neg_5651 | Glycochenodeoxycholate | 2.0676 | 0.9270 | 0.0000 | 0.0000 | 1.1504 | 1.2120 | up | down |
| 254 | neg_5661 | Caproylcholine | 10.3434 | 0.8430 | 0.0000 | 0.0000 | 1.1503 | 1.2124 | up | down |
| 255 | neg_5672 | 1-Acetoxy-2-hydroxy-16-heptadecyn-4-one | 6.4227 | 0.7610 | 0.0000 | 0.0000 | 1.1502 | 1.2220 | up | down |
| 256 | neg_5680 | Physapubescin | 0.2347 | 8.9514 | 0.0000 | 0.0000 | 1.0951 | 1.2314 | down | up |
| 257 | neg_5681 | Methyl (Z,Z)-5,8-tetradecadienoate | 33.0928 | 0.4451 | 0.0000 | 0.0000 | 1.1491 | 1.2354 | up | down |
| 258 | neg_5688 | Parthenin | 0.1022 | 1.4082 | 0.0000 | 0.0000 | 1.1508 | 1.2221 | down | up |
| 259 | neg_5694 | Coprocholic acid | 0.3667 | 2.3114 | 0.0000 | 0.0000 | 1.1471 | 1.2393 | down | up |
| 260 | neg_5737 | 14-Deoxyandrographolide | 2.4225 | 0.8211 | 0.0000 | 0.0000 | 1.1459 | 1.1852 | up | down |
| 261 | neg_5739 | Ganglioside GM3 (d18:1/16:0) | 2.1011 | 0.4072 | 0.0008 | 0.0001 | 1.0455 | 1.1282 | up | down |
| 262 | neg_5748 | Laurenobiolide | 0.1899 | 1.9632 | 0.0000 | 0.0000 | 1.1502 | 1.2360 | down | up |
| 263 | neg_5815 | (Z)-3-Methyl-3-decen-1-ol | 4.5271 | 0.8252 | 0.0000 | 0.0000 | 1.1498 | 1.2142 | up | down |
| 264 | neg_5838 | LysoPC(14:0/0:0) | 0.4222 | 1.2798 | 0.0000 | 0.0000 | 1.1499 | 1.2240 | down | up |
| 265 | neg_5841 | Taurolithocholic acid 3-sulfate | 0.3988 | 1.3534 | 0.0000 | 0.0000 | 1.1463 | 1.2286 | down | up |
| 266 | neg_5846 | Desglucocoroloside | 0.3109 | 1.3957 | 0.0000 | 0.0019 | 1.1164 | 1.0294 | down | up |
| 267 | neg_5857 | 4,7-Megastigmadien-9-ol | 15.8039 | 0.8278 | 0.0000 | 0.0000 | 1.1508 | 1.2300 | up | down |
| 268 | neg_5859 | 5-((6-((Aminomethyl)amino)-1-oxohexyl)amino)pentanoic acid | 28.0803 | 0.8422 | 0.0000 | 0.0000 | 1.1499 | 1.1794 | up | down |
| 269 | neg_5877 | LysoPE(22:5(4Z,7Z,10Z,13Z,16Z)/0:0) | 0.2665 | 1.6506 | 0.0000 | 0.0011 | 1.1380 | 1.0183 | down | up |
| 270 | neg_5931 | 5beta-Cholestane-3alpha,7alpha,24,26-tetrol | 3.1886 | 0.8120 | 0.0000 | 0.0000 | 1.1494 | 1.2143 | up | down |
| 271 | neg_5969 | lpc(16-1_0-0) | 0.2837 | 1.3470 | 0.0000 | 0.0000 | 1.1425 | 1.1694 | down | up |
| 272 | neg_6022 | Fumonisin AK1 | 3.4122 | 0.7740 | 0.0000 | 0.0000 | 1.1508 | 1.2399 | up | down |
| 273 | neg_6023 | Jubanine C | 3.3609 | 0.6805 | 0.0000 | 0.0000 | 1.1508 | 1.2404 | up | down |
| 274 | neg_6165 | Prostaglandin A1 ethyl ester | 9.6919 | 0.1929 | 0.0000 | 0.0002 | 1.1283 | 1.1620 | up | down |
| 275 | neg_6167 | Gymnodimine | 0.4929 | 1.0638 | 0.0000 | 0.0017 | 1.1488 | 1.0210 | down | up |
| 276 | neg_6191 | Dicarbine | 5.9106 | 0.4814 | 0.0000 | 0.0000 | 1.1276 | 1.2054 | up | down |
| 277 | neg_6328 | PG 17_0-16_1-d5 | 3.8868 | 0.8257 | 0.0000 | 0.0000 | 1.1464 | 1.1689 | up | down |
| 278 | neg_6346 | 3b,17b-Dihydroxyetiocholane | 214465891.6786 | 0.0543 | 0.0001 | 0.0000 | 1.1088 | 1.1730 | up | down |
| 279 | neg_6347 | 2-Isopropyl-1,4-hexadiene | 1732029373.9521 | 0.4695 | 0.0000 | 0.0002 | 1.1229 | 1.1080 | up | down |
| 280 | neg_6385 | (2S,4R)-p-Mentha-1(7),5-dien-2-ol | 4.1487 | 0.4099 | 0.0000 | 0.0000 | 1.1399 | 1.2253 | up | down |
| 281 | neg_6514 | LysoPC(24:1(15Z)/0:0) | 3.5180 | 0.5693 | 0.0001 | 0.0040 | 1.0392 | 1.0109 | up | down |
| 282 | neg_6604 | 3-hydroxy-tetradecanoic acid | 5.7752 | 0.8387 | 0.0000 | 0.0000 | 1.1500 | 1.2115 | up | down |
| 283 | neg_6611 | PE(16_1e_15-HETE) | 2.6274 | 0.8952 | 0.0000 | 0.0020 | 1.1435 | 1.0028 | up | down |
| 284 | neg_6635 | 3-Heptadeca-1,3-dienyloxirene-2-carboxylic acid | 2.7626 | 0.5821 | 0.0000 | 0.0000 | 1.1347 | 1.1733 | up | down |
| 285 | neg_6754 | Cholic acid glucuronide | 0.4910 | 1.6666 | 0.0000 | 0.0000 | 1.1505 | 1.2411 | down | up |
| 286 | neg_6758 | Myrcenol | 2.1160 | 0.7938 | 0.0000 | 0.0001 | 1.1363 | 1.1147 | up | down |
| 287 | neg_6765 | 13(S)-HODE | 88.0362 | 0.4967 | 0.0000 | 0.0000 | 1.1496 | 1.2179 | up | down |
| 288 | neg_6775 | D-1,5-Anhydrofructose | 0.2711 | 2.3201 | 0.0000 | 0.0000 | 1.1488 | 1.2382 | down | up |
| 289 | neg_6776 | Corchorosol A | 0.3840 | 1.7705 | 0.0000 | 0.0000 | 1.1446 | 1.2263 | down | up |
| 290 | neg_6778 | 3-Butyn-1-al | 0.1013 | 6.1726 | 0.0000 | 0.0000 | 1.1474 | 1.2284 | down | up |
| 291 | neg_6784 | 16-Hydroxyhexadecanoic acid | 2.2836 | 0.6933 | 0.0000 | 0.0000 | 1.1482 | 1.2307 | up | down |
| 292 | neg_6791 | 3-Methylene-1,5,5-trimethylcyclohexene | 0.2247 | 1.6162 | 0.0000 | 0.0025 | 1.1327 | 1.0342 | down | up |
| 293 | neg_6793 | ent-16b,19-Kauranediol 19-acetate | 0.2439 | 2.0787 | 0.0000 | 0.0000 | 1.1323 | 1.1866 | down | up |
| 294 | neg_6794 | Pregnanetriol 3a-O-b-D-glucuronide | 0.2491 | 1.8114 | 0.0000 | 0.0000 | 1.1490 | 1.2347 | down | up |
| 295 | neg_6799 | Nordeoxycholic acid | 0.2560 | 2.2726 | 0.0000 | 0.0000 | 1.1501 | 1.2270 | down | up |
| 296 | neg_6800 | Ascorbyl stearate | 0.4480 | 2.0888 | 0.0000 | 0.0000 | 1.1477 | 1.2368 | down | up |
| 297 | neg_6846 | 3-Oxohexadecanoic acid | 4.9127 | 0.8824 | 0.0000 | 0.0003 | 1.1488 | 1.0787 | up | down |
| 298 | neg_6848 | 1,2,3-Trihydroxyhenicos-12-en-4-one | 2.2536 | 0.5564 | 0.0000 | 0.0000 | 1.1413 | 1.2262 | up | down |
| 299 | neg_6856 | Eicosanedioic acid | 4.2885 | 0.7774 | 0.0000 | 0.0000 | 1.1483 | 1.2122 | up | down |
| 300 | neg_6859 | DG(8:0/0:0/16:0) | 2.5099 | 0.7636 | 0.0000 | 0.0006 | 1.1298 | 1.1051 | up | down |
| 301 | neg_6863 | Androstan-3alpha,17beta-diol | 0.2610 | 3.7562 | 0.0004 | 0.0005 | 1.0561 | 1.1326 | down | up |
| 302 | neg_6912 | 20a,22b-Dihydroxycholesterol | 72.4520 | 0.4781 | 0.0000 | 0.0000 | 1.1502 | 1.2382 | up | down |
| 303 | neg_6925 | Goshuyic acid | 5.3266 | 0.4009 | 0.0000 | 0.0000 | 1.1457 | 1.2317 | up | down |
| 304 | neg_7000 | Docosa-2,4,6,8-tetraenoic acid | 2.0061 | 0.8295 | 0.0000 | 0.0000 | 1.1450 | 1.1656 | up | down |
| 305 | neg_7018 | 3-Decenoic acid | 0.1519 | 2.9766 | 0.0000 | 0.0000 | 1.1224 | 1.1800 | down | up |
| 306 | neg_7019 | Palmitoyl glucuronide | 0.2741 | 1.7806 | 0.0000 | 0.0000 | 1.1473 | 1.2288 | down | up |
| 307 | neg_7022 | 17alpha,20alpha-Dihydroxycholesterol | 137.5245 | 0.4483 | 0.0000 | 0.0000 | 1.1507 | 1.2402 | up | down |
| 308 | neg_7030 | docosanedioate | 4.7750 | 0.8442 | 0.0000 | 0.0017 | 1.1392 | 1.0007 | up | down |
| 309 | neg_71 | Ala-Met-OH | 0.4243 | 1.9068 | 0.0001 | 0.0014 | 1.0789 | 1.0786 | down | up |
| 310 | neg_7186 | Methyl 2-(10-heptadecenyl)-6-hydroxybenzoate | 5.2574 | 0.6189 | 0.0000 | 0.0003 | 1.1245 | 1.1315 | up | down |
| 311 | neg_7196 | Hept-2-en-1-yl isovalerate | 3.3836 | 0.7694 | 0.0000 | 0.0000 | 1.1508 | 1.2330 | up | down |
| 312 | neg_7197 | 1-Hydroxy-1-phenyl-3-octadecanone | 4.3452 | 0.5100 | 0.0000 | 0.0000 | 1.1488 | 1.2348 | up | down |
| 313 | neg_7214 | Heneicosanoic acid | 31.9940 | 0.5093 | 0.0000 | 0.0000 | 1.1432 | 1.2125 | up | down |
| 314 | neg_7238 | (±)-(E)-3-Methyl-4-decen-1-yl acetate | 6.1153 | 0.6771 | 0.0000 | 0.0001 | 1.1418 | 1.1195 | up | down |
| 315 | neg_7290 | delta-Terpineol | 195.3936 | 0.5933 | 0.0000 | 0.0000 | 1.1489 | 1.2236 | up | down |
| 316 | neg_7295 | 4,6-Tricosanedione | 5.7971 | 0.6097 | 0.0000 | 0.0000 | 1.1473 | 1.2170 | up | down |
| 317 | neg_7296 | Methyl-[12]-gingerdiol | 3.1534 | 0.7972 | 0.0000 | 0.0000 | 1.1488 | 1.2109 | up | down |
| 318 | neg_7333 | 2-Hexenyl octanoate | 59.8620 | 0.2471 | 0.0000 | 0.0000 | 1.1249 | 1.1708 | up | down |
| 319 | neg_7340 | Erucoylacetone | 8.1199 | 0.5155 | 0.0000 | 0.0000 | 1.1496 | 1.2348 | up | down |
| 320 | neg_7358 | Polyoxyethylene (600) monoricinoleate | 0.1944 | 1.7984 | 0.0000 | 0.0000 | 1.1176 | 1.1409 | down | up |
| 321 | neg_7364 | 5-(12-Nonadecenyl)-1,3-benzenediol | 3.3548 | 0.6959 | 0.0000 | 0.0000 | 1.1495 | 1.2295 | up | down |
| 322 | neg_7435 | 6,10,14-Trimethyl-2-methylenepentadecanal | 0.2958 | 1.3924 | 0.0000 | 0.0013 | 1.1315 | 1.0557 | down | up |
| 323 | neg_7504 | 10,20-Dihydroxyeicosanoic acid | 5.5163 | 0.6910 | 0.0000 | 0.0000 | 1.1473 | 1.2029 | up | down |
| 324 | neg_7512 | cis-Quinceoxepane | 2.7358 | 0.6136 | 0.0000 | 0.0000 | 1.1369 | 1.1939 | up | down |
| 325 | neg_7576 | RIOPROSTIL | 0.0729 | 2.4605 | 0.0000 | 0.0000 | 1.1487 | 1.2257 | down | up |
| 326 | neg_7606 | Mosinone A | 4.8977 | 0.8279 | 0.0000 | 0.0000 | 1.1488 | 1.1573 | up | down |
| 327 | neg_7615 | MG(16:0/0:0/0:0) | 0.1463 | 3.0914 | 0.0000 | 0.0000 | 1.1452 | 1.2314 | down | up |
| 328 | neg_7632 | Auberganol | 5.5771 | 0.4997 | 0.0000 | 0.0000 | 1.1482 | 1.2321 | up | down |
| 329 | neg_7669 | lpe(15-0_0-0) | 0.4213 | 1.3997 | 0.0000 | 0.0014 | 1.0942 | 1.0206 | down | up |
| 330 | neg_7670 | MMV687801 | 5.1913 | 0.7817 | 0.0000 | 0.0001 | 1.1410 | 1.1227 | up | down |
| 331 | neg_78 | Rosmarinate | 0.4345 | 1.7944 | 0.0000 | 0.0001 | 1.0895 | 1.1333 | down | up |
| 332 | neg_7901 | Triptohypol F | 5.3033 | 0.7753 | 0.0000 | 0.0000 | 1.1487 | 1.1973 | up | down |
| 333 | neg_7910 | Germacrenone | 3.9085 | 0.6952 | 0.0000 | 0.0000 | 1.1490 | 1.2126 | up | down |
| 334 | neg_7931 | 4alpha-hydroxymethyl,4beta,14alpha-dimethyl-9beta,19-cyclo-5alpha-ergost-24(241)-en-3beta-ol | 4.1686 | 0.7508 | 0.0000 | 0.0000 | 1.1482 | 1.1944 | up | down |
| 335 | neg_891 | L-Glutamine | 0.4427 | 1.1064 | 0.0000 | 0.0000 | 1.1489 | 1.1508 | down | up |
| 336 | neg_99 | dTDP-D-glucuronate | 0.4802 | 1.6867 | 0.0000 | 0.0008 | 1.1300 | 1.1104 | down | up |
| 337 | pos_10 | 7alpha-Hydroxyandrost-4-ene-3,17-dione | 2.4957 | 0.5199 | 0.0000 | 0.0000 | 1.1123 | 1.1573 | up | down |
| 338 | pos_1011 | Muramic acid | 2.7554 | 0.7116 | 0.0000 | 0.0002 | 1.1253 | 1.0851 | up | down |
| 339 | pos_1035 | 2-amino-8-oxo-9,10-epoxy-decanoic acid | 33.1716 | 0.6793 | 0.0000 | 0.0000 | 1.1497 | 1.2293 | up | down |
| 340 | pos_1036 | 4-[(2,4-Dihydroxy-3,3-dimethylbutanoyl)amino]butanoic acid | 1041918619.0486 | 0.6843 | 0.0000 | 0.0000 | 1.1480 | 1.1989 | up | down |
| 341 | pos_1049 | L-Norleucine | 6.6025 | 0.9006 | 0.0000 | 0.0007 | 1.1490 | 1.0574 | up | down |
| 342 | pos_1051 | 3-Hydroxybutyrylcarnitine | 12.4441 | 0.7346 | 0.0000 | 0.0000 | 1.1501 | 1.2245 | up | down |
| 343 | pos_1052 | 2-Hydroxyamino-1-methyl-6-phenylimidazo[4,5-b]pyridine | 13.5792 | 0.7260 | 0.0000 | 0.0000 | 1.1501 | 1.2205 | up | down |
| 344 | pos_1053 | L-Pipecolate | 1828071175.6998 | 0.6892 | 0.0000 | 0.0000 | 1.1497 | 1.2052 | up | down |
| 345 | pos_1056 | N-(1-Deoxy-1-fructosyl)leucine | 12.8600 | 0.7389 | 0.0000 | 0.0000 | 1.1491 | 1.2096 | up | down |
| 346 | pos_1063 | N-(1-Deoxy-1-fructosyl)glycine | 515598181.9999 | 0.6366 | 0.0000 | 0.0000 | 1.1471 | 1.2005 | up | down |
| 347 | pos_1064 | 7-Methylxanthine | 4.8126 | 0.5997 | 0.0000 | 0.0002 | 1.1257 | 1.0838 | up | down |
| 348 | pos_1065 | n-(1-deoxy-1-fructosyl)phenylalanine | 3.2256 | 0.6343 | 0.0000 | 0.0000 | 1.1506 | 1.2394 | up | down |
| 349 | pos_1075 | Indoleacetaldehyde | 0.2389 | 4.0052 | 0.0000 | 0.0000 | 1.1440 | 1.2409 | down | up |
| 350 | pos_1080 | 5-Hydroxyindoleacetic acid | 0.4485 | 1.6115 | 0.0000 | 0.0000 | 1.1305 | 1.1908 | down | up |
| 351 | pos_1096 | 7-Mercaptoheptanoylthreonine | 4.6496 | 0.5574 | 0.0000 | 0.0000 | 1.1496 | 1.2373 | up | down |
| 352 | pos_11 | Acuminoside | 7.9613 | 0.1971 | 0.0000 | 0.0000 | 1.1444 | 1.2335 | up | down |
| 353 | pos_1108 | Daumone | 14.1344 | 0.3800 | 0.0000 | 0.0000 | 1.1463 | 1.2292 | up | down |
| 354 | pos_1120 | virginiamycin m1 | 2.1984 | 0.7802 | 0.0000 | 0.0000 | 1.1423 | 1.1689 | up | down |
| 355 | pos_1123 | 5,6,7,8-Tetrahydromonapterin | 9.3097 | 0.4478 | 0.0001 | 0.0003 | 1.1124 | 1.0758 | up | down |
| 356 | pos_1124 | 3-Carboxy-2,3,4,9-tetrahydro-1H-pyrido[3,4-b]indole-1-propanoic acid | 2.3369 | 0.7359 | 0.0000 | 0.0002 | 1.1191 | 1.1567 | up | down |
| 357 | pos_1125 | Formylfusarochromanone | 2.2197 | 0.7069 | 0.0000 | 0.0000 | 1.1413 | 1.2137 | up | down |
| 358 | pos_1132 | 2,3-Butanediol glucoside | 0.1732 | 3.8217 | 0.0000 | 0.0000 | 1.1456 | 1.2131 | down | up |
| 359 | pos_1137 | Pentenylbenzene | 0.3246 | 1.7057 | 0.0000 | 0.0000 | 1.1182 | 1.2101 | down | up |
| 360 | pos_1140 | Cytidine 5'-{[hydroxy(2-hydroxypropyl)phosphonoyl]phosphate} | 0.2869 | 1.8322 | 0.0000 | 0.0000 | 1.1451 | 1.2270 | down | up |
| 361 | pos_1159 | L-Tryptophan | 0.3280 | 1.6187 | 0.0000 | 0.0000 | 1.1429 | 1.2196 | down | up |
| 362 | pos_1163 | 5-(Phenylethynyl)furan-2-carboxylic acid | 2.8288 | 0.6850 | 0.0000 | 0.0000 | 1.1487 | 1.2364 | up | down |
| 363 | pos_1168 | Asp Asp Asp | 2.8504 | 0.7039 | 0.0000 | 0.0000 | 1.1495 | 1.2345 | up | down |
| 364 | pos_1177 | Aminomethyl fluorescein | 2.5527 | 0.7383 | 0.0000 | 0.0000 | 1.1497 | 1.2350 | up | down |
| 365 | pos_1184 | 2-(Formamido)-N1-(5'-phosphoribosyl)acetamidine | 3.2225 | 0.7012 | 0.0000 | 0.0000 | 1.1452 | 1.2224 | up | down |
| 366 | pos_1193 | 7-Methyl-2'-deoxyguanosine-3'-monophosphate | 2.0809 | 0.8154 | 0.0000 | 0.0000 | 1.1407 | 1.1709 | up | down |
| 367 | pos_1195 | Nitrobenzyl-6-thioinosine | 0.4890 | 1.4383 | 0.0000 | 0.0000 | 1.1465 | 1.2146 | down | up |
| 368 | pos_1196 | dTTP | 9.3221 | 0.6210 | 0.0000 | 0.0012 | 1.1230 | 1.0166 | up | down |
| 369 | pos_1239 | (2S)-Lactyl-2-diphospho-5'-guanosine | 0.2981 | 1.9223 | 0.0000 | 0.0000 | 1.1288 | 1.1732 | down | up |
| 370 | pos_1282 | S-[2-(N7-Guanyl)ethyl]-N-acetyl-L-cysteine | 2.8445 | 0.2377 | 0.0000 | 0.0000 | 1.1241 | 1.2213 | up | down |
| 371 | pos_13 | Tuberonic Acid | 5.9902 | 0.4746 | 0.0000 | 0.0000 | 1.1455 | 1.2259 | up | down |
| 372 | pos_1302 | Adenosine tetraphosphate | 5.1938 | 0.3806 | 0.0000 | 0.0000 | 1.1482 | 1.2329 | up | down |
| 373 | pos_1308 | Paraldehyde | 0.4173 | 1.9625 | 0.0000 | 0.0000 | 1.1437 | 1.2352 | down | up |
| 374 | pos_1310 | N'-nitrosonornicotine | 0.3638 | 2.4467 | 0.0000 | 0.0000 | 1.1318 | 1.1938 | down | up |
| 375 | pos_1312 | hexaethylene-glycol | 0.4022 | 1.9657 | 0.0000 | 0.0000 | 1.1475 | 1.2397 | down | up |
| 376 | pos_1314 | N-Lauroyl Glutamine | 0.3590 | 2.1994 | 0.0000 | 0.0000 | 1.1496 | 1.2402 | down | up |
| 377 | pos_1315 | Cotinine | 43.8482 | 0.0093 | 0.0000 | 0.0000 | 1.1452 | 1.2363 | up | down |
| 378 | pos_1317 | 6''-O-Carbamoylkanamycin A | 154719570.8363 | 0.0387 | 0.0000 | 0.0000 | 1.1229 | 1.2089 | up | down |
| 379 | pos_1318 | (R)-Acetoin | 0.4123 | 1.9667 | 0.0000 | 0.0000 | 1.1432 | 1.2232 | down | up |
| 380 | pos_1319 | 6'''-Deamino-6'''-oxoneomycin C | 3.9824 | 0.2475 | 0.0000 | 0.0000 | 1.1412 | 1.2347 | up | down |
| 381 | pos_1324 | Ronacaleret | 4.3447 | 0.3790 | 0.0000 | 0.0000 | 1.1478 | 1.2345 | up | down |
| 382 | pos_1327 | 1-Acetylindole | 0.0777 | 5.6149 | 0.0000 | 0.0000 | 1.1469 | 1.2256 | down | up |
| 383 | pos_1328 | Xylostasin | 369926794.7172 | 0.1552 | 0.0000 | 0.0000 | 1.1419 | 1.2241 | up | down |
| 384 | pos_1332 | Pelargonidin 3-arabinoside | 478935125.3332 | 0.3240 | 0.0000 | 0.0000 | 1.1479 | 1.2314 | up | down |
| 385 | pos_1336 | Violaceinic acid | 0.1837 | 12.7916 | 0.0000 | 0.0000 | 1.1466 | 1.2407 | down | up |
| 386 | pos_1341 | 4h-Benzoxazine | 2.4548 | 0.5305 | 0.0000 | 0.0000 | 1.1476 | 1.2362 | up | down |
| 387 | pos_1347 | 10-Piperazinylpropylphenothiazine | 0.3245 | 2.4912 | 0.0000 | 0.0000 | 1.1421 | 1.2328 | down | up |
| 388 | pos_1364 | Ethyl 3-hydroxybutyrate | 0.4209 | 1.9502 | 0.0000 | 0.0000 | 1.1430 | 1.2373 | down | up |
| 389 | pos_1367 | Heptaethylene glycol | 0.4026 | 1.9928 | 0.0000 | 0.0000 | 1.1482 | 1.2393 | down | up |
| 390 | pos_1373 | Diprotin A | 0.1512 | 4.8539 | 0.0000 | 0.0000 | 1.1490 | 1.2400 | down | up |
| 391 | pos_1376 | val-gln | 7.0021 | 0.4299 | 0.0000 | 0.0000 | 1.1441 | 1.2256 | up | down |
| 392 | pos_1379 | Benzoic acid | 0.3252 | 6.8483 | 0.0000 | 0.0000 | 1.1465 | 1.2401 | down | up |
| 393 | pos_1380 | Hippuric acid | 0.0000 | 299089118.1367 | 0.0000 | 0.0000 | 1.1276 | 1.2376 | down | up |
| 394 | pos_1385 | Neodiospyrin | 0.0000 | 427500660.8242 | 0.0009 | 0.0000 | 1.0515 | 1.2274 | down | up |
| 395 | pos_1387 | Benzene | 0.0436 | 61.6040 | 0.0000 | 0.0000 | 1.1240 | 1.2238 | down | up |
| 396 | pos_1390 | Glu-Val-Phe | 0.3546 | 2.3928 | 0.0000 | 0.0000 | 1.1466 | 1.2379 | down | up |
| 397 | pos_1391 | 4-(3-Pyridyl)-3-butenoic acid | 0.2550 | 1.4398 | 0.0000 | 0.0000 | 1.1446 | 1.1769 | down | up |
| 398 | pos_1394 | 5-Hydroxy-6-methoxyindole glucuronide | 0.2685 | 1.6693 | 0.0000 | 0.0000 | 1.1483 | 1.2320 | down | up |
| 399 | pos_14 | L-Acetopine | 3.3873 | 0.6502 | 0.0000 | 0.0007 | 1.1136 | 1.0871 | up | down |
| 400 | pos_1404 | 3-Dehydroshikimate | 2.4686 | 0.7529 | 0.0000 | 0.0010 | 1.1259 | 1.0256 | up | down |
| 401 | pos_1405 | Benzyl O-[arabinofuranosyl-(1->6)-glucoside] | 602259889.5786 | 0.6002 | 0.0000 | 0.0000 | 1.1444 | 1.1844 | up | down |
| 402 | pos_1408 | TRIETHYLENE GLYCOL | 0.4327 | 1.9989 | 0.0000 | 0.0000 | 1.1444 | 1.2337 | down | up |
| 403 | pos_1409 | Octaethylene glycol | 0.3948 | 2.0873 | 0.0000 | 0.0000 | 1.1477 | 1.2398 | down | up |
| 404 | pos_1411 | N-(R)-[2-(Hydroxyaminocarbonyl)methyl]-4-methylpentanoyl-L-t-butyl-alanyl-L-alanine, 2-aminoethyl Amide | 0.3519 | 2.2328 | 0.0000 | 0.0000 | 1.1464 | 1.2379 | down | up |
| 405 | pos_1416 | N-Docosahexaenoyl Lysine | 0.4438 | 2.0479 | 0.0000 | 0.0000 | 1.1003 | 1.1839 | down | up |
| 406 | pos_1417 | Acetoin | 0.4235 | 2.0017 | 0.0000 | 0.0000 | 1.1453 | 1.2295 | down | up |
| 407 | pos_1434 | beta-Tocotrienol | 0.3298 | 2.3411 | 0.0000 | 0.0000 | 1.1362 | 1.2051 | down | up |
| 408 | pos_1449 | cis-1,2-Dihydro-3-ethylcatechol | 3.8966 | 0.3185 | 0.0000 | 0.0000 | 1.1444 | 1.2325 | up | down |
| 409 | pos_1460 | Methyl cellulose | 0.4038 | 2.1157 | 0.0000 | 0.0000 | 1.1473 | 1.2404 | down | up |
| 410 | pos_1565 | 4-Amino-1-piperidinecarboxylic acid | 2.0918 | 0.6339 | 0.0000 | 0.0006 | 1.0712 | 1.0482 | up | down |
| 411 | pos_1666 | 8,8a-Deoxyoleandolide | 0.3282 | 3.5636 | 0.0000 | 0.0000 | 1.1109 | 1.2095 | down | up |
| 412 | pos_1708 | D-Urobilinogen | 2.9266 | 0.7763 | 0.0000 | 0.0000 | 1.1469 | 1.1991 | up | down |
| 413 | pos_1711 | Linalool oxide D 3-[apiosyl-(1->6)-glucoside] | 17.9948 | 0.6341 | 0.0000 | 0.0002 | 1.1340 | 1.0943 | up | down |
| 414 | pos_1717 | LPE 17_0-d5 | 0.1098 | 6.6691 | 0.0000 | 0.0000 | 1.1401 | 1.2391 | down | up |
| 415 | pos_1720 | Etimicin | 0.3547 | 2.3258 | 0.0000 | 0.0000 | 1.1456 | 1.2371 | down | up |
| 416 | pos_1755 | 4-Hydroxy-5-phenyltetrahydro-1,3-oxazin-2-one | 8.6354 | 0.3930 | 0.0000 | 0.0000 | 1.1397 | 1.2156 | up | down |
| 417 | pos_1860 | 4-formyl Indole | 0.3326 | 1.4861 | 0.0000 | 0.0000 | 1.1496 | 1.2136 | down | up |
| 418 | pos_1861 | 6-Hydroxymethylpterin | 0.2216 | 1.8096 | 0.0000 | 0.0000 | 1.1457 | 1.2006 | down | up |
| 419 | pos_1862 | 5-Methoxyindole-2-carboxylic acid | 0.2482 | 1.8161 | 0.0000 | 0.0000 | 1.1490 | 1.2097 | down | up |
| 420 | pos_1863 | Tyrosyl-Serine | 0.3413 | 1.4453 | 0.0000 | 0.0000 | 1.1452 | 1.2088 | down | up |
| 421 | pos_1864 | (2,5-Dioxopyrrolidin-1-yl) 5-(2-hydroxyethoxy)-4-oxopentanoate | 0.3325 | 1.3879 | 0.0000 | 0.0017 | 1.1351 | 1.0176 | down | up |
| 422 | pos_1865 | Aloxistatin | 0.2753 | 2.8811 | 0.0000 | 0.0000 | 1.1462 | 1.2372 | down | up |
| 423 | pos_1866 | Prostaglandin I2 | 0.1724 | 5.2046 | 0.0000 | 0.0000 | 1.1476 | 1.2368 | down | up |
| 424 | pos_1880 | N-[2-[5-[[3-(2-Aminoethyl)-1H-indol-5-yl]-methoxymethoxy]-1H-indol-3-yl]ethyl]-N-propan-2-ylpropan-2-amine | 0.3040 | 2.6265 | 0.0000 | 0.0000 | 1.1488 | 1.2401 | down | up |
| 425 | pos_1889 | (1S,2S,3S,4R)-3-(1-Acetamido-2-ethylbutyl)-4-(diaminomethylideneamino)-2-hydroxycyclopentane-1-carboxylic acid | 2.5154 | 0.7306 | 0.0000 | 0.0000 | 1.1470 | 1.2227 | up | down |
| 426 | pos_1912 | apicidin | 0.2159 | 4.5466 | 0.0000 | 0.0000 | 1.1438 | 1.2406 | down | up |
| 427 | pos_1915 | Neuromedin C 1-8 | 2.0459 | 0.7749 | 0.0000 | 0.0000 | 1.1428 | 1.1819 | up | down |
| 428 | pos_1921 | L-Phenylalanine | 2.9391 | 0.5483 | 0.0000 | 0.0000 | 1.1482 | 1.2373 | up | down |
| 429 | pos_1922 | L-Tyrosine | 71.2882 | 0.1035 | 0.0000 | 0.0000 | 1.1443 | 1.2273 | up | down |
| 430 | pos_1960 | beta-Acetyldigoxin | 5.0758 | 0.7655 | 0.0000 | 0.0000 | 1.1454 | 1.1328 | up | down |
| 431 | pos_1961 | Tezacitabine | 0.1520 | 39.1487 | 0.0000 | 0.0000 | 1.1011 | 1.2383 | down | up |
| 432 | pos_1964 | Gentamicin C1a | 0.3320 | 2.5718 | 0.0000 | 0.0000 | 1.1309 | 1.2300 | down | up |
| 433 | pos_1965 | Tert-butyl N-[2-(prop-2-enamido)ethyl]carbamate | 2.6998 | 0.7133 | 0.0000 | 0.0000 | 1.1454 | 1.1830 | up | down |
| 434 | pos_1968 | Leu His Lys His | 0.2966 | 2.7620 | 0.0000 | 0.0000 | 1.1476 | 1.2408 | down | up |
| 435 | pos_1972 | Indole-3-carboxaldehyde | 0.1394 | 5.1905 | 0.0000 | 0.0000 | 1.1496 | 1.2394 | down | up |
| 436 | pos_1974 | (Z)-7-[(1S,4R,6R)-4-[(E)-Oct-6-enyl]-2,3-diazabicyclo[2.2.1]hept-2-en-6-yl]hept-5-enoic acid | 2.9035 | 0.7572 | 0.0000 | 0.0000 | 1.1466 | 1.2052 | up | down |
| 437 | pos_1976 | His Arg Phe Lys | 0.3090 | 3.1372 | 0.0000 | 0.0000 | 1.1484 | 1.2413 | down | up |
| 438 | pos_1978 | Gluten exorphin C | 0.1928 | 5.1516 | 0.0000 | 0.0000 | 1.1462 | 1.2390 | down | up |
| 439 | pos_1990 | Docosapentaenoic acid (22n-3) | 0.3094 | 2.8700 | 0.0000 | 0.0000 | 1.1451 | 1.2318 | down | up |
| 440 | pos_1997 | Palmitoylcarnitine | 0.2562 | 3.2449 | 0.0000 | 0.0000 | 1.1429 | 1.2361 | down | up |
| 441 | pos_2007 | Phenylalanyltryptophan | 0.3619 | 2.5794 | 0.0000 | 0.0000 | 1.1048 | 1.1770 | down | up |
| 442 | pos_2033 | PE(14:0/14:1(9Z)) | 0.1836 | 4.1823 | 0.0000 | 0.0000 | 1.1484 | 1.2359 | down | up |
| 443 | pos_2084 | C20915 | 0.1025 | 7.4520 | 0.0000 | 0.0000 | 1.1345 | 1.2299 | down | up |
| 444 | pos_2104 | Dihydroethidium | 0.3282 | 3.0028 | 0.0000 | 0.0000 | 1.1478 | 1.2415 | down | up |
| 445 | pos_2126 | Tryprostatin B | 0.5000 | 2.2690 | 0.0000 | 0.0000 | 1.1355 | 1.2377 | down | up |
| 446 | pos_2149 | 1-(9Z-hexadecenoyl)-glycero-3-phosphate | 0.2893 | 3.0008 | 0.0000 | 0.0000 | 1.1394 | 1.2337 | down | up |
| 447 | pos_2163 | 2,7-Diamino-7-iminoheptanoic acid | 0.0639 | 20.0094 | 0.0005 | 0.0002 | 1.0421 | 1.1555 | down | up |
| 448 | pos_2204 | n-Octanamide | 2.3338 | 0.8364 | 0.0000 | 0.0003 | 1.1421 | 1.1424 | up | down |
| 449 | pos_2217 | Metaldehyde | 0.3021 | 3.2118 | 0.0000 | 0.0000 | 1.1396 | 1.2161 | down | up |
| 450 | pos_2231 | Centhaquine | 0.1333 | 7.1121 | 0.0000 | 0.0000 | 1.1459 | 1.2392 | down | up |
| 451 | pos_2234 | [6]-Gingerdiol 5-acetate | 0.1255 | 14.8807 | 0.0000 | 0.0000 | 1.1490 | 1.2415 | down | up |
| 452 | pos_2240 | Pheniramine | 0.2724 | 3.7764 | 0.0000 | 0.0000 | 1.1384 | 1.2280 | down | up |
| 453 | pos_2298 | L-Tryptophanamide hydrochloride | 0.3115 | 3.1867 | 0.0000 | 0.0000 | 1.1403 | 1.2191 | down | up |
| 454 | pos_2299 | gamma-Humulene | 0.2305 | 3.7293 | 0.0000 | 0.0000 | 1.1260 | 1.2242 | down | up |
| 455 | pos_2300 | Minaprine | 0.3184 | 3.0382 | 0.0000 | 0.0000 | 1.1479 | 1.2380 | down | up |
| 456 | pos_2304 | Triethylene Glycol Monomethyl Ether | 0.3227 | 3.0003 | 0.0000 | 0.0000 | 1.1266 | 1.2212 | down | up |
| 457 | pos_2307 | Withaferin A | 0.2926 | 2.8972 | 0.0000 | 0.0000 | 1.1471 | 1.2394 | down | up |
| 458 | pos_2308 | PE(18:3(6Z,9Z,12Z)/20:5(5Z,8Z,11Z,14Z,17Z)) | 0.0678 | 13.0695 | 0.0000 | 0.0000 | 1.1315 | 1.2259 | down | up |
| 459 | pos_2309 | 13-hdohe | 3.1593 | 0.6638 | 0.0000 | 0.0000 | 1.1469 | 1.2310 | up | down |
| 460 | pos_2329 | Cuscohygrine | 0.2973 | 3.4762 | 0.0000 | 0.0000 | 1.1274 | 1.2155 | down | up |
| 461 | pos_2381 | lpe(0-0_20-4) | 0.2465 | 3.6111 | 0.0000 | 0.0000 | 1.1433 | 1.2348 | down | up |
| 462 | pos_2383 | Erinacine P | 0.2233 | 3.8285 | 0.0000 | 0.0000 | 1.1457 | 1.2374 | down | up |
| 463 | pos_2386 | 4-hydroxy-4-(pyridin-2-yl)butan-2-one | 0.0000 | 297519620.4569 | 0.0002 | 0.0018 | 1.0906 | 1.0996 | down | up |
| 464 | pos_2387 | Pentaethylene glycol | 0.3047 | 3.2268 | 0.0000 | 0.0000 | 1.1477 | 1.2300 | down | up |
| 465 | pos_2403 | Cer(d18:1/LTE4) | 0.4136 | 2.4782 | 0.0000 | 0.0000 | 1.1031 | 1.1858 | down | up |
| 466 | pos_2433 | (S)-Ureidoglycine | 0.4709 | 1.7260 | 0.0000 | 0.0000 | 1.1432 | 1.2249 | down | up |
| 467 | pos_2434 | L-Oxalylalbizziine | 0.3669 | 2.2093 | 0.0000 | 0.0000 | 1.0993 | 1.2081 | down | up |
| 468 | pos_2448 | DG(16:0/PGJ2/0:0) | 0.4767 | 1.8370 | 0.0000 | 0.0001 | 1.1113 | 1.1616 | down | up |
| 469 | pos_25 | Delavirdine | 4.5645 | 0.4503 | 0.0000 | 0.0000 | 1.1479 | 1.2346 | up | down |
| 470 | pos_2539 | Avermectin B1a aglycone | 0.2439 | 3.7474 | 0.0000 | 0.0000 | 1.1476 | 1.2406 | down | up |
| 471 | pos_2543 | Nandrolone phenpropionate | 0.0970 | 2.6893 | 0.0000 | 0.0000 | 1.1509 | 1.2403 | down | up |
| 472 | pos_2551 | 5-(3-Pyridyl)-2-hydroxytetrahydrofuran | 2.0979 | 0.7343 | 0.0000 | 0.0000 | 1.1460 | 1.2220 | up | down |
| 473 | pos_2553 | 3-Indoleacetic Acid | 2.3175 | 0.7206 | 0.0000 | 0.0000 | 1.1443 | 1.2062 | up | down |
| 474 | pos_26 | Antiarrhythmic peptide | 3.9873 | 0.5830 | 0.0000 | 0.0000 | 1.1492 | 1.2341 | up | down |
| 475 | pos_2624 | (S)-3-Mercaptohexyl pentanoate | 0.4605 | 2.0785 | 0.0000 | 0.0000 | 1.1427 | 1.2364 | down | up |
| 476 | pos_2641 | (1S,2R,4R,8S)-p-Menthane-2,8,9-triol 2-glucoside | 0.1631 | 4.7092 | 0.0000 | 0.0000 | 1.1485 | 1.2373 | down | up |
| 477 | pos_2653 | (5Z)-7-[(1R,2R,3R)-3-Hydroxy-2-[(1E,3S)-3-hydroxy-5-phenylpent-1-en-1-yl]-5-oxocyclopentyl]hept-5-enoylcarnitine | 0.2585 | 3.4861 | 0.0000 | 0.0000 | 1.1160 | 1.1899 | down | up |
| 478 | pos_2689 | all-trans-4-Hydroxyretinoic acid | 0.2422 | 4.0749 | 0.0000 | 0.0000 | 1.1479 | 1.2412 | down | up |
| 479 | pos_27 | Threoninyl-Tryptophan | 363.3177 | 0.3582 | 0.0000 | 0.0000 | 1.1446 | 1.2197 | up | down |
| 480 | pos_2741 | 5,7-Megastigmadien-9-ol glucoside | 2.0353 | 0.5943 | 0.0000 | 0.0000 | 1.1359 | 1.2098 | up | down |
| 481 | pos_2791 | 7-[(1R,2R,3R)-3-Hydroxy-2-[(3S)-3-hydroxyoctyl]-5-oxocyclopentyl]heptanoylcarnitine | 0.1863 | 4.1329 | 0.0000 | 0.0001 | 1.1065 | 1.1630 | down | up |
| 482 | pos_2801 | Val Val Val Phe | 0.3205 | 2.8933 | 0.0000 | 0.0000 | 1.1447 | 1.2395 | down | up |
| 483 | pos_2805 | 2-(2-(4-(2-Fluoroethoxy)phenyl)-5,7-dimethylpyrazolo(1,5-a)pyrimidin-3-yl)-N,N-diethylacetamide | 0.0099 | 96.9341 | 0.0000 | 0.0000 | 1.1450 | 1.2151 | down | up |
| 484 | pos_2807 | Sarcodon scabrosus Depsipeptide | 0.3712 | 2.4150 | 0.0000 | 0.0000 | 1.1486 | 1.2406 | down | up |
| 485 | pos_2817 | p-Coumaroylagmatine | 0.2898 | 2.9227 | 0.0000 | 0.0000 | 1.1444 | 1.2384 | down | up |
| 486 | pos_2836 | Coutaric acid | 0.2526 | 3.9713 | 0.0000 | 0.0000 | 1.1481 | 1.2413 | down | up |
| 487 | pos_2842 | 3-(7'-Methylthio)heptylmalic acid | 10.3500 | 0.6804 | 0.0000 | 0.0000 | 1.1428 | 1.1668 | up | down |
| 488 | pos_2850 | Daidzein | 40.9614 | 0.1452 | 0.0000 | 0.0000 | 1.1509 | 1.2419 | up | down |
| 489 | pos_2878 | 348281274 | 0.3109 | 2.6306 | 0.0000 | 0.0000 | 1.1457 | 1.2392 | down | up |
| 490 | pos_2901 | 3-amino-2-naphthoic acid | 0.4795 | 3.5343 | 0.0000 | 0.0000 | 1.1481 | 1.2390 | down | up |
| 491 | pos_2921 | Milbemycin A4 | 0.3418 | 2.8450 | 0.0000 | 0.0000 | 1.1487 | 1.2409 | down | up |
| 492 | pos_2924 | Antibiotic JI-20B | 0.3843 | 2.3965 | 0.0000 | 0.0000 | 1.1405 | 1.2271 | down | up |
| 493 | pos_2935 | Taurocholate | 0.3745 | 2.1329 | 0.0000 | 0.0000 | 1.1470 | 1.2368 | down | up |
| 494 | pos_2940 | Milbemycin A3 | 0.3431 | 2.6706 | 0.0000 | 0.0000 | 1.1458 | 1.2366 | down | up |
| 495 | pos_2956 | Denbufylline | 0.3234 | 2.5895 | 0.0000 | 0.0000 | 1.1445 | 1.2400 | down | up |
| 496 | pos_2978 | Vignatic acid B | 0.4099 | 2.3340 | 0.0000 | 0.0000 | 1.1455 | 1.2386 | down | up |
| 497 | pos_2992 | Wogonin | 18.3767 | 0.1692 | 0.0000 | 0.0000 | 1.1506 | 1.2414 | up | down |
| 498 | pos_3076 | 2,2'-(3-methylcyclohexane-1,1-diyl)diacetic acid | 0.4432 | 1.8933 | 0.0000 | 0.0000 | 1.1460 | 1.2350 | down | up |
| 499 | pos_3083 | Dethiobiotin | 0.1791 | 2.8698 | 0.0000 | 0.0001 | 1.1437 | 1.1713 | down | up |
| 500 | pos_3095 | Phosphatidylethanolamine (16_1_18_3) Abbr_ PoLnPE | 0.2599 | 2.9678 | 0.0000 | 0.0000 | 1.1318 | 1.2173 | down | up |
| 501 | pos_3102 | Isoquinoline | 0.3282 | 3.3071 | 0.0000 | 0.0000 | 1.1500 | 1.2417 | down | up |
| 502 | pos_3103 | 3-Indolepropionic acid | 0.3681 | 2.9405 | 0.0000 | 0.0000 | 1.1481 | 1.2410 | down | up |
| 503 | pos_3105 | Descarbonyl-lacosamide | 0.1220 | 1.6425 | 0.0000 | 0.0001 | 1.1473 | 1.1177 | down | up |
| 504 | pos_3142 | Verdamicin | 0.2575 | 2.3285 | 0.0000 | 0.0003 | 1.1152 | 1.0935 | down | up |
| 505 | pos_3168 | 1,2,3,4,5,6-Hexahydro-5-(1-hydroxyethylidene)-7H-cyclopenta[b]pyridin-7-one | 339.9874 | 0.0000 | 0.0000 | 0.0000 | 1.1470 | 1.2378 | up | down |
| 506 | pos_3185 | (2E,6E,11E,13E)-18-(2,6-dioxopiperidin-4-yl)-9-hydroxy-8-methoxy-10,12,14-trimethyl-15-oxooctadeca-2,6,11,13-tetraenoic acid | 0.0647 | 2.2382 | 0.0000 | 0.0000 | 1.1505 | 1.2227 | down | up |
| 507 | pos_3226 | 1-(13Z,16Z-docosadienoyl)-glycero-3-phosphate | 0.4664 | 1.4828 | 0.0000 | 0.0000 | 1.1339 | 1.1565 | down | up |
| 508 | pos_3304 | PS(18:2(9Z,12Z)/TXB2) | 0.4143 | 2.1371 | 0.0000 | 0.0000 | 1.1488 | 1.2402 | down | up |
| 509 | pos_3313 | Ginsenoside Rb1 | 5.7902 | 0.6928 | 0.0000 | 0.0002 | 1.1395 | 1.1556 | up | down |
| 510 | pos_3322 | Chavicol | 0.0014 | 41.1933 | 0.0000 | 0.0000 | 1.1474 | 1.2058 | down | up |
| 511 | pos_3324 | Isoelemicin | 0.0092 | 8.1686 | 0.0000 | 0.0000 | 1.1483 | 1.2366 | down | up |
| 512 | pos_3326 | 12alpha-Hydroxy-13,18-dehydroparain | 0.0217 | 4.4533 | 0.0000 | 0.0000 | 1.1487 | 1.2323 | down | up |
| 513 | pos_3507 | Vesatolimod | 635681018.9399 | 0.2842 | 0.0000 | 0.0000 | 1.1485 | 1.2329 | up | down |
| 514 | pos_3533 | Abscisic aldehyde | 0.3212 | 2.5056 | 0.0000 | 0.0000 | 1.1475 | 1.2402 | down | up |
| 515 | pos_3546 | Linatine | 2.0346 | 0.4916 | 0.0000 | 0.0000 | 1.1219 | 1.2115 | up | down |
| 516 | pos_3580 | 1,4,7,10,13,16-Hexaoxacyclooctadecane | 0.4317 | 2.5149 | 0.0000 | 0.0000 | 1.1450 | 1.2351 | down | up |
| 517 | pos_3581 | 2,6-Dimethyl-7-octene-1,6-diol 8-O-glucoside | 0.3401 | 2.3665 | 0.0000 | 0.0000 | 1.1447 | 1.2352 | down | up |
| 518 | pos_3585 | Ganoderic acid Mg | 0.3987 | 1.4590 | 0.0000 | 0.0003 | 1.1369 | 1.0925 | down | up |
| 519 | pos_3603 | 3-(1-Propyl-3-piperidinyl)phenol | 0.4865 | 1.6586 | 0.0000 | 0.0000 | 1.1382 | 1.2156 | down | up |
| 520 | pos_3608 | 5-Oxopentanoate | 20.4749 | 0.0754 | 0.0000 | 0.0000 | 1.1508 | 1.2418 | up | down |
| 521 | pos_3616 | DG(2:0/PGD1/0:0) | 0.3360 | 2.5982 | 0.0000 | 0.0000 | 1.1229 | 1.1902 | down | up |
| 522 | pos_3627 | Arg Trp Lys Leu | 0.3818 | 2.1084 | 0.0000 | 0.0000 | 1.1467 | 1.2357 | down | up |
| 523 | pos_3632 | Glutathionylspermine | 0.4175 | 1.4965 | 0.0000 | 0.0004 | 1.1173 | 1.0983 | down | up |
| 524 | pos_3640 | Butanedioic acid, octenyl- | 0.4098 | 1.9785 | 0.0000 | 0.0000 | 1.1414 | 1.2313 | down | up |
| 525 | pos_3650 | Retinyl beta-glucuronide | 0.1345 | 2.3034 | 0.0000 | 0.0000 | 1.1438 | 1.1559 | down | up |
| 526 | pos_3653 | (2S,3R,4R)-2-Amino-3,4-dihydroxy-2-(hydroxymethyl)-14-oxoicos-6-enoic acid | 2.7418 | 0.5543 | 0.0000 | 0.0000 | 1.1413 | 1.2193 | up | down |
| 527 | pos_3658 | (R)-(Indol-3-yl)lactate | 0.4070 | 3.9995 | 0.0000 | 0.0000 | 1.1382 | 1.2407 | down | up |
| 528 | pos_3697 | 3-Phenyl-2-propenenitrile | 0.1633 | 6.6683 | 0.0000 | 0.0000 | 1.1487 | 1.2398 | down | up |
| 529 | pos_3704 | Vinyl butyrate | 0.3117 | 2.5617 | 0.0000 | 0.0000 | 1.1347 | 1.2219 | down | up |
| 530 | pos_3705 | Malvidin | 19.4210 | 0.0000 | 0.0000 | 0.0000 | 1.1365 | 1.2299 | up | down |
| 531 | pos_3706 | (2S,3R,4R,5R,6R)-2-methyl-6-[[(2R,3S,4S,5R,6R)-3,4,5-trihydroxy-6-[(E)-7-hydroxy-3,7-dimethyloct-3-enoxy]oxan-2-yl]methoxy]oxane-3,4,5-triol | 0.2987 | 2.6468 | 0.0000 | 0.0000 | 1.1398 | 1.2049 | down | up |
| 532 | pos_3711 | N-[3-(1,3-Dioxoisoindol-2-yl)propyl]-2,2,5,5-tetramethyl-1H-pyrrole-3-carboxamide | 0.0140 | 97.6531 | 0.0000 | 0.0000 | 1.1346 | 1.2228 | down | up |
| 533 | pos_3712 | Cys Lys Arg Ser | 0.0484 | 27.4354 | 0.0000 | 0.0000 | 1.1498 | 1.2387 | down | up |
| 534 | pos_3755 | NCGC00381111-01_C23H38N4O6_1-[(2E,4E)-6,7-Dihydroxy-2,4-octadienoyl]prolyl-N-methylvalyl-N~2~-methylalaninamide | 0.3280 | 2.2967 | 0.0000 | 0.0000 | 1.1442 | 1.2364 | down | up |
| 535 | pos_3769 | Delta-Hexalactone | 0.3646 | 1.9960 | 0.0000 | 0.0000 | 1.1461 | 1.2160 | down | up |
| 536 | pos_3770 | Cyclohexyl cinnamate | 4.6470 | 0.8078 | 0.0000 | 0.0000 | 1.1494 | 1.2047 | up | down |
| 537 | pos_3775 | Acetylpterosin C | 4.2020 | 0.7989 | 0.0000 | 0.0000 | 1.1500 | 1.2248 | up | down |
| 538 | pos_3776 | Pseudaminic acid | 5.5932 | 0.7878 | 0.0000 | 0.0000 | 1.1497 | 1.2170 | up | down |
| 539 | pos_3813 | Thyroxine | 0.3693 | 1.4488 | 0.0000 | 0.0000 | 1.1481 | 1.2230 | down | up |
| 540 | pos_3823 | Seryllysine | 0.4056 | 1.9148 | 0.0000 | 0.0000 | 1.1402 | 1.2190 | down | up |
| 541 | pos_3834 | SM(d18:0/LTE4) | 0.4783 | 1.4783 | 0.0000 | 0.0003 | 1.1155 | 1.0787 | down | up |
| 542 | pos_3842 | Zotarolimus | 0.4569 | 1.6044 | 0.0000 | 0.0000 | 1.0999 | 1.1461 | down | up |
| 543 | pos_3848 | PE(DiMe(9,3)/20:5(7Z,9Z,11E,13E,17Z)-3OH(5,6,15)) | 0.3545 | 1.3884 | 0.0000 | 0.0000 | 1.1488 | 1.1550 | down | up |
| 544 | pos_3851 | glu-leu-arg | 0.4674 | 1.5058 | 0.0000 | 0.0003 | 1.1119 | 1.0818 | down | up |
| 545 | pos_3857 | Jurubine | 2.1088 | 0.7582 | 0.0000 | 0.0000 | 1.1447 | 1.2097 | up | down |
| 546 | pos_3872 | Gln Phe Gln Tyr | 0.3649 | 1.3975 | 0.0000 | 0.0000 | 1.1477 | 1.2231 | down | up |
| 547 | pos_3914 | Lisinopril, epsilon-biotinamidocaproyl- | 0.0174 | 6.1779 | 0.0000 | 0.0000 | 1.1470 | 1.2345 | down | up |
| 548 | pos_3932 | Avermectin A1a aglycone | 0.4566 | 1.7929 | 0.0000 | 0.0000 | 1.1462 | 1.2373 | down | up |
| 549 | pos_4100 | 31-Nordehydrolanosterol | 2.0487 | 0.7915 | 0.0000 | 0.0001 | 1.1325 | 1.1092 | up | down |
| 550 | pos_4122 | Capillene | 5.7082 | 0.7918 | 0.0000 | 0.0000 | 1.1453 | 1.1612 | up | down |
| 551 | pos_4125 | Pilocarpic acid | 357877026.7227 | 0.7694 | 0.0000 | 0.0018 | 1.1404 | 1.0047 | up | down |
| 552 | pos_4126 | C20914 | 5.4793 | 0.7499 | 0.0000 | 0.0006 | 1.1465 | 1.1207 | up | down |
| 553 | pos_4130 | 2-[(1E,3E,5E)-3,7-Dimethylnona-1,3,5,8-tetraenyl]-1,3,3-trimethylcyclohexene | 5.0828 | 0.8184 | 0.0000 | 0.0029 | 1.1365 | 1.0265 | up | down |
| 554 | pos_4136 | Glycocholic Acid | 7.6125 | 0.7958 | 0.0000 | 0.0000 | 1.1489 | 1.1797 | up | down |
| 555 | pos_4146 | Methyl 11,17,21-trihydroxy-3,20-dioxopregna-1,4-diene-16-carboxylate | 0.0029 | 11.8435 | 0.0000 | 0.0015 | 1.1468 | 1.0713 | down | up |
| 556 | pos_4155 | Persicachrome | 3.6978 | 0.7674 | 0.0000 | 0.0000 | 1.1436 | 1.1683 | up | down |
| 557 | pos_4157 | Deoxycholylserine | 4.1581 | 0.8191 | 0.0000 | 0.0000 | 1.1475 | 1.1750 | up | down |
| 558 | pos_4186 | 1-Deoxy-11beta-hydroxypentalenate | 0.2029 | 1.8516 | 0.0000 | 0.0000 | 1.1465 | 1.2210 | down | up |
| 559 | pos_4199 | 5'-Methylthioadenosine | 0.3553 | 1.6288 | 0.0000 | 0.0000 | 1.1455 | 1.1916 | down | up |
| 560 | pos_4230 | Docosapentaenoic acid (22n-6) | 0.2429 | 1.4378 | 0.0000 | 0.0004 | 1.1485 | 1.0793 | down | up |
| 561 | pos_4246 | 3-Ethyl-5-methylphenol | 4.7361 | 0.5050 | 0.0000 | 0.0000 | 1.1482 | 1.2311 | up | down |
| 562 | pos_4247 | 3-Phenylpropyl formate | 4.1378 | 0.4877 | 0.0000 | 0.0000 | 1.1489 | 1.2363 | up | down |
| 563 | pos_4249 | Tocopheronic acid | 5.5862 | 0.5009 | 0.0000 | 0.0000 | 1.1429 | 1.2179 | up | down |
| 564 | pos_4250 | Pyrenophorol | 6.8747 | 0.4922 | 0.0000 | 0.0000 | 1.1497 | 1.2363 | up | down |
| 565 | pos_4251 | Methionyl-Lysine | 4.7584 | 0.4959 | 0.0000 | 0.0000 | 1.1459 | 1.2275 | up | down |
| 566 | pos_4264 | Antibiotic JI-20A | 0.4825 | 1.1170 | 0.0000 | 0.0000 | 1.1498 | 1.2010 | down | up |
| 567 | pos_4266 | Tuftsin | 0.3184 | 2.2139 | 0.0000 | 0.0000 | 1.1415 | 1.2198 | down | up |
| 568 | pos_4317 | Lyciumoside II | 3.0669 | 0.8421 | 0.0000 | 0.0003 | 1.1429 | 1.0760 | up | down |
| 569 | pos_4328 | Adhumulinic acid | 0.3895 | 2.1353 | 0.0000 | 0.0000 | 1.1166 | 1.1883 | down | up |
| 570 | pos_4350 | Erythronolide B | 4.8513 | 0.6642 | 0.0000 | 0.0002 | 1.1312 | 1.1388 | up | down |
| 571 | pos_4364 | Butaprost | 2.7024 | 0.7434 | 0.0000 | 0.0000 | 1.1388 | 1.1705 | up | down |
| 572 | pos_4380 | Acoric acid | 0.0297 | 47.4910 | 0.0000 | 0.0000 | 1.1465 | 1.2374 | down | up |
| 573 | pos_4456 | 7-Ethyl-5,6-dihydro-1,4-dimethylazulene | 0.1698 | 4.0787 | 0.0000 | 0.0000 | 1.1460 | 1.2338 | down | up |
| 574 | pos_4508 | Senkyunolide N | 0.0066 | 26.6386 | 0.0008 | 0.0055 | 1.0529 | 1.0231 | down | up |
| 575 | pos_4537 | Propylparaben | 0.0507 | 20.1919 | 0.0000 | 0.0000 | 1.1388 | 1.2321 | down | up |
| 576 | pos_4538 | 2-{[(1r,2s)-2-Aminocyclohexyl]amino}-4-{[3-(2h-1,2,3-Triazol-2-Yl)phenyl]amino}pyrimidine-5-Carboxamide | 0.0114 | 51.3159 | 0.0000 | 0.0000 | 1.1442 | 1.2399 | down | up |
| 577 | pos_4540 | (Z)-3-Oxo-2-(2-pentenyl)-1-cyclopenteneacetic acid | 0.0275 | 34.3949 | 0.0000 | 0.0000 | 1.1408 | 1.2344 | down | up |
| 578 | pos_4613 | Mauritine A | 3.1765 | 0.5875 | 0.0000 | 0.0000 | 1.1411 | 1.1917 | up | down |
| 579 | pos_4654 | 1,2-Dihydro-1,1,6-trimethylnaphthalene | 2.5691 | 0.4482 | 0.0000 | 0.0000 | 1.0906 | 1.1858 | up | down |
| 580 | pos_4656 | (4E,7E,10E,13E)-Hexadeca-4,7,10,13-tetraenoic acid | 2.4704 | 0.4988 | 0.0000 | 0.0000 | 1.1486 | 1.2288 | up | down |
| 581 | pos_4659 | (10Z,14E,16E)-10,14,16-Octadecatrien-12-ynoic acid | 2.4743 | 0.4930 | 0.0000 | 0.0000 | 1.1425 | 1.2253 | up | down |
| 582 | pos_4661 | 18-Nor-4(19),8,11,13-abietatetraene | 2.8330 | 0.4221 | 0.0000 | 0.0000 | 1.1327 | 1.2138 | up | down |
| 583 | pos_4662 | Virol A | 2.0791 | 0.5135 | 0.0000 | 0.0000 | 1.1326 | 1.2250 | up | down |
| 584 | pos_4663 | Eremolactone | 2.4014 | 0.4869 | 0.0000 | 0.0000 | 1.1259 | 1.1817 | up | down |
| 585 | pos_4664 | Dehydroabietic acid | 2.8358 | 0.4507 | 0.0000 | 0.0000 | 1.0841 | 1.1445 | up | down |
| 586 | pos_4681 | Nodularin-R | 2.8813 | 0.5029 | 0.0000 | 0.0000 | 1.1106 | 1.1428 | up | down |
| 587 | pos_473 | Nitroxoline | 0.4584 | 1.1785 | 0.0000 | 0.0000 | 1.1499 | 1.2145 | down | up |
| 588 | pos_4755 | Nvp-aew541 | 0.2235 | 7.4715 | 0.0000 | 0.0000 | 1.1157 | 1.2326 | down | up |
| 589 | pos_4770 | 3a,7b,12a-Trihydroxyoxocholanyl-Glycine | 2.3328 | 0.7415 | 0.0000 | 0.0000 | 1.1406 | 1.2006 | up | down |
| 590 | pos_4844 | Enterodiol sulfate | 2.1056 | 0.4313 | 0.0000 | 0.0000 | 1.1455 | 1.2374 | up | down |
| 591 | pos_4866 | Reichstein's substance E | 12.7892 | 0.7342 | 0.0000 | 0.0000 | 1.1488 | 1.1977 | up | down |
| 592 | pos_4925 | Gentamicin X2 | 29.2943 | 0.4351 | 0.0000 | 0.0000 | 1.1482 | 1.2334 | up | down |
| 593 | pos_4949 | Zearalanone | 0.2952 | 3.6307 | 0.0000 | 0.0000 | 1.1415 | 1.2378 | down | up |
| 594 | pos_4964 | Calendic acid | 2.2518 | 0.8240 | 0.0000 | 0.0001 | 1.1150 | 1.1129 | up | down |
| 595 | pos_4965 | Methyl cis-p-coumarate 3-(3,7-dimethyl-2,6-octadienyl) | 0.4638 | 1.8626 | 0.0000 | 0.0000 | 1.1244 | 1.1957 | down | up |
| 596 | pos_5041 | 2',4',6',3-Tetrahydroxy-3'-geranyl-6'',6''-dimethylpyrano[2'',3'':4,5]dihydrochalcone | 0.2132 | 1.3168 | 0.0000 | 0.0001 | 1.1496 | 1.1487 | down | up |
| 597 | pos_5042 | Capsianoside I | 0.0232 | 2.2767 | 0.0000 | 0.0000 | 1.1458 | 1.1917 | down | up |
| 598 | pos_5132 | PC(14:0/0:0) | 0.4811 | 1.2825 | 0.0000 | 0.0000 | 1.1502 | 1.2210 | down | up |
| 599 | pos_5137 | Bouillonamide A | 2.2985 | 0.4471 | 0.0000 | 0.0000 | 1.1207 | 1.1752 | up | down |
| 600 | pos_5159 | 7E,9E,11-Dodecatrienyl acetate | 6.9487 | 0.8591 | 0.0000 | 0.0008 | 1.1442 | 1.0559 | up | down |
| 601 | pos_5238 | Oleandomycin triacetate | 2.3752 | 0.4172 | 0.0000 | 0.0001 | 1.0476 | 1.1260 | up | down |
| 602 | pos_5287 | 28-Norcastasterone | 2.5069 | 0.6720 | 0.0000 | 0.0000 | 1.1451 | 1.2082 | up | down |
| 603 | pos_5330 | Validoxylamine A | 5.7399 | 0.6963 | 0.0000 | 0.0000 | 1.1493 | 1.2246 | up | down |
| 604 | pos_5373 | 23S,25,26-Trihydroxyvitamin D3 | 2.3474 | 0.8350 | 0.0000 | 0.0009 | 1.1421 | 1.0346 | up | down |
| 605 | pos_5382 | PE(14:0/PGJ2) | 2.5974 | 0.4256 | 0.0000 | 0.0000 | 1.1189 | 1.1534 | up | down |
| 606 | pos_5507 | Octadecenoylcarnitine | 0.0202 | 2.9657 | 0.0000 | 0.0000 | 1.1467 | 1.1826 | down | up |
| 607 | pos_5528 | Alpha-Linoleoylcholine | 0.0279 | 3.6960 | 0.0000 | 0.0000 | 1.1494 | 1.2241 | down | up |
| 608 | pos_56 | Urolithin B 3-O-glucuronide | 2.0340 | 0.8133 | 0.0000 | 0.0016 | 1.1206 | 1.0053 | up | down |
| 609 | pos_562 | Norvaline | 0.4922 | 1.6092 | 0.0000 | 0.0000 | 1.1476 | 1.2369 | down | up |
| 610 | pos_5664 | 6,8a-Seco-6,8a-deoxy-5-oxoavermectin ''1a'' aglycone | 2.1355 | 0.6566 | 0.0000 | 0.0000 | 1.1201 | 1.1917 | up | down |
| 611 | pos_578 | Ethyl glucuronide | 2.9490 | 0.6189 | 0.0000 | 0.0000 | 1.1497 | 1.2393 | up | down |
| 612 | pos_583 | S-Adenosylmethioninamine | 4.8930 | 0.7027 | 0.0000 | 0.0000 | 1.1479 | 1.2148 | up | down |
| 613 | pos_586 | Streptidine 6-phosphate | 3.5682 | 0.6860 | 0.0000 | 0.0000 | 1.1493 | 1.2324 | up | down |
| 614 | pos_598 | Cytosine | 4.1995 | 0.5256 | 0.0000 | 0.0000 | 1.1477 | 1.2321 | up | down |
| 615 | pos_6 | Leu Thr | 3.1894 | 0.3282 | 0.0000 | 0.0000 | 1.1426 | 1.2320 | up | down |
| 616 | pos_600 | Trifluridine | 5.8566 | 0.3175 | 0.0000 | 0.0000 | 1.1342 | 1.2125 | up | down |
| 617 | pos_6038 | 6-[5]-ladderane-1-hexanol | 2.3066 | 0.5030 | 0.0003 | 0.0000 | 1.0297 | 1.1689 | up | down |
| 618 | pos_605 | 7-Methyluric acid | 2.0598 | 0.7025 | 0.0000 | 0.0000 | 1.1323 | 1.1517 | up | down |
| 619 | pos_6054 | Corchorifatty acid D | 2.3226 | 0.5848 | 0.0000 | 0.0007 | 1.0763 | 1.0376 | up | down |
| 620 | pos_61 | Loganin | 0.2849 | 2.3746 | 0.0000 | 0.0000 | 1.1120 | 1.1379 | down | up |
| 621 | pos_6151 | Cis-8,11,14,17-Eicosatetraenoic acid | 0.4382 | 1.7281 | 0.0000 | 0.0000 | 1.1444 | 1.1957 | down | up |
| 622 | pos_6181 | Helicallenal | 0.3481 | 1.8020 | 0.0000 | 0.0000 | 1.1399 | 1.2179 | down | up |
| 623 | pos_621 | Levan | 14.2349 | 0.3929 | 0.0000 | 0.0000 | 1.1493 | 1.2368 | up | down |
| 624 | pos_6221 | (3R,5S,6R,7R,8S,9S,10S,13R,14S,17R)-6-Ethyl-17-((R)-4-hydroxybutan-2-yl)-10,13-dimethylhexadecahydro-1H-cyclopenta[a]phenanthrene-3,7-diol | 0.4502 | 1.1887 | 0.0000 | 0.0011 | 1.1462 | 1.0264 | down | up |
| 625 | pos_63 | Chitobiose | 0.3386 | 2.0474 | 0.0000 | 0.0000 | 1.1324 | 1.2236 | down | up |
| 626 | pos_634 | N-(1-Deoxy-1-fructosyl)proline | 2.7645 | 0.8268 | 0.0000 | 0.0000 | 1.1491 | 1.1989 | up | down |
| 627 | pos_6349 | Septacidin | 3.1075 | 0.8089 | 0.0000 | 0.0000 | 1.1503 | 1.2141 | up | down |
| 628 | pos_636 | Queuine | 2.8599 | 0.6983 | 0.0000 | 0.0000 | 1.1467 | 1.2238 | up | down |
| 629 | pos_642 | 2-Isopropyl-3-oxosuccinate | 2.1617 | 0.8991 | 0.0000 | 0.0010 | 1.1406 | 1.0578 | up | down |
| 630 | pos_661 | N2-Methyl-L-lysine | 4.1682 | 0.3481 | 0.0000 | 0.0000 | 1.1475 | 1.2353 | up | down |
| 631 | pos_6732 | Lipoteichoic acid | 6.6341 | 0.4425 | 0.0000 | 0.0005 | 1.0938 | 1.0553 | up | down |
| 632 | pos_680 | 2,3-Butanediol apiosylglucoside | 0.4736 | 1.7505 | 0.0000 | 0.0000 | 1.1256 | 1.2197 | down | up |
| 633 | pos_6908 | DG(15:0/6 keto-PGF1alpha/0:0) | 0.4940 | 1.6726 | 0.0000 | 0.0000 | 1.1387 | 1.2276 | down | up |
| 634 | pos_6918 | 16,16-Dimethylprostaglandin E2 | 2.0023 | 0.4322 | 0.0000 | 0.0000 | 1.0722 | 1.1703 | up | down |
| 635 | pos_6933 | Menthone 1,2-glyceryl ketal | 0.3503 | 2.6470 | 0.0000 | 0.0000 | 1.1189 | 1.1919 | down | up |
| 636 | pos_695 | Hydroxypropionylcarnitine | 10.1451 | 0.7726 | 0.0000 | 0.0001 | 1.1476 | 1.1833 | up | down |
| 637 | pos_698 | N-(1-Deoxy-1-fructosyl)valine | 11.3665 | 0.8161 | 0.0000 | 0.0001 | 1.1481 | 1.1608 | up | down |
| 638 | pos_700 | Triphenylpropargylphosphonium | 11.0756 | 0.7954 | 0.0000 | 0.0000 | 1.1490 | 1.1952 | up | down |
| 639 | pos_701 | Fructosyl valine | 15.5049 | 0.7212 | 0.0000 | 0.0000 | 1.1475 | 1.1940 | up | down |
| 640 | pos_7032 | pe(20-4(5z,8z,11z,14z)_p-18-1(11z)) | 0.4869 | 2.4088 | 0.0000 | 0.0000 | 1.1468 | 1.2384 | down | up |
| 641 | pos_7087 | PE(20:4(8Z,11Z,14Z,17Z)/18:2(10E,12Z)+=O(9)) | 2.4468 | 0.4323 | 0.0003 | 0.0005 | 1.0528 | 1.1337 | up | down |
| 642 | pos_71 | Lactose-lysine | 3.2636 | 0.6515 | 0.0000 | 0.0000 | 1.1469 | 1.2207 | up | down |
| 643 | pos_741 | Protopine | 3.6586 | 0.7331 | 0.0000 | 0.0002 | 1.1333 | 1.0945 | up | down |
| 644 | pos_748 | Cyanidin 3-(6''-acetylglucoside) | 10.1723 | 0.8506 | 0.0000 | 0.0001 | 1.1487 | 1.1304 | up | down |
| 645 | pos_76 | S-(Hydroxyphenylacetothiohydroximoyl)-L-cysteine | 0.2525 | 2.4712 | 0.0000 | 0.0000 | 1.1414 | 1.2067 | down | up |
| 646 | pos_7760 | N,N-Dimethylsphingosine | 0.4442 | 1.7703 | 0.0000 | 0.0009 | 1.1241 | 1.0721 | down | up |
| 647 | pos_785 | Acetyl-arginyl-glycyl-aspartyl-serinamide | 0.3990 | 1.4031 | 0.0000 | 0.0008 | 1.1256 | 1.0401 | down | up |
| 648 | pos_7853 | 2-aminoethyl [3-[(Z)-heptadec-9-enoxy]-2-hydroxypropyl] hydrogen phosphate | 0.4763 | 2.1263 | 0.0000 | 0.0001 | 1.1408 | 1.1797 | down | up |
| 649 | pos_7882 | 1-Phenyl-1,3-eicosanedione | 0.1652 | 6.3126 | 0.0000 | 0.0000 | 1.0886 | 1.1896 | down | up |
| 650 | pos_8055 | 17-Hydroxylinolenic acid | 3.3285 | 0.3590 | 0.0001 | 0.0001 | 1.1077 | 1.1854 | up | down |
| 651 | pos_816 | 5-Hydroxyectoine | 0.4871 | 1.1538 | 0.0000 | 0.0000 | 1.1497 | 1.2058 | down | up |
| 652 | pos_8214 | LPE(O-18-2) | 2.7186 | 0.4007 | 0.0001 | 0.0001 | 1.1033 | 1.1843 | up | down |
| 653 | pos_825 | Arabinofuranobiose | 12.1830 | 0.4053 | 0.0000 | 0.0000 | 1.1504 | 1.2403 | up | down |
| 654 | pos_8299 | pc(24-0) | 2.9045 | 0.4194 | 0.0001 | 0.0001 | 1.1110 | 1.1843 | up | down |
| 655 | pos_84 | Isopentenyladenosine-5'-triphosphate | 0.2586 | 2.1649 | 0.0000 | 0.0000 | 1.1311 | 1.1692 | down | up |
| 656 | pos_849 | Dhurrin | 2.1252 | 0.7928 | 0.0000 | 0.0000 | 1.1492 | 1.2250 | up | down |
| 657 | pos_85 | GDP-L-fucose | 0.2733 | 2.2297 | 0.0000 | 0.0000 | 1.1163 | 1.2123 | down | up |
| 658 | pos_871 | cyclo-Dopa-glucuronylglucoside | 134.0378 | 0.4471 | 0.0000 | 0.0000 | 1.1448 | 1.2053 | up | down |
| 659 | pos_876 | p-Coumaric acid | 0.4880 | 1.2121 | 0.0000 | 0.0000 | 1.1471 | 1.1977 | down | up |
| 660 | pos_880 | (2R,3R,4R,5R)-2-Amino-4,5,6-trihydroxy-3-[(2R)-1-oxopropan-2-yl]oxyhexanal | 0.3731 | 1.2853 | 0.0000 | 0.0013 | 1.1259 | 1.0264 | down | up |
| 661 | pos_886 | (R)-3-hydroxybutyrylcarnitine | 7.3666 | 0.8073 | 0.0000 | 0.0000 | 1.1506 | 1.2277 | up | down |
| 662 | pos_887 | Guanidino arginine | 117.4338 | 0.6795 | 0.0000 | 0.0001 | 1.1469 | 1.1263 | up | down |
| 663 | pos_890 | N-Phenylacetylglutamic acid | 3.7687 | 0.8465 | 0.0000 | 0.0000 | 1.1494 | 1.2023 | up | down |
| 664 | pos_896 | N-Fructosyl isoleucine | 9.3141 | 0.8253 | 0.0000 | 0.0000 | 1.1506 | 1.2245 | up | down |
| 665 | pos_899 | Neosaxitoxin | 9.0627 | 0.8175 | 0.0000 | 0.0000 | 1.1506 | 1.2257 | up | down |
| 666 | pos_9 | 10-Nitrolinoleic acid | 3.8646 | 0.4140 | 0.0000 | 0.0000 | 1.1486 | 1.2370 | up | down |
| 667 | pos_913 | O-Desmethylvenlafaxine glucuronide | 385876508.3611 | 0.6540 | 0.0000 | 0.0004 | 1.1470 | 1.1017 | up | down |
| 668 | pos_928 | Nopaline | 656259566.3237 | 0.5146 | 0.0000 | 0.0000 | 1.1420 | 1.1929 | up | down |
| 669 | pos_929 | Stachyose | 485.2347 | 0.5976 | 0.0000 | 0.0001 | 1.1397 | 1.1626 | up | down |
| 670 | pos_932 | 6''-O-alpha-D-Galactopyranosylciceritol | 58.8271 | 0.6249 | 0.0000 | 0.0000 | 1.1486 | 1.2148 | up | down |
| 671 | pos_950 | Allyl crotonate | 0.4785 | 1.2993 | 0.0000 | 0.0000 | 1.1485 | 1.2240 | down | up |
| 672 | pos_958 | (R)-3-((R)-3-Hydroxybutanoyloxy)butanoate | 0.2289 | 1.6632 | 0.0000 | 0.0007 | 1.1181 | 1.0619 | down | up |
| 673 | pos_963 | 5-Hydroxypentanoylcarnitine | 2.7130 | 0.7529 | 0.0000 | 0.0000 | 1.1467 | 1.2159 | up | down |
